# An emergent disease-associated motor neuron state precedes cell death in ALS

**DOI:** 10.1016/j.cell.2026.05.047

**Published:** 2026-06-23

**Authors:** Olivia Gautier, Jacob A. Blum, Thao P. Nguyen, Shaolong Cao, Sandy Klemm, Mai Yamakawa, Dann Huh, Jessica A. Hurt, Nasa Sinnott-Armstrong, Yi Zeng, Chung-ha O. Davis, Juliane Bombosch, Chang Liu, Lisa N. Encarnacion, Kevin A. Guttenplan, Derek Chen, Arwa Kathiria, Luke Zhao, Stephen Moore, Alex Meng, Kailee Ong, Don W. Cleveland, John Ravits, Jessica E. Rexach, William J. Greenleaf, Aaron D. Gitler

**Affiliations:** 1 Department of Genetics, Stanford University School of Medicine, Stanford, CA 94305, USA; 2 Stanford Neurosciences Graduate Program, Stanford University School of Medicine, Stanford, CA 94305, USA; 3 Research, Biogen Inc., Cambridge, MA 02142, USA; 4 Program in Neurogenetics, Department of Neurology, David Geffen School of Medicine, University of California, Los Angeles, Los Angeles, CA 90095, USA; 5 Herbold Computational Biology Program, Public Health Sciences Division, Fred Hutchinson Cancer Center, Seattle, WA 98195, USA; 6 Department of Genome Sciences, University of Washington, Seattle, WA 98195, USA; 7 Brotman Baty Institute, University of Washington, Seattle, WA 98195, USA; 8 Department of Neurobiology, Stanford University, Stanford, CA 94305, USA; 9 Vollum Institute, Oregon Health and Sciences University, Portland, OR 97239, USA; 10 Departments of Cellular and Molecular Medicine, University of California, San Diego, La Jolla, CA 92093, USA; 11 Department of Neurosciences, University of California, San Diego, La Jolla, CA 92093, USA; 12 Department of Human Genetics, David Geffen School of Medicine, University of California, Los Angeles, Los Angeles, CA 90095, USA; 13 Department of Applied Physics, Stanford University, Stanford, CA 94305, USA; 14 Biohub – San Francisco, San Francisco, CA 94158, USA; 15 The Phil and Penny Knight Initiative for Brain Resilience, Stanford University, Stanford, CA 94305, USA; 16 These authors contributed equally; 17 Lead contact

## Abstract

To define molecular determinants of motor neuron degeneration in amyotrophic lateral sclerosis (ALS), we generated longitudinal single-nucleus transcriptomes and chromatin accessibility profiles of spinal motor neurons together with spatial transcriptomics from the SOD1-G93A mouse model. Vulnerable alpha motor neurons showed thousands of molecular changes, marking a transition into a distinct cell state we named “disease-associated motor neurons” (DMs). We identified transcription factor networks that govern how healthy cells transition into DMs and those associated with motor neuron subtype-selective vulnerability. Upregulation of DM-associated transcription factors in human motor neurons induced key features of DMs, demonstrating an active regulatory component. Human ALS spinal cord single-nucleus RNA sequencing data demonstrated conservation of the DM signature in alpha motor neurons, and human orthologs of regions differentially accessible in SOD1-G93A mouse motor neurons were enriched for ALS genetic risk variants. Together, these findings establish a conserved, genetically linked motor neuron signature in ALS.

## In brief

Longitudinal multi-omics in SOD1-ALS mice identifies a disease-associated motor neuron (DM) state transition driven by transcription factor networks. Features of this state are conserved in human ALS and linked to genetic risk, pointing to shared aspects of motor neuron degeneration.

## Graphical Abstract

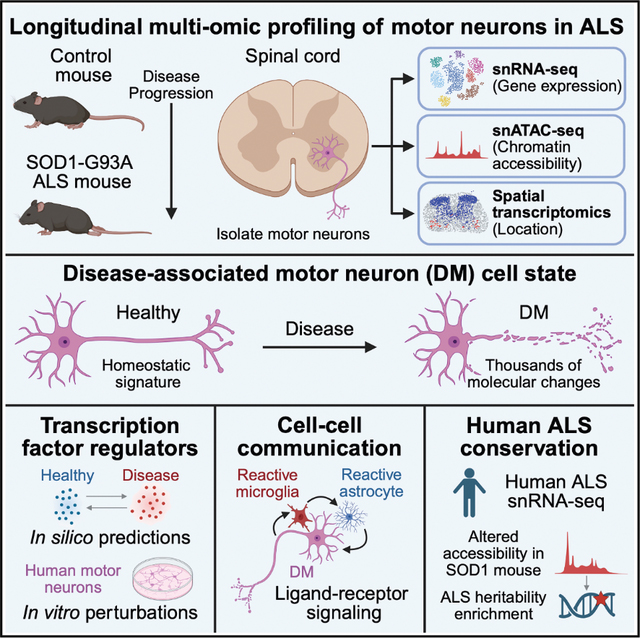

## INTRODUCTION

Amyotrophic lateral sclerosis (ALS) is a progressive disorder distinguished by degeneration of motor neurons in the brain and spinal cord. Motor neuron loss impairs mobility, dexterity, speech, and swallowing and ultimately causes fatal paralysis. ALS is a cruelly rapid disease. Most patients survive just 3 to 5 years after diagnosis.^1^ Effective therapies depend on defining the cellular and molecular drivers of motor neuron degeneration. The first ALS gene identified was *SOD1*, which encodes the antioxidant enzyme superoxide dismutase 1 (SOD1).^2^ *SOD1* mutations cause ~20% of inherited ALS cases, constituting ~2% of total cases.^2–4^ In these cases and in mouse models of the disease, mutant SOD1 causes disease via a toxic gain of function that is incompletely understood.^5–11^

Expression of the ALS-associated variant SOD1-G93A (glycine-to-alanine substitution at residue 93) in mice recapitulates key features observed in ALS patients, providing a useful model.^7^ In both SOD1-G93A mice and ALS patients, not all spinal motor neurons are equally susceptible to degeneration—alpha motor neurons are vulnerable, while gamma and visceral motor neurons are relatively resilient.^12–14^ Even among alpha motor neurons, the larger, fast-firing (FF) ones that innervate fast-twitch muscle fibers are most vulnerable to degeneration.^15–19^ Interestingly, this selective motor neuron vulnerability occurs despite ubiquitous *SOD1* expression.^7,20,21^

To provide insight into motor neuron subtype-selective vulnerability and degeneration, we performed longitudinal single-nucleus RNA sequencing (snRNA-seq), single-nucleus assay for transposase-accessible chromatin using sequencing (snATAC-seq), and spatial transcriptomics to generate comprehensive transcriptomic and epigenomic profiles of vulnerable and resistant spinal motor neurons in the SOD1-G93A mouse. We discovered a population of alpha motor neurons that exhibited an extensive disease-associated gene expression and chromatin accessibility signature, which we have named “disease-associated motor neurons,” or DMs. We used the ATAC and RNA data to nominate transcription factor (TF) drivers of the DM state transition and mediators of motor neuron subtype-selective vulnerability. We performed functional studies in human induced pluripotent stem cell (iPSC)-derived motor neurons and found that DM-associated TFs induce core features of the disease-associated transcriptional program.

Extending these findings to human ALS, we analyzed a large-scale spinal cord snRNA-seq dataset from familial and sporadic ALS patients and control subjects and demonstrated conservation of the DM signature in human alpha motor neurons. Finally, we show that human orthologs of regions with differential accessibility in SOD1-G93A alpha motor neurons are enriched for ALS-associated genetic variants, linking this disease-associated state to human genetic risk.

## RESULTS

### snRNA-seq and spatial transcriptomics of the SOD1-G93A mouse spinal cord

**Figure 1.**
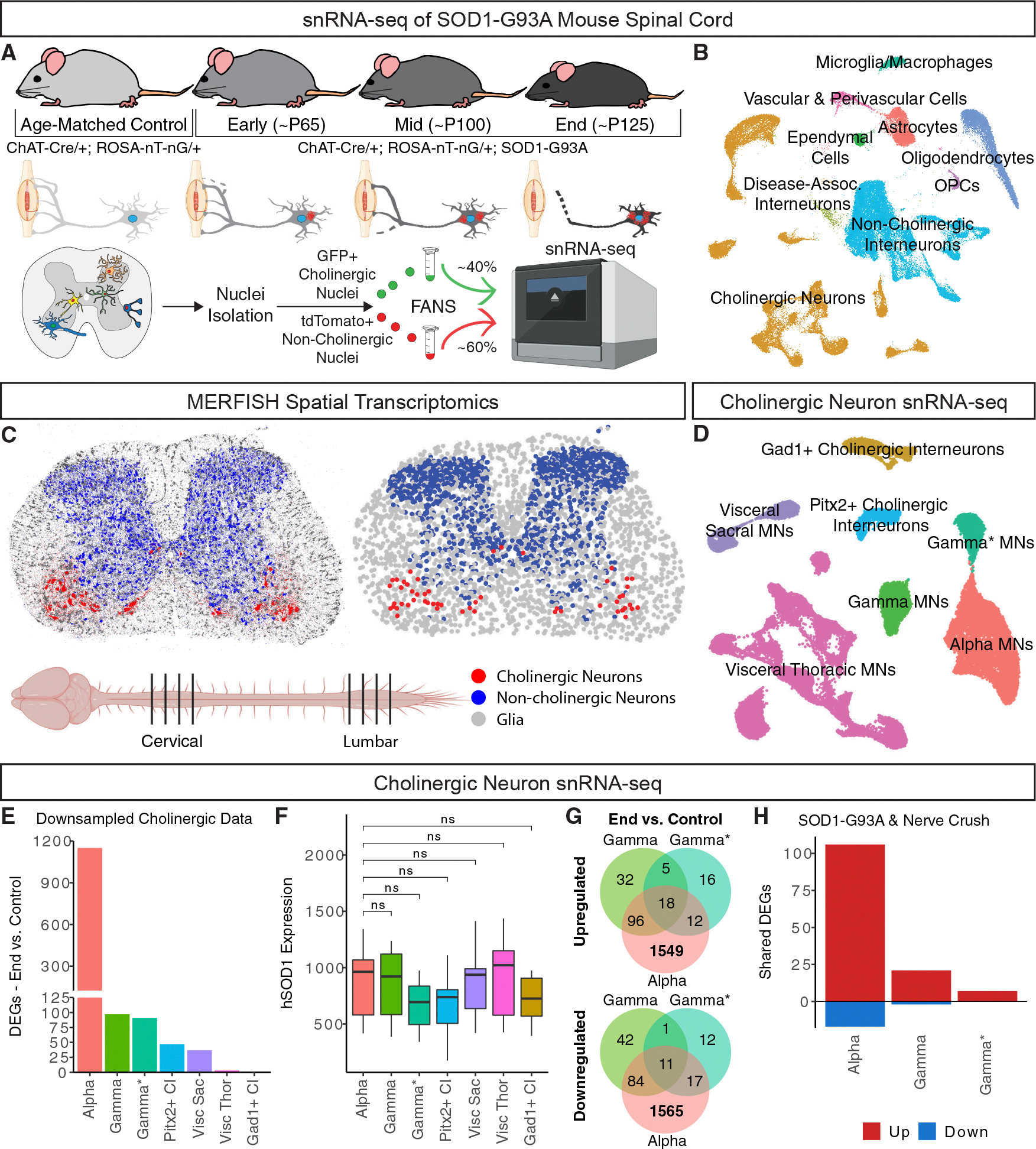
Single-cell transcriptional profiling of the SOD1-G93A mouse spinal cord (A) snRNA-seq of spinal cords from early, mid, and end-stage SOD1-G93A mice and age-matched controls. (B) UMAP of all nuclei. (C) MERFISH spatial transcriptomics of cervical and lumbar spinal cord from SOD1-G93A and control mice. Representative control lumbar sections with transcripts (left) or segmented cells (right) colored by cholinergic neurons, non-cholinergic interneurons, and glia. (D) UMAP of cholinergic neurons. (E) Number of DEGs between end-stage SOD1-G93A and control nuclei for each cholinergic neuron type (DESeq2 Wald test; *p*adj < 0.01). (F) Average hSOD1-G93A transgene expression (counts per million) across cholinergic neuron subtypes (*n* = 11–15 experiments; Welch’s *t* tests with FDR correction; n.s., not significant). (G) Overlap of upregulated (top) or downregulated (bottom) genes in alpha, gamma, and gamma* motor neurons (end-stage versus control; DESeq2 Wald test; *p*adj < 0.01). (H) Shared DEGs from SOD1-G93A motor neurons (from G) and spinal cholinergic neurons after sciatic nerve crush (Shadrach et al.^25^).

To investigate the mechanisms by which motor neurons degenerate and provide insight into motor neuron selective vulnerability in ALS, we performed snRNA-seq of the SOD1-G93A mouse spinal cord. Motor neurons are rare in the spinal cord,^22,23^ so we enriched for cholinergic nuclei (including all motor neurons and some interneuron subtypes^24^) and also captured non-cholinergic nuclei (Figures 1A and S1A). We collected time points from early-stage (~postnatal day 65 [P65]), mid-stage (~P100), end-stage (~P125), and age-matched, non-transgenic control mice (Figure 1A). In total, we transcriptionally profiled >115,000 high-quality nuclei from these four conditions (Figures 1B and S1B).

We clustered nuclei and annotated them using expression of known marker genes^24,26^ (Figures 1B, S1B, and S1C), resulting in ~39% of nuclei coming from cholinergic neurons, including motor neurons, and the rest coming from other spinal cord cell types. To validate our sequencing results and provide spatial context to our data, we performed multiplexed error-robust fluorescence *in situ* hybridization (MERFISH)^27^ to simultaneously probe expression of 140 genes in control and SOD1-G93A mouse spinal cords throughout disease progression (Figures 1C and S1D–S1F). Clustering revealed expected major cell classes and spatial locations of cholinergic neurons (Figures 1C and S1D–S1F).

### Gene expression changes in spinal motor neurons during neurodegeneration

To identify gene expression changes with disease in vulnerable and resistant motor neurons, we first subclustered and annotated the cholinergic nuclei from the snRNA-seq and MERFISH data (Figures 1D and S2A–S2H).^24,28^ Using the snRNA-seq data, we then performed pseudobulk differential expression analysis to find differentially expressed genes (DEGs) between control and end-stage disease conditions for all cholinergic neuron types (Figure 1E). Among cholinergic neurons, alpha motor neurons had the greatest number of DEGs, over an order of magnitude more than other types, which all showed a relatively minor transcriptional response to hSOD1-G93A expression (Figure 1E). This pattern was also true for motor neuron subtypes at the mid-stage (Figure S2C), while only the hSOD1-G93A transgene was differentially expressed at the early stage (Figure S2D). The hSOD1-G93A transgene was expressed at similar levels in alpha motor neurons and the other cholinergic neuron types (Figure 1F), suggesting that the large transcriptional response to disease in alpha motor neurons was not due to disproportionate levels of mutant SOD1 RNA.

Next, we compared DEGs at end-stage disease among vulnerable and resistant skeletal motor neurons to identify common and divergent gene expression changes (Figure 1G; Tables S1A–S1C). We first focused on DEGs shared across all skeletal motor neuron subtypes (18 upregulated and 11 downregulated) (Figure 1G; Tables S1A–S1C). Among shared upregulated genes, we found the regeneration-associated TFs *Sox11* and *Stat3*, which are implicated in peripheral axon regeneration *in vivo*,^29–32^ as well as injury-induced genes *Socs3* and *Abca1*^25,32,33^ (Figure 1G; Tables S1A–S1C).

These findings prompted comparison of SOD1-G93A gene expression changes with bulk translatome data from regenerating motor neurons after sciatic nerve crush^25^ to identify candidate pro-regenerative responses. Among upregulated disease genes in resistant motor neurons, ~14% are also upregulated during regeneration after nerve injury (21/151 in gamma and 7/51 in gamma*), and in alpha motor neurons, ~6% of upregulated genes are shared (106/1,675) (Figure 1H; Table S1D). Some of these genes (e.g., *Gal*, *Adcyap1*, *Gap43*/*Basp1*, *Sprr1a*, and *Atf3*) play established roles in peripheral neuron regeneration.^34–38^ By contrast, there was little overlap between downregulated transcripts after nerve injury and those altered in disease for alpha (17/1,677) and gamma (2/138) motor neurons (Figure 1H; Table S1D). These results suggest that all skeletal motor neurons undergo regeneration-associated transcriptional changes in the SOD1-G93A mouse, but these changes are ultimately insufficient to prevent alpha motor neuron degeneration.

### A DM signature in alpha motor neurons

**Figure 2.**
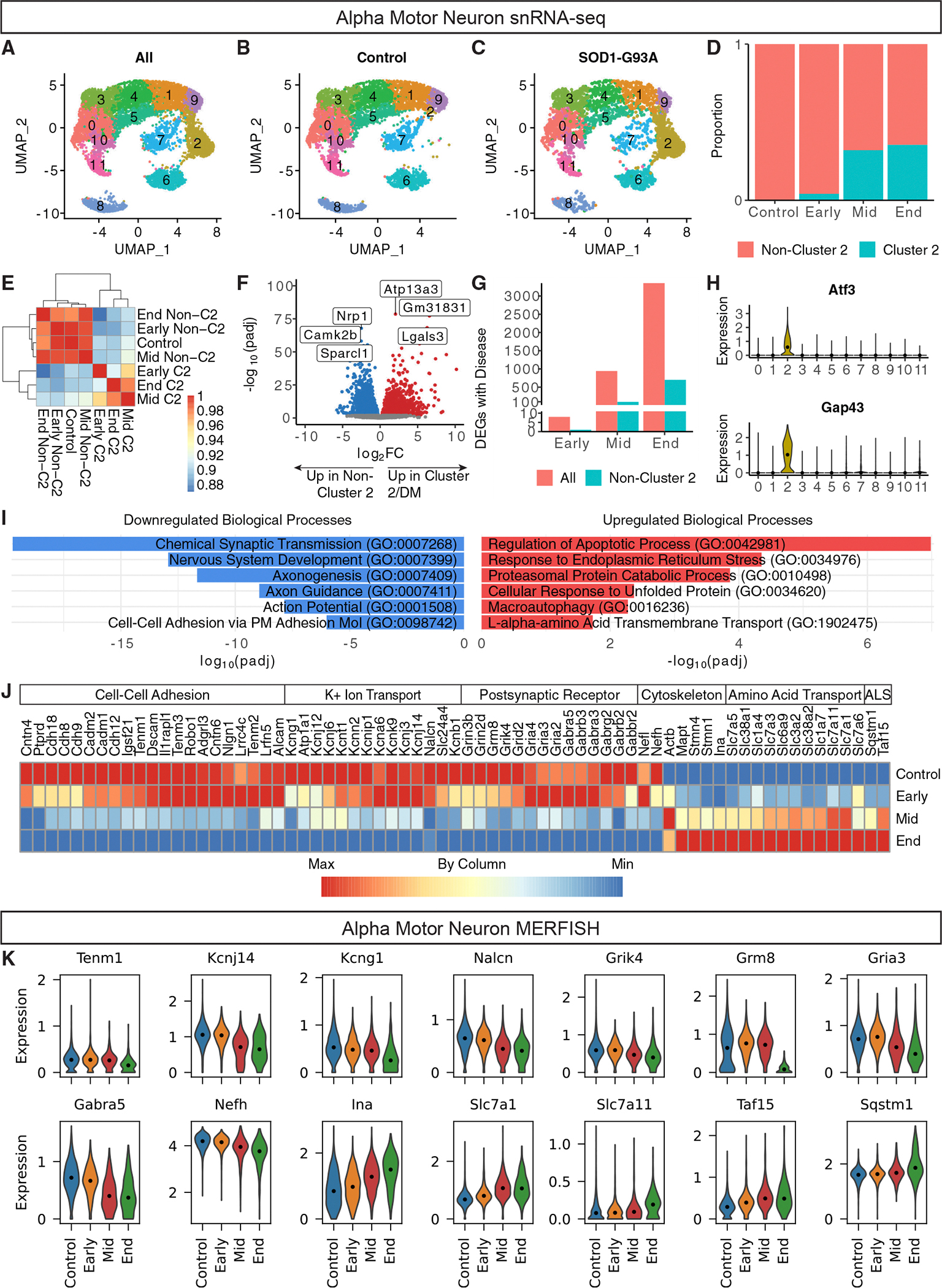
Transcriptional signature of disease-associated alpha motor neurons (A–C) UMAPs of all (A), control (B), and disease (C) alpha motor neurons. (D) Proportion of alpha motor neurons in cluster 2 (DMs) or other clusters. (E) Pearson correlation heatmap using average expression profiles (top 2,000 variable genes) from alpha motor neuron groups (C2 = cluster 2/DM). (F) DEGs in cluster 2/DMs versus other clusters (DESeq2 Wald test; *p*adj < 0.01). (G) Number of DEGs across disease stages versus control with or without the inclusion of cluster 2/DMs (DESeq2 Wald test; *p*adj < 0.01). (H) Log-normalized expression of *Atf3* (top) and *Gap43* (bottom) across clusters. (I) Selected GO biological processes enriched among DM-downregulated (left) and DM-upregulated (right) genes (Enrichr; Fisher’s exact tests with FDR correction). (J) Expression heatmap of selected DEGs across disease progression (median |log_2_FC| for control versus end-stage = 0.91; range 0.39–3.45). (K) Log-normalized expression of select DEGs by MERFISH (two-sided Mann-Whitney U tests between control and end-stage; FDR < 0.01).

To identify changes in alpha motor neuron subtypes with disease, we subclustered the alpha motor neuron transcriptomes into 12 groups (Figure 2A). Strikingly, we observed the emergence of a new transcriptionally distinct alpha motor neuron cluster with disease (cluster 2) (Figures 2B and 2C), which was present at ~4% at the early stage and increased in representation to ~35% at end-stage disease (Figures 2D and S2E). We have named cells in this new cluster disease-associated motor neurons, or DMs. Hierarchical clustering separated DMs from all other SOD1-G93A alphas, which grouped with controls, indicating that many non-DMs remain control-like even at end-stage (Figure 2E). DMs have thousands of DEGs when compared with non-DMs (Figure 2F; Table S1E), supporting classification as a new cell state.

The DM cluster increases in relative abundance with disease (Figure 2D), while clusters 7 and 9 are significantly depleted (Figure S3A). Cluster 7 is marked by *Sema3e* and *Cdh8* (Figure S3B) and may correspond to gluteus and shoulder-innervating alpha motor neurons.^24,39,40^ Cluster 9 is marked by genes associated with FF alpha motor neurons (Figure S3C)^24,41^ and lacks expression of slow-firing (SF) marker genes^24,42^ (Figure S3D). Its reduction in disease is consistent with the preferential vulnerability of FF alpha motor neurons.^15–18^

As the proportion of DMs increased with disease progression, the number of DEGs between disease and control cells also increased (Figure 2G). Notably, we detected very few transcriptional changes at the early disease stage (~P65), suggesting that widespread gene expression alterations emerge later (Figure 2G). We still detected disease-associated DEGs after excluding DMs (Figure 2G), suggesting that some transcriptional changes arise before full entry into the DM state. We refer to cells at mid/end stages that have not yet entered the DM cluster but already exhibit early transcriptional changes as “pre-DMs.”

To identify pre-DM transcriptional changes, we performed differential expression analysis between the control and disease conditions for FF (clusters 9 and 1) and SF (clusters 0 and 10) alpha motor neurons (Figures S3C and S3D). We observed more gene expression changes in FF neurons (213 downregulated and 237 upregulated) than in SF ones (23 downregulated and 37 upregulated), with an overlap of 11 downregulated and 13 upregulated genes (Figure S3E; Tables S1F and S1G). The overlapping upregulated genes include *Sox11*, *Etv4*, and *Atf3* (Figures S3E and S3F), suggesting a response that is at least partially protective. In disease, FF neurons exhibit downregulation of genes involved in potassium ion transport and extracellular matrix organization and upregulation of genes involved in amino acid transport, negative regulation of apoptosis, and integrated stress response signaling (Figures S3G and S3H; Tables S1H and S1I) prior to DM state entry.

DMs show increased expression of known markers of neuronal denervation and axon regrowth, *Atf3* and *Gap43*, and genes involved in apoptosis compared with non-DMs (Figures 2H and 2I; Tables S1E and S1J), suggesting that DMs correspond to denervating, degenerating, and ultimately dying alpha motor neurons. DMs also upregulate genes involved in response to endoplasmic reticulum (ER) stress/unfolded protein, proteasomal protein catabolism, autophagy, and amino acid transport, among other processes, as well as an increase in ALS-associated genes (e.g., *Sqstm1* and *Taf15*) (Figures 2I and 2J; Tables S1E and S1J). Conversely, DMs downregulate genes involved in synaptic transmission (including both pre- and post-synaptic genes), axonogenesis/axon guidance, potassium ion transport/action potential, and cell-cell adhesion (Figures 2I and 2J; Tables S1E and S1K).

To validate the DM transcriptional program *in situ*, we analyzed expression of 106 DM-associated genes in alpha motor neurons from our MERFISH dataset (Figures 2K, S3I, and S3J; Tables S1L and S1M). We confirmed 61 upregulated (~88%) and 32 downregulated (~86%) DM genes identified by snRNA-seq at mid and/or end stages (Figures 2K, S3J, and S3K; Table S1M), demonstrating strong concordance between modalities and indicating that the DM state is not an artifact of tissue dissociation.

Together, these data indicate that alpha motor neurons do not enter the DM state simultaneously. Even at later stages of disease, there are SOD1-G93A alpha motor neurons that remain transcriptionally closer to controls than to DMs (Figures 2A–2E). These findings mirror human ALS, where relatively unaffected motor pools coexist with affected ones, and provide a gene-expression-based view of disease onset at the cellular level.^43^ To define the upstream regulatory mechanisms underlying this transition, we next examined chromatin accessibility dynamics associated with entry into the DM state.

### Paired snATAC-seq and snRNA-seq of spinal motor neurons during neurodegeneration

**Figure 3.**
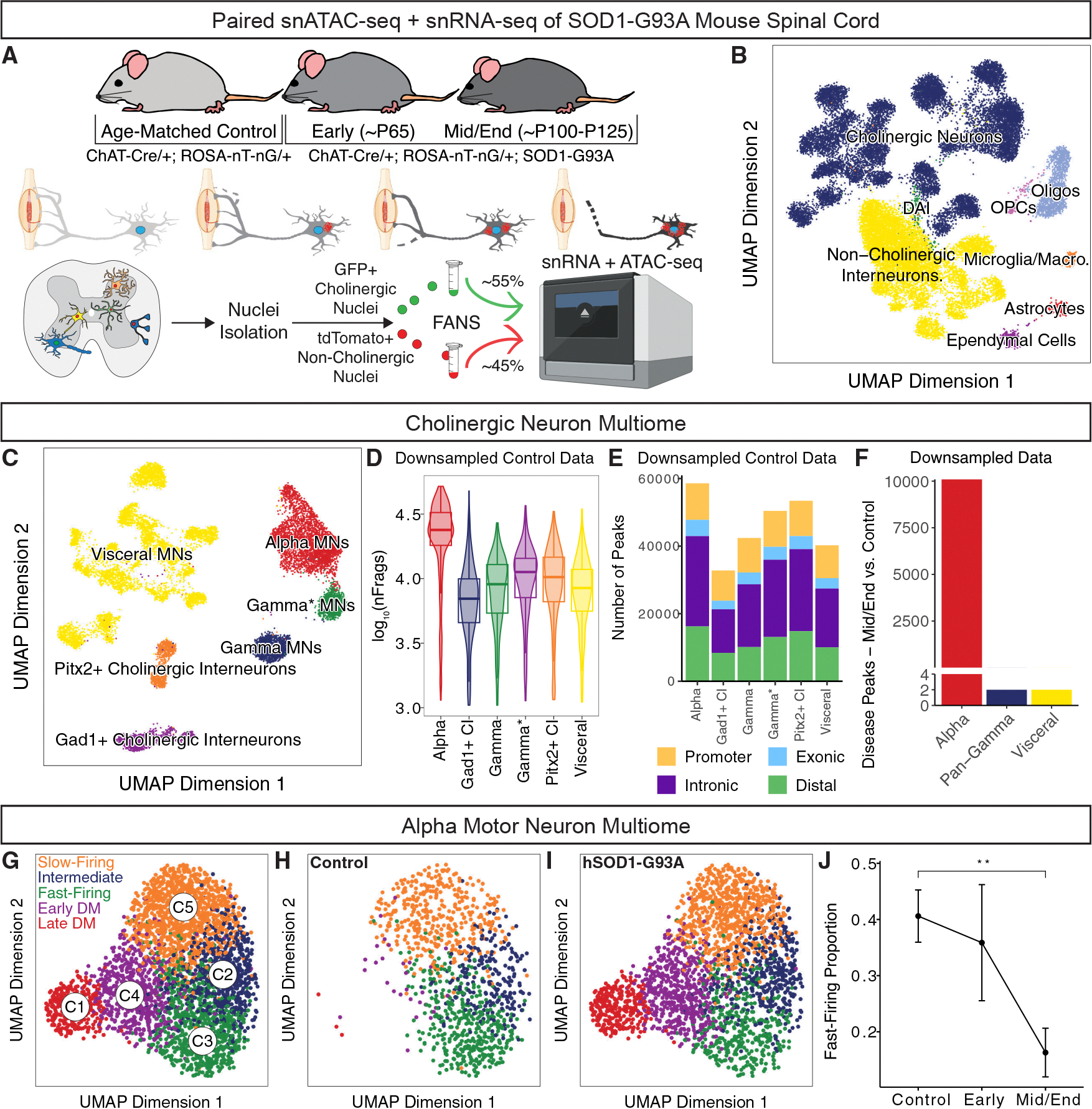
Chromatin accessibility landscapes of spinal cord cell types in the SOD1-G93A mouse (A) Paired snATAC-seq and snRNA-seq of spinal cords from early and mid/end-stage SOD1-G93A mice and age-matched controls. (B) UMAP of all nuclei from integrated paired snRNA-seq and snATAC-seq data. (C) UMAP of cholinergic neurons from integrated paired snRNA-seq and snATAC-seq data. (D) snATAC-seq fragment counts (nFrags) across control cholinergic neuron types. (E) Number of snATAC-seq peaks across genomic annotations in control cholinergic neuron types. (F) Number of differentially accessible peaks between mid/end-stage and control nuclei for motor neuron subtypes (Wilcoxon tests; FDR ≤ 0.1). (G–I) UMAPs of all (G), control (H), and disease (I) alpha motor neurons from snATAC-seq data, with annotations in (G). (J) Proportion of FF alpha motor neurons across conditions (FF/total non-DM; group means ± standard error; *n* = 2–3 experiments per group; one-sided Welch’s *t* test; ** *p* ≤ 0.01).

To capture epigenomic changes during ALS, we profiled chromatin accessibility and gene expression using paired snATAC-seq and snRNA-seq (multiome sequencing) from the mouse SOD1-G93A spinal cord (Figure 3A). We applied a similar strategy as above to sequence motor neurons and other cell types, yielding ~27,000 high-quality multi-omic profiles from early and mid/end-stage SOD1-G93A mice and controls (Figure S4A). Of these, ~54% came from cholinergic neurons, including motor neurons (Figures 3B, S4A, and S4B).

To investigate the chromatin accessibility landscapes of motor neurons, we first subclustered and annotated the cholinergic neuron nuclei (Figures 3C, S4C, and S4D).^24,28^ At baseline in controls, alpha motor neurons showed the highest number of snATAC-seq fragments and the greatest number of peaks among cholinergic neuron types (Figures 3D and 3E), while the fragment size distributions were similar (Figure S4E). By contrast, transcription start site (TSS) enrichment profiles and scores and the fraction of fragments in peaks were lowest in alpha motor neurons (Figures S4F–S4H). These observations are consistent with a long-observed phenomenon that alpha motor neurons are more euchromatic and transcriptionally active than other cell types.^23,44,45^ We next identified chromatin accessibility changes with disease in vulnerable and resistant motor neurons using the control and mid/end-stage disease data (Figure 3F). The alpha motor neuron epigenome was substantially altered with disease progression, with numerous differentially accessible peaks, while resistant pan-gamma (gamma and gamma*) and visceral motor neurons exhibited minimal changes (Figure 3F).

### Chromatin accessibility landscapes of healthy and DM alpha motor neurons

To define the chromatin accessibility landscapes of alpha motor neuron subtypes, we subclustered alpha motor neuron nuclei into five groups using the snATAC-seq data (Figure 3G). As with the RNA data, we found disease-dependent, DM clusters that were essentially absent in the control samples but present in SOD1-G93A samples (Figures 3H and 3I). One DM cluster had less extensive gene expression changes (early DM), while the other had more extensive changes (late DM) and contained alpha motor neurons that were further along in the degeneration/cell death process (Figures 3G and S5A). The remaining three subclusters were well-represented in both control and disease conditions (Figures 3H and 3I). We found that two of these clusters corresponded to FF and SF alpha motor neurons, respectively (Figures 3G and S5A).^24,41,42^ The third cluster showed intermediate expression levels of FF and SF marker genes (Figures 3G and S5A) and may have intermediate electrophysiological properties. These results provide chromatin accessibility landscapes for alpha motor neurons according to DM status (non-DM, early DM, and late DM) and functional subtype (FF, intermediate, and SF).

Consistent with the snRNA-seq data, we observed increasing representation of DMs with disease progression (Figure S5B). To assess concordance between modalities, we compared DM-associated differential expression results from the RNA-only and multiome datasets. We found that 1,050 DM genes were shared across datasets (*p*adj < 0.01), and log_2_ fold-changes were highly correlated (r = 0.87, p ≈ 0), indicating strong reproducibility of the DM signature (Figure S5C).

Because FF alpha motor neurons are preferentially vulnerable in ALS,^15–18^ we tested whether their proportion declined with disease and observed a significant decrease in the proportion of FF alpha neurons with disease (Figure 3J). To further connect RNA- and ATAC-defined states, we performed cross-modal label transfer of alpha motor neuron subtypes from multiome to the RNA-only dataset (Figure S5D). In the RNA-only dataset, disease progression was associated with expansion of cluster 2 (DMs) and depletion of clusters 7 and 9 (Figures 2D and S3A). Following label transfer, we observed stage-dependent depletion of FF neurons and relative enrichment of SF neurons in cluster 7 (Figure S5E). Additionally, among early DM nuclei, the most probable non-DM subtype assignment from label transfer was more frequently FF and less frequently SF compared to the observed subtype composition of non-DM nuclei (Figure S5F), supporting preferential progression of FF alpha motor neurons into the DM state. To assess transcriptional changes with DM progression, we compared early and late DMs and found that late DMs exhibited further upregulation of stress, proteostasis, and apoptotic programs and additional downregulation of synaptic and axonal pathways (Tables S1N–S1P), coinciding with the progressive chromatin accessibility changes observed across DM states.

### TFs associated with the DM state transition

**Figure 4.**
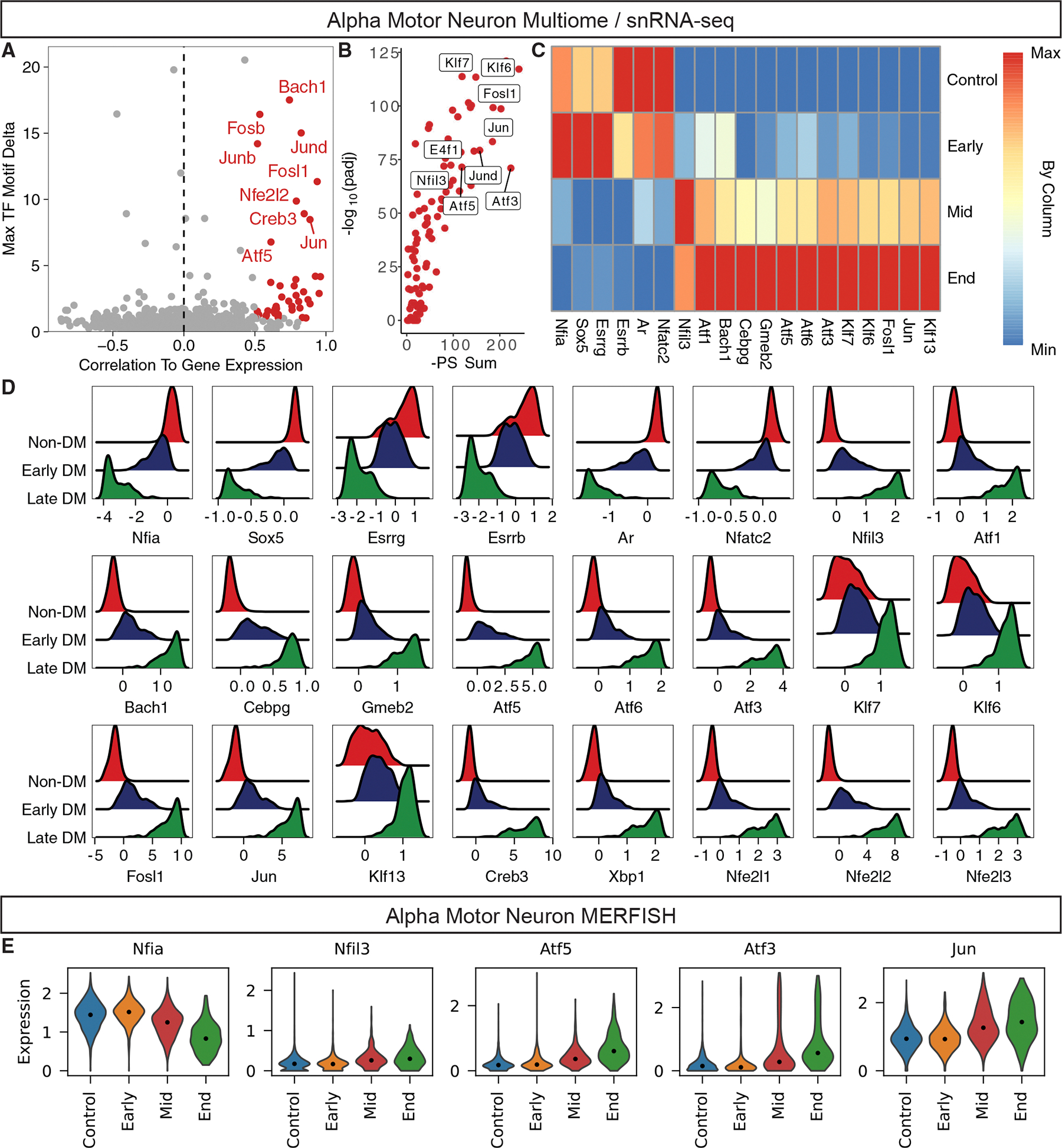
TF regulators of the DM state (A) ArchR identification of positive TF regulators of DM status. TFs in the top quartile of maximum differences in chromVAR deviation *Z* scores with correlation > 0.5 are in red. (B) CellOracle *in silico* knockout results of 106 TFs in the non-DM to DM transition (−PS sum = negative sum of perturbation scores across nuclei). (C) Expression heatmap of selected differentially expressed TFs across disease progression by snRNA-seq (median |log_2_FC| for control versus end-stage = 0.74; range 0.44–6.91). (D) Distributions of chromVAR deviation scores of selected TFs from (A) by DM status. (E) Log-normalized expression of select DEGs by MERFISH (two-sided Mann-Whitney U tests between control and end-stage; FDR < 0.01).

To identify TFs that may drive the transition from healthy alpha motor neurons to DMs, we analyzed the multiome and snRNA-seq data using ArchR^46^ (Figure 4A; Table S1Q) and CellOracle^47^ (Figure 4B; Table S1R). Candidate regulators of the DM transition are TFs whose motif activities correlate with their own gene expression across alpha motor neurons (Figure 4A, *x* axis) and differ across non-DM, early DM, and late DM states (Figure 4A, *y* axis, max TF motif delta). CellOracle simulates TF knockouts to predict changes in cell state trajectories, quantified for each cell by the perturbation score (ps) and aggregated across cells as the ps sum (Figure 4B). TFs identified by both methods include C2H2 zinc-finger factors from the Sp/KLF (*Klf6* and *Klf7*) and GLI-Krüppel (*E4f1*) groups as well as bZIP factors from the AP-1 (*Fosl1*, *Jun*, and *Jund*), ATF/CREB (*Atf3* and *Atf5*), and PAR-bZIP (*Nfil3*) groups (Figures 4A and 4B; Tables S1Q and S1R). Many of these TFs, and others, showed disease-associated changes in gene expression and corresponding alterations in motif accessibility (Figures 4C and 4D). To support these findings, we validated the expression changes of several candidate TFs using MERFISH (Figure 4E; Table S1M).

Two of the top DM regulators, *Atf3* and *Nfil3* (Figures 4A–4E), have been reported to confer neuroprotection and delay disease onset when overexpressed in the SOD1-G93A mouse.^48,49^ Additionally, a recent study showed that *CREB3*, another positive regulator of the DM state, carries a gain-of-function variant that boosts CREB3 activity and protects against human ALS (Figures 4A and 4D).^50^ Furthermore, several of the other TFs—*Xbp1*, *Nfe2l1*, *Nfe2l2*, and *Nfe2l3* (Figure 4D)—have known roles in promoting protein folding, protein degradation, and recovery from oxidative stress. Together, these findings suggest that at least a subset of DM-associated TFs are protective in disease.

**Figure 5.**
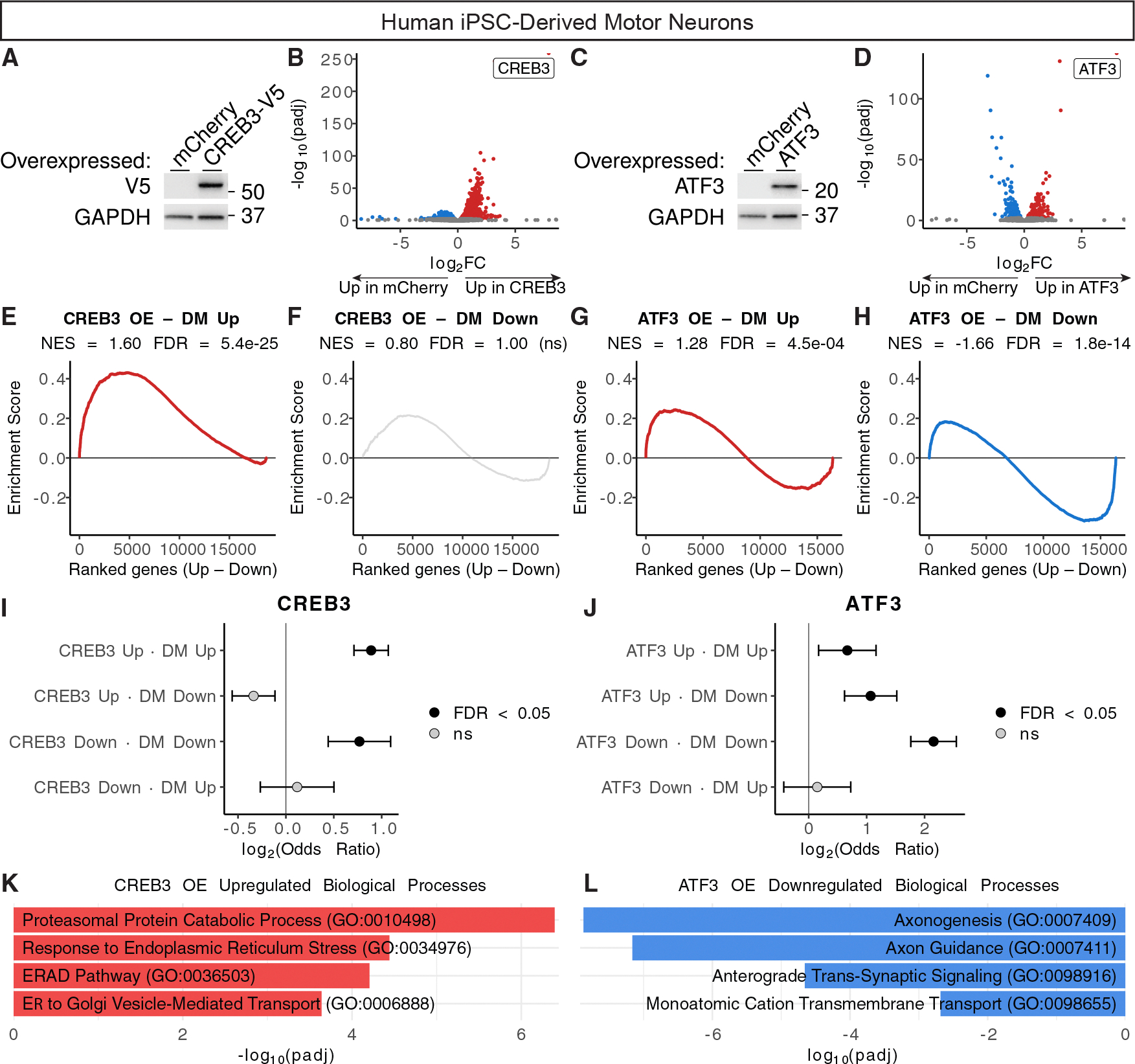
CREB3 and ATF3 induce DM-associated transcriptional programs in human motor neurons (A) Western blot of V5-tagged CREB3 overexpression in human iPSC-derived motor neurons. (B) DEGs in CREB3 overexpression versus mCherry control (DESeq2 Wald test; *p*adj < 0.01). (C) Western blot of ATF3 overexpression in human iPSC-derived motor neurons. (D) DEGs in ATF3 overexpression versus mCherry control (DESeq2 Wald test; *p*adj < 0.01). (E–H) Gene set enrichment analysis (GSEA) of DM-up and DM-down gene sets with genes ranked by the DESeq2 Wald statistic (TF overexpression versus mCherry control; NES, normalized enrichment score): CREB3 DM-up (E), CREB3 DM-down (F), ATF3 DM-up (G), and ATF3 DM-down (H). (I and J) Overlap between TF-induced DEGs and DM gene sets for CREB3 (I) and ATF3 (J) (log_2_ odds ratios with 95% confidence intervals; Fisher’s exact tests with FDR correction across tests). (K and L) Selected GO biological processes enriched among CREB3-upregulated (K) and ATF3-downregulated (L) genes (Enrichr; Fisher’s exact tests with FDR correction). The terms in (K) and (L) are significant when using the leading-edge genes from GSEA as shown or the genes with *p*adj < 0.01 from (B) and (D).

To directly test whether DM-associated TFs can drive features of the DM transcriptional program in human motor neurons, we performed gain-of-function experiments in human iPSC-derived motor neurons targeting two DM-enriched TFs identified in our single-cell analyses, CREB3 and ATF3, with RNA sequencing (RNA-seq) as a readout (Figures 5A–5D; Tables S1S and S1T). Applying gene set enrichment analysis, we found CREB3 expression positively enriched DM-upregulated genes (normalized enrichment score [NES] = 1.60, false discovery rate [FDR] = 5.4 × 10^−25^) (Figures 5E and 5F), whereas ATF3 expression negatively enriched DM-downregulated genes (NES = −1.66, FDR = 1.8 × 10^−14^) (Figures 5G and 5H). These results were corroborated by Fisher’s exact tests (Figures 5I and 5J). CREB3-upregulated and ATF3-downregulated genes were enriched for biological processes that recapitulate key features of the DM state observed *in vivo* (Figures 5K and 5L; Tables S1U–S1X). Quantitatively, CREB3 expression was associated with upregulation of approximately 20% of DM-upregulated genes (precision ~15%), while ATF3 expression was associated with downregulation of approximately 5% of DM-downregulated genes (precision ~34%). Thus, components of the DM transcriptional program represent an active regulatory response to disease pathogenesis, and DM-enriched TFs identified *in silico* can induce key features of the DM state in human motor neurons.

### TFs associated with vulnerable and resistant skeletal motor neurons

We next sought to identify TFs that define selectively vulnerable and resistant motor neuron populations at baseline in the adult spinal cord, building on prior work characterizing TFs in motor neuron development.^51–55^ To do so, we analyzed the control multiome and snRNA-seq data using ArchR^50^ and CellOracle,^47^ as described above, focusing on skeletal motor neuron subtypes (alpha, gamma*, and gamma) and alpha motor neuron subtypes (FF, intermediate, and SF) (Figures S6A–S6G). On top of known motor neuron subtype-specific TFs (e.g., *Esrrg* and *Esrrb*),^24,53,56^ we identified additional TFs linked to specific motor neuron subtypes (Figures S6A–S6G; Tables S1Y–S1AC). Interestingly, we found five TFs (*Esrrg*, *Esrrb*, *Mitf*, *Arntl*, and *Arnt2*) that are important for gene regulatory networks of pan-gamma compared with alpha motor neurons and SF compared with FF alpha motor neurons (Figures S6A–S6G; Tables S1Y–S1AC), providing a set of TFs that are shared among resistant motor neurons. We also identified two shared TFs from the ArchR and CellOracle analyses of alpha motor neuron subtypes, *Maf* and *Arnt2*, which are associated with FF and SF alpha motor neurons, respectively (Figures S6D–S6G; Tables S1AA–S1AC).

To further nominate alpha motor neuron subtype-specific TFs that may be relevant to disease, we looked for those that also had altered expression between early DM and late DM cells. We found that *Arnt2*, *Zfhx3*, and *Arntl* were more active in SF compared with FF alpha motor neurons and had increased expression with DM progression (Tables S1Z and S1N), suggesting a potential protective role in disease, consistent with the relative resistance of SF alpha motor neurons. By contrast, *Maf*, *Mecom*, and *Lhx4* showed the opposite pattern. They were more active in FF compared with SF alpha motor neurons and had decreased expression with DM progression (Tables S1Z and S1N), suggesting a potential deleterious role in disease. Consistent with these expression changes, chromVAR deviation analysis comparing late DM versus early DM alpha motor neurons showed increased accessibility of *Arnt2* and *Arntl* motifs and decreased accessibility of *Lhx4* motifs with DM progression (all FDR < 3 × 10^−8^). Interestingly, Mecom is necessary for the specification of fast motor neurons in zebrafish,^57^ and Maf regulates fast type IIb myofiber determination in mice,^58,59^ raising the possibility that shared transcriptional programs may contribute to fast identity across the motor unit. Overall, we identified TFs associated with vulnerable and resistant motor neurons, which may specify motor neuron subtypes in health and influence disease state (DM versus non-DM) during neurodegeneration.

### Glia become reactive near motor neurons early in disease and then become widespread

**Figure 6.**
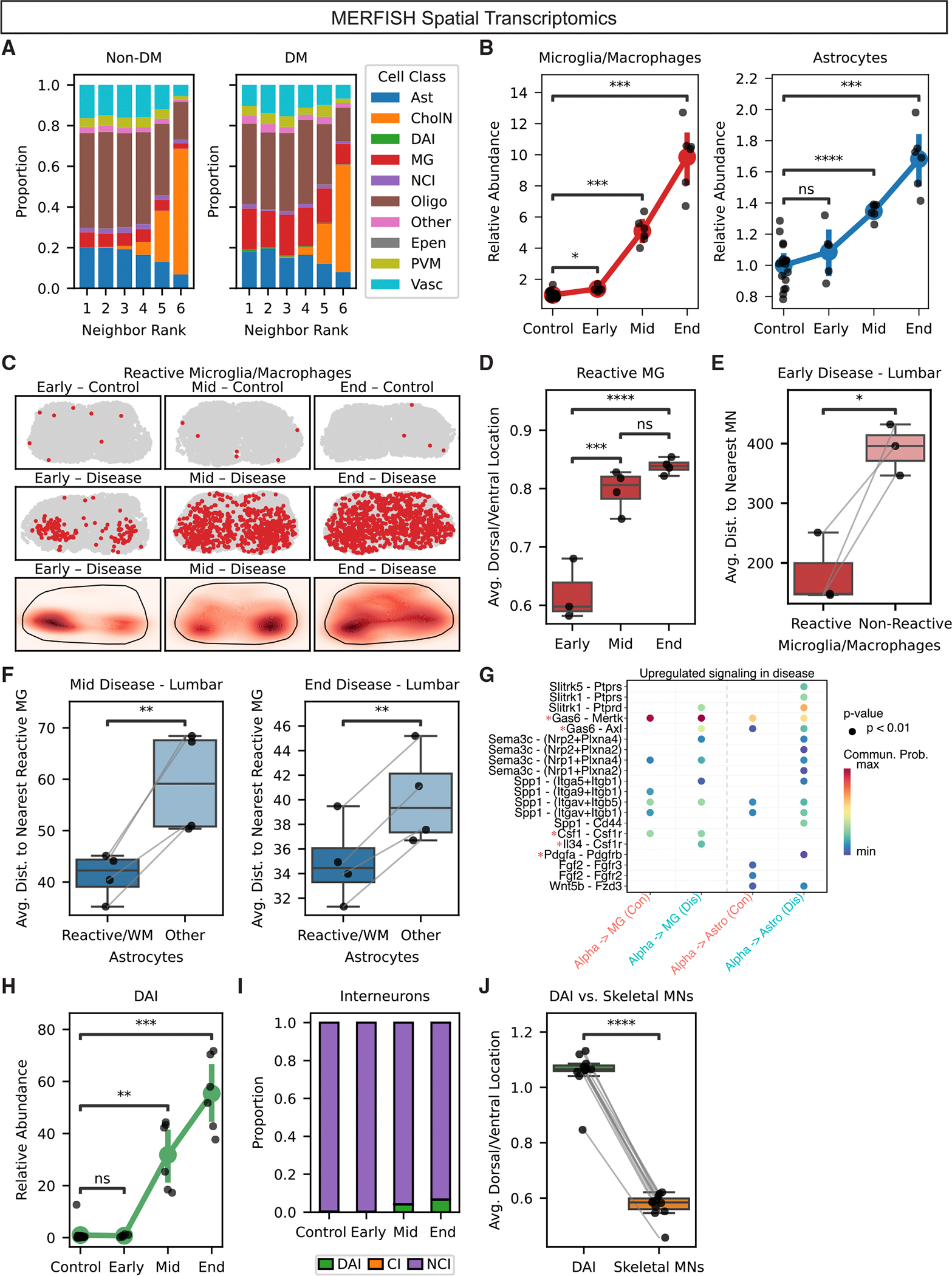
Spatial and compositional dynamics of spinal cord cell types with disease in the SOD1-G93A mouse (A) Cell class composition of the six nearest neighbors to non-DM and DM alpha motor neurons (Ast, astrocytes; CholN, cholinergic neurons; DAI, disease-associated interneurons; MG, microglia/macrophages; NCI, non-cholinergic interneurons; Oligo, oligodendrocytes; Epen, putative ependymal cells; PVM, putative perivascular/meningeal cells; and Vasc, putative vascular cells; see STAR Methods). Microglia/macrophages were significantly enriched at all neighbor positions for DMs compared with non-DMs (Fisher’s exact tests; Bonferroni-adjusted *p* ≤ 0.01). (B) Abundance of microglia/macrophages (left) and astrocytes (right) relative to interneurons. Black points represent per-experiment means (*n* ≥ 5), scaled to control (one-sided Welch’s *t* tests with Bonferroni correction; n.s., not significant; * *p*adj ≤ 0.05, ** *p*adj ≤ 0.01, *** *p*adj ≤ 0.001, and **** *p*adj ≤ 0.0001). Colored points and lines indicate condition means with 95% confidence intervals. (C) Spatial distribution of reactive microglia/macrophages. Top rows: dots represent cells (reactive microglia/macrophages: red, other cells: gray). Bottom row: kernel density plots. (D) Dorsal-ventral position of reactive microglia/macrophages in lumbar spinal cord (lower values = more ventral; higher values = more dorsal). Points represent per-experiment means (*n* ≥ 3; one-way ANOVA followed by Tukey’s honestly significant difference test; n.s., not significant; *** *p* ≤ 0.001 and **** *p* ≤ 0.0001). (E) Distance (μm) from reactive and non-reactive microglia/macrophages to the nearest skeletal motor neuron (see STAR Methods). Points represent per-experiment means (*n* = 3; one-sided paired *t* test; n.s., not significant; ** *p* ≤ 0.01). (F) Distance (μm) from reactive/white matter and other astrocytes to the nearest reactive microglia/macrophage (see STAR Methods). Points represent per-experiment means (*n* = 4; one-sided paired *t* tests; ** *p* ≤ 0.01). (G) Ligand-receptor interactions upregulated in disease with alpha motor neurons as the sender. Bubble size indicates statistical significance (permutation test), and color reflects communication probability (see STAR Methods). Red stars indicate ligand-receptor interactions where the ligand is significantly upregulated in alpha motor neurons at end-stage by snRNA-seq (DESeq2 Wald test; *p*adj < 0.01). (H) Abundance of DAIs relative to other interneurons. Black points represent per-experiment means (*n* ≥ 5), scaled to control (one-sided Welch’s *t* tests with Bonferroni correction; n.s., not significant; ** *p*adj ≤ 0.01 and *** *p*adj ≤ 0.001). Colored points and lines indicate condition means with 95% confidence intervals. (I) Proportions of interneuron subtypes across conditions (DAIs, disease-associated interneurons; CIs, cholinergic interneurons; and NCIs, non-cholinergic interneurons). (J) Dorsal-ventral position of DAI and skeletal motor neurons in the lumbar spinal cord during disease (lower values = more ventral; higher values = more dorsal). Points represent per-experiment means (*n* = 11; one-sided paired *t* test; **** *p* ≤ 0.0001).

In addition to cell-autonomous mechanisms of motor neuron degeneration, non-cell-autonomous mechanisms contribute to ALS.^60–64^ Toxic properties of glial cells collaborate to drive disease progression,^62^ but it remains unclear if these changes arise independently or in response to motor neuron-derived signals. Using spatial transcriptomics, we identified cell types nearest to skeletal motor neurons and observed a marked increase in microglia/macrophages near DMs compared with non-DMs (Figure 6A; Table S1AD), as well as near all skeletal motor neuron subtypes in disease versus control (Figure S7A; Table S1AE). These spatial changes prompted analysis of cell-type abundance with disease relative to interneurons. Microglia/macrophages increased ~10-fold in abundance with disease, echoing previous observations,^65^ along with more modest increases in astrocytes and oligodendrocytes (Figures 6B and S7B). These changes coincide with a significant decrease in abundance of alpha motor neurons but not gamma or gamma* motor neurons with disease (Figure S7B). Importantly, the large increase in microglia/macrophages was not attributable to pre-processing bias (Figure S7C), supporting a genuine rise with disease.

To further assess glial changes, we analyzed our snRNA-seq data and found upregulation of reactive astrocyte (Table S1AF) and disease-associated microglia genes (Table S1AG), along with downregulation of homeostatic microglia genes (Table S1AG).^66–68^ In the MERFISH data, we used *Apoe* and *Gfap* expression to classify microglia and astrocytes as reactive/white matter or non-reactive. Consistent with prior work, we found that glial reactivity increases with disease progression,^69–71^ accompanied by an expansion of DMs and a preferential loss of FF alpha motor neurons (Figures S7D–S7H).

Strikingly, we found that reactive microglia/macrophages were spatially located near motor neurons early in disease and became more widespread with disease progression (Figures 6C–6E, S8A, and S8B).^70,72^ Reactive/white matter astrocytes were also significantly closer to reactive microglia/macrophages than other astrocytes were at mid and end-stage disease (Figures 6F and S8A), consistent with the known role of reactive microglia in activating astrocytes.^64,67^ The MERFISH-based segmentations revealed progressive reductions in alpha motor neuron soma volume and anisotropy, along with increases in solidity and perimeter-to-area ratio with disease (Figures S9A–S9G). However, within a given stage, DM and non-DM motor neurons did not differ (Figures S9H–S9S), indicating that the DM state is primarily molecular rather than morphometric.

Because reactive and proliferating microglia emerged near motor neurons before widespread alpha motor neuron death (Figures 6B–6E and S7B), we posited that there may be a motor neuron-derived signal that activates local microglia. To prioritize candidate pathways, we queried the ChatGPT o3 model to identify ligands that are upregulated in DMs and promote microglial reactivity or proliferation and identified *Csf1*, *Il34*, and *Lgals3* (Table S1E). CellChat^73^ analysis further predicted baseline CSF1-CSF1R signaling from alpha motor neurons to microglia/macrophages and, in disease, increased CSF1-CSF1R signaling to microglia/macrophages from alpha motor neurons, astrocytes, and microglia/macrophages themselves (Figures 6G, S10A, and S10B). It also predicted IL-34-CSF1R signaling specifically from alpha motor neurons to microglia/macrophages in disease, along with additional disease-associated ligand-receptor signaling events (Figures 6G and S10A–S10F). Prior studies show that CSF1R activation drives microglial proliferation^74–76^ and that damaged sensory^77,78^ and motor neurons^78,79^ upregulate CSF1, which directly induces microglial activation. Together, our results show that *Il34* and *Csf1* are both induced in DMs, coincident with increased microglial proliferation and reactivity (Figures 6B, 6C, and 6G; Table S1E).

The changes in reactive glia coincided with increased disease-associated interneurons (DAIs) at mid and end stages (Figure 6H). DAI were also observed in our snRNA-seq and multiome sequencing data (Figures 1B and 3B), consistent with reported interneuron dysfunction in this model.^80–82^ DAI increased to ~7% of interneurons at end-stage (Figure 6I), while ~56% of alpha motor neurons were DMs (Figure S7F). Gene expression changes were significantly correlated between DAI (Table S1AH) and DM populations (r = 0.55, *p* = 6.9 × 10^−280^) (Figures S10G and S10H), and odds ratios for Gene Ontology (GO) biological process enrichment were also significantly correlated (r = 0.76, *p* = 6.6 × 10^−67^) (Figure S10I; Tables S1AI and S1AJ), demonstrating convergence on shared stress, proteostasis, and apoptotic programs alongside suppression of synaptic and axonal pathways (Figure S10J). DAIs were located more dorsally in the spinal cord than motor neurons (Figure 6J) and may contribute to or result from the increase in reactive glia in more dorsal locations with disease progression. Future work is required to elucidate the interactions among reactive glia, DMs, and DAI, but these findings raise the possibility that early glial reactivity may be influenced by signals from nearby motor neurons.

### DM signature in human ALS

**Figure 7.**
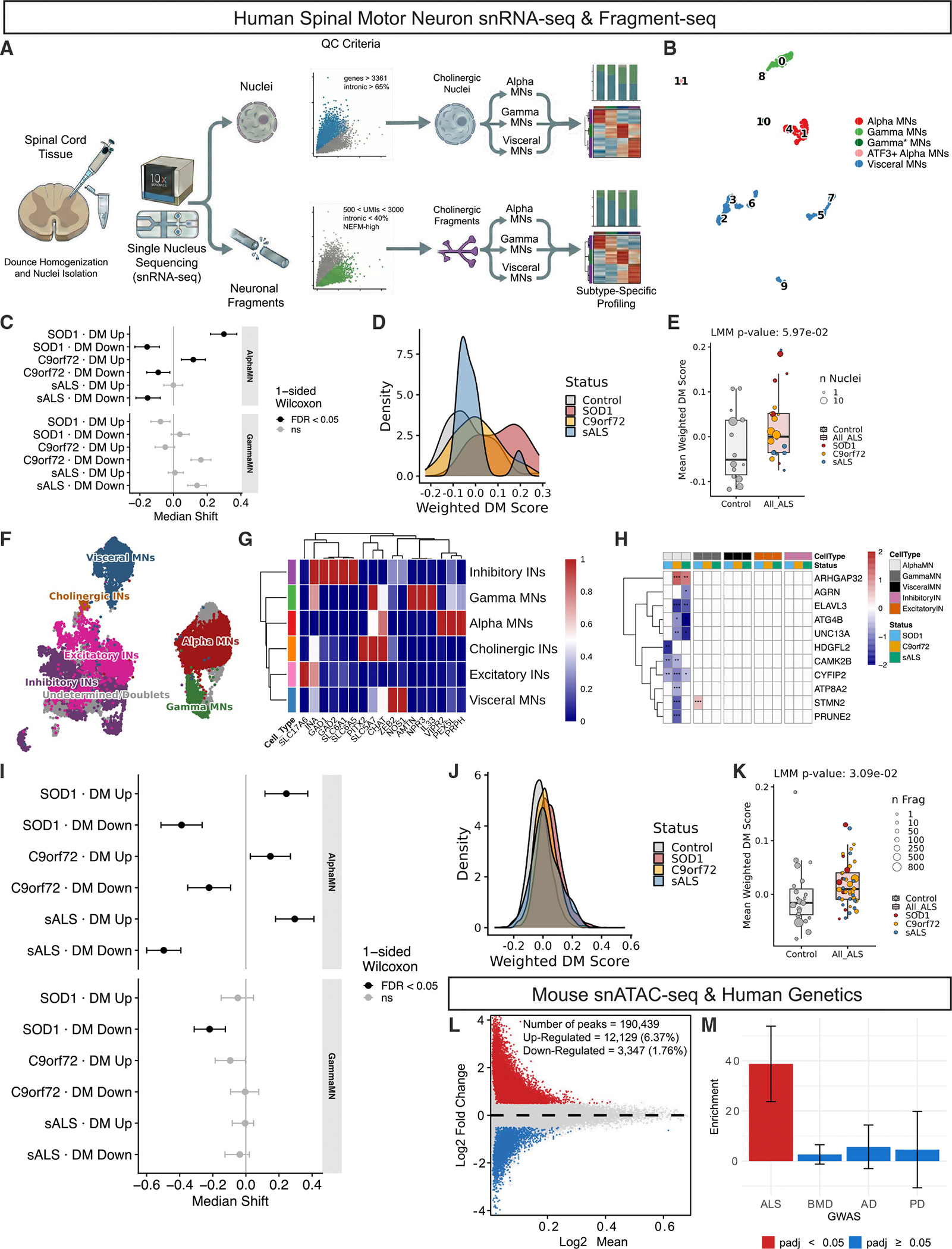
Single-nucleus and fragment transcriptomic profiling of human spinal motor neurons and genetic links to ALS (A) snRNA-seq experimental and analysis workflow for human spinal cord samples. (B) UMAP of human motor neuron snRNA-seq nuclei. (C) Wilcoxon rank-based gene set enrichment of DM-up and DM-down gene sets across ALS subtypes versus control in alpha and gamma motor neuron nuclei. Points indicate median shifts in NEBULA (negative binomial mixed model using a large-sample approximation)-derived Wald/z statistics for each gene set relative to all other genes tested (Hodges-Lehmann estimates with 95% confidence intervals). Significance was assessed by one-sided Wilcoxon-Mann-Whitney rank-sum tests with FDR correction across tested comparisons. (D) Distribution of DM scores for control and ALS alpha motor neuron nuclei. (E) DM scores from alpha motor neuron nuclei across control and pan-ALS conditions. Points represent sample/donor means. Significance was assessed using linear mixed models fit to individual nuclei with sample identity as a random effect (see STAR Methods). (F) UMAP of neuronal fragment profiles. (G) Expression heatmap of selected marker genes for fragment clusters (values min-max normalized by column). (H) Expression heatmap of TDP-43 cryptic splicing target genes differentially expressed in any neuronal subtype (NEBULA differential expression analysis of ALS subtypes versus control; FDR correction across TDP-43 cryptic splicing target genes [see STAR Methods]; *padj < 0.1, **padj < 0.05, and ***padj < 0.01; values *z*-score normalized by row). (I) Wilcoxon rank-based gene set enrichment of DM-up and DM-down gene sets across ALS subtypes versus control in alphaFRAGs and gammaFRAGs. Points indicate median shifts in NEBULA-derived Wald/z statistics for each gene set relative to all other genes tested (Hodges-Lehmann estimates with 95% confidence intervals). Significance was assessed by one-sided Wilcoxon-Mann-Whitney rank-sum tests with FDR correction across tested comparisons. (J) Distribution of DM scores for control and ALS subtype alphaFRAGs. (K) DM scores from alphaFRAGs across control and pan-ALS conditions. Points represent sample/donor means. Significance was assessed using linear mixed models fit to individual fragments with sample identity as a random effect (see STAR Methods). (L) Differentially accessible peaks in alpha motor neurons (mid/end-stage versus control; Wilcoxon test; FDR ≤ 0.05, |log_2_FC| ≥ 0.5). (M) Heritability enrichment from partitioned LD score regression using peaks from (L) across ALS, BMD (bone mineral density abnormality), AD (Alzheimer’s disease), and PD (Parkinson’s disease). Bars show enrichment estimates ± standard error and statistical significance (Z-tests on regression coefficients with FDR correction across tested traits).

To determine whether the DM transcriptional program identified in the SOD1-G93A mouse is conserved in human ALS, we analyzed human spinal cord snRNA-seq data from 58 individuals with ALS (12 *SOD1*-associated ALS [SOD1-ALS], 25 *C9orf72*-associated ALS [C9-ALS], and 21 sporadic ALS [sALS]) and 27 non-neurological controls (Figure 7A).^83^ We identified 648 motor neurons that subclustered into alpha, gamma, putative gamma*, and visceral motor neuron populations based on known markers (Figures 7B, S11A, and S11B). Cluster 10 displayed limited expression of gamma (*AMTN* and *IL33*) and alpha (*PEX5L*, *STK32A*, *TPD52L1*, and *VIPR2*) markers but was enriched for gamma* markers (*GPR149*, *RORA*, and *ESRRG*) (Figures S11A and S11B). It was therefore classified as putative gamma* motor neurons (Figure 7B). Cluster 11 most resembled alpha motor neurons by marker expression and showed elevated *ATF3* expression, consistent with a potential DM-like state, but comprised only 18 nuclei (Figures 7B, S11A, and S11B). Due to its small size, we conservatively excluded cluster 11 from subsequent analyses.

We performed differential expression analysis (see STAR Methods) comparing disease cases to controls (Tables S1AK–S1AM). We defined cross-species DM gene signatures by mapping mouse DM genes (*p*adj < 0.01) to human orthologs, filtering for detectability in human alpha motor neuron nuclei, and selecting the top 1,000 DM-up and top 1,000 DM-down genes by effect size. Using Wilcoxon rank-based gene set enrichment analysis, we found the DM-up signature was significantly enriched in SOD1-ALS and C9-ALS alpha motor neuron nuclei, and the DM-down signature was significantly depleted in SOD1-ALS, C9-ALS, and sALS alpha motor neuron nuclei (Figure 7C). No significant enrichment or depletion of these signatures in the expected direction was observed in resistant gamma motor neurons (Figure 7C), consistent with subtype specificity of the DM signature.

SOD1-ALS alpha motor neuron nuclei showed a clear increase in weighted DM score (see STAR Methods) at the single-nucleus level (Figure 7D). Using models that accounted for sample identity, we found a statistically significant increase in average DM score in pan-ALS versus control (Figure 7E) and in SOD1 versus control (Figure S11C). By contrast, gamma and visceral motor neuron nuclei showed no such differences (Figures S11D and S11E). Together, these results support conservation of the DM signature in human ALS.

Of the 773,198 nuclei sequenced, only 150 bona fide alpha motor neurons (excluding cluster 11) passed quality control across all conditions, underscoring their rarity in the human spinal cord.^22,23^ To address this limitation, we leveraged an additional transcriptomic population within the same dataset—neuronal fragments—previously described in human spinal cord single-nucleus data.^22^ These fragments are characterized by low gene and transcript counts, high relative expression of neurofilament genes, a low proportion of unspliced, intron-containing reads, and depletion of nuclear-localized lncRNAs. Neuronal fragments contain sufficient mRNA to be subclustered by neurotransmitter and lineage marker genes.^22^ Although not directly comparable to nuclei, fragment-to-fragment comparisons could capture biologically meaningful disease-associated differences within rare cell types, such as alpha motor neurons (Figure 7A).

We identified 23,192 neuronal fragments, which clustered by neurotransmitter identity, and further resolved alpha, gamma, and visceral motor neuron fragments (Figures 7F, 7G, and S12A–S12C). We compared alpha motor neuron fragments (alphaFRAGs) from C9 and sALS patients to controls and found a transcriptional signature consistent with TAR DNA-binding protein 43 (TDP-43) pathology (Tables S1AN and S1AO). Several TDP-43 cryptic splicing targets were downregulated in both C9 and sALS alphaFRAGs, including *UNC13A*, *ATG4B*, *ELAVL3*, and *CYFIP2*, while *STMN2*, *PRUNE2*, and *ATP8A2* were significantly reduced in C9-ALS (Figure 7H).^84–86^ This pattern was less evident in SOD1 alphaFRAGs and absent in more resistant motor neuron subtypes (Figure 7H; Table S1AP). Thus, alphaFRAGs capture biologically relevant disease-associated transcriptional changes.

DM-up and DM-down gene sets showed significant enrichment and depletion, respectively, in alphaFRAGs across ALS subtypes (Figure 7I). Applying the weighted DM score, we observed increased scores in ALS alphaFRAGs compared with controls (Figure 7J). Using models that accounted for sample identity, we found a significant increase in the average DM score in pan-ALS alphaFRAGs (Figure 7K). The DM signature was also present in a previously published dataset, in which skeletal motor neuron transcriptomes were collected by laser capture microdissection from sALS postmortem tissue (Figure S12D).^87^ Neuronal fragments derived from less affected motor neuron subtypes (gamma, visceral) showed no significant differences in DM scores between ALS cases and controls (Figures S12E and S12F). Differential expression analysis of alphaFRAGs further revealed ALS-associated transcriptional changes consistent with the DM signature (Figures S12G–S12J; Tables S1AN–S1AQ). Together, these orthogonal analyses demonstrate conservation of the DM signature in human ALS alpha motor neurons.

### Human ALS genetic risk converges with disease-associated chromatin changes in SOD1-G93A motor neurons

We next asked whether the regulatory pathways underlying the DM state are also connected to human ALS genetic risk. To test this, we integrated disease-associated differentially accessible regions from alpha motor neurons (Figure 7L) with human genome-wide association studies (GWASs). Approximately 40% of these differentially accessible regions could be confidently lifted over from the mouse to the human genome, and these orthologous regions were analyzed using partitioned linkage disequilibrium (LD) score regression to assess enrichment for ALS heritability.^88,89^

Our results demonstrate significant heritability enrichment using GWAS summary statistics from human ALS^90^ (Figure 7M), but no such enrichments using those from a non-brain-related trait (bone mineral density abnormality [BMD]) or from other neurodegenerative disorders (Alzheimer’s disease [AD] and Parkinson’s disease [PD]) (Figure 7M).^91–93^ The ALS GWAS has fewer samples than the other studies do (152,268 for ALS, 426,824 for BMD, 788,989 for AD, and 2,525,897 for PD),^90–93^ suggesting that this result is not driven by GWAS sample size differences. We performed additional partitioned heritability analyses using differentially accessible regions defined in the control condition (Figures S13A–S13D). None of these comparisons showed significant enrichment for ALS GWAS heritability (or for AD, PD, or BMD) after FDR correction, suggesting that baseline alpha motor neuron subtype-specific chromatin accessibility may not account for the ALS heritability enrichment observed in disease.

To identify genes mediating the ALS heritability enrichment within disease-associated differentially accessible regions from alpha motor neurons, we performed Hi-C coupled Multi-marker Analysis of GenoMic Annotation (H-MAGMA) with the most recent ALS GWAS^90^ and intersected the output with genes located in the differentially accessible regions (Figure S13E; Table S1AR).^94,95^ A handful of the prioritized genes are significantly differentially expressed with disease in SOD1-G93A mouse alpha motor neurons and may contribute to disease pathogenesis (Figure S13F). Collectively, our results suggest that despite different upstream triggers of degeneration (mutant SOD1 transgene in mice versus various genetic and pathological triggers in humans), aspects of the genetic basis of motor neuron degeneration are shared between the SOD1-G93A mouse model and human ALS.

## DISCUSSION

By employing longitudinal single-nucleus RNA/ATAC-seq and spatial transcriptomics throughout disease progression in the SOD1-G93A mouse model of ALS, we uncovered extensive molecular changes in a subset of vulnerable alpha motor neurons. Alpha motor neurons with these gene expression and chromatin accessibility changes have transitioned from healthy cells into a distinct cell state, which we have named DMs.

Individual alpha motor neurons do not transition to the DM state all at once—we observed a small number of DMs before overt behavioral phenotypes were apparent as well as many non-DM alpha motor neurons that persisted even at later stages of disease. These findings allow us to frame disease onset from a cellular perspective, based on gene expression and functional properties, as well as from an organismal level, based on behavior and survival.

The transcriptional changes distinguishing DMs from healthy alpha motor neurons arise, at least in part, from specific TFs that we have nominated based on our ATAC and RNA-seq data. Importantly, our gain-of-function experiments in human iPSC-derived motor neurons demonstrate that CREB3 and ATF3—two DM-associated TFs—can elicit distinct DM-associated transcriptional programs. These findings indicate that key features of the DM state reflect an active and regulated transcriptional response.

In addition to cell-intrinsic programs, we observed a striking increase in reactive microglia/macrophages near motor neurons early in disease, followed by widespread glial reactivity at later stages. This early proximity is notable, given that the mutant SOD1 transgene is expressed in all cells and could have driven glial activation through purely cell-autonomous mechanisms. Instead, this pattern, along with gene expression changes in relevant cell types, suggests that local signals from motor neurons may contribute to early glial activation.

While snRNA-seq from human postmortem tissue represents a late-stage snapshot of degeneration, our analyses detect cross-species conservation of a DM-like molecular signature in human disease. Consistent with this, prior studies show that human orthologs of DM-upregulated *Gap43*, *Ngf*, and *Jun* all show increases in RNA levels or encoded protein products in postmortem sALS spinal cords,^96–98^ while human orthologs of DM-downregulated *Gria2* and *Gria4* show downregulation in motor neurons from sALS and SOD1-ALS cases.^99^

Our finding linking disease-associated regulatory changes observed in the SOD1-G93A model to genetic risk for ALS in humans suggests that the regulatory pathways accompanying the DM state overlap with those contributing to ALS susceptibility. Thus, the SOD1 mouse can be a powerful model to study fundamental aspects of motor neuron degeneration and to develop and test therapies aimed at increasing motor neuron resilience.

Continued exploration of the interplay between genetic factors, transcriptional regulation, and motor neuron subtype-specific responses will be essential for advancing our understanding of this devastating disease and for evaluating whether elements of the DM program can be therapeutically modulated to enhance motor neuron resilience.

### Limitations of the study

The SOD1-G93A mouse models a single genetic form of ALS, whereas human disease arises from diverse etiologies, most associated with TDP-43 pathology. Nonetheless, the convergence between the DM program and human ALS transcriptomic and genetic signals suggests that core features of this state are shared across disease contexts, although additional subtype-specific pathways may also contribute to motor neuron degeneration. Human analyses were limited by the rarity of alpha motor neuron nuclei, reducing statistical power, particularly for sALS and SOD1 cases. Disease heterogeneity, including variable lower motor neuron involvement, further complicates the identification of shared transcriptional programs. Finally, while neuronal fragments introduce some uncertainty regarding their biological origin, their recapitulation of DM enrichment patterns observed in nuclei supports their use as an orthogonal validation, although further work will help define their provenance and broader applicability.

## RESOURCE AVAILABILITY

### Lead contact

Requests for further information and resources should be directed to and will be fulfilled by the lead contact, Aaron D. Gitler (agitler@stanford.edu).

### Materials availability

This study did not generate any new, unique reagents.

### Data and code availability

- Raw and processed sequencing data have been deposited in the NCBI Gene Expression Omnibus (GEO) under accession numbers GEO: GSE306676 for the snRNA-seq data and GEO: GSE306675 for the multiome (paired snATAC/snRNA-seq) data. An interactive web portal for exploring the snRNA-seq dataset is available at http://www.spinalcordatlas.org. The processed MERFISH spatial transcriptomics data are available on Zenodo: https://doi.org/10.5281/zenodo.16938739.
- Original code is available on GitHub: https://github.com/omgautier/Gautier_Blum_2026.
- Any additional information required to reanalyze the data reported in this paper is available from the lead contact upon request.

## STAR★METHODS

### EXPERIMENTAL MODEL AND STUDY PARTICIPANT DETAILS

#### Mouse Strains and Husbandry

All procedures involving mice were performed in accordance with a protocol approved by the Administrative Panel of Laboratory Animal Care of Stanford University (protocol no. 30643). For snRNA-seq and multiome sequencing experiments, ChAT-IRES-Cre mice (B6;129S6-*Chattm2(cre)Lowl*/J; Jax Strain #: 006410) were crossed with ROSA^nT-nG^ mice (B6;129S6-Gt(ROSA)26Sortm1(CAG-tdTomato*,-EGFP*)Ees/J; Jax Strain #: 023035) to generate mice homozygous for both alleles. Resulting female mice were crossed with male hemizygous SOD1-G93A mice (B6SJL-Tg(SOD1*G93A)1Gur/J; Jax Strain #: 002726). Male and female progeny that carried the SOD1-G93A transgene were used for the early-, mid-, and end-stage SOD1 experiments, and progeny that lacked the SOD1-G93A transgene were used for age-matched control experiments. For MERFISH experiments, male SOD1-G93A mice (B6SJL-Tg(SOD1*G93A)1Gur/J; Jax Strain #: 002726) and age-matched wild-type male mice (B6SJLF1/J; Jax Strain #: 100012) were used. All mice were housed with food and water available *ad libitum* in a 12-h light/dark environment at ambient temperature and humidity. Symptomatic mice carrying the SOD1-G93A transgene were given HydroGel (ClearH2O) and food on the cage floor. All animals were euthanized at or before the humane euthanasia point, which was defined as the inability of a mouse to right itself within 20 s after being placed on its back or side.

#### Human Postmortem Spinal Cord snRNA-seq and Fragment Data

Human postmortem lumbar spinal cord single-nucleus RNA-seq data were obtained from Cao et al. (2025),^83^ who performed snRNA-seq on tissues from individuals with sporadic ALS, *C9orf72*-associated ALS, *SOD1*-associated ALS, and non-neurological controls. We analyzed the motor neuron subset of the published dataset as provided. In addition, we extracted and analyzed fragment data present in the full dataset but not examined in the original study. Tissue procurement and cohort details are described in the original publication. We did not have access to identifiable participant information.

#### Human iPSC Line

Human induced pluripotent stem cell experiments used the WTC11 hNIL line harboring doxycycline-inducible NGN2, ISL1, and LHX3 transgenes. Cells were maintained and differentiated as described in method details.

### METHOD DETAILS

#### Nuclei Isolation and Fluorescence-Activated Nuclei Sorting (FANS)

Nuclei isolation and sorting was adapted from Blum et al. (2021), and buffer recipes are found in Blum et al. (2021).^24^ Twenty-four snRNA-seq experiments and five multiome (paired snATAC/snRNA-seq) experiments were performed. For each experiment, several mice (~3–12) were euthanized with CO2. The spinal cords were rapidly hydraulically extruded using a PBS-filled syringe with a blunt 21 G needle. Two spinal cords were homogenized at a time in a 2 mL Dounce homogenizer (Sigma-Aldrich, D8938–1SET) containing 2 mL of nuclei extraction buffer. The spinal cords were homogenized with ten strokes of pestle A followed by five strokes of pestle B. The homogenate from all cords was transferred to a 50 mL round-bottom centrifugation tube (Thermo Scientific, 3118–0050PK), and 8 mL of nuclei spin buffer 1 was added. Then, 5 mL of nuclei spin buffer 2 was layered gently underneath the homogenate solution. The nuclei were pelleted through the cushion in a swinging-bucket centrifuge (3200×g, 4°C, 15 minutes). The supernatant was rapidly discarded, and the nuclei were resuspended in 5 mL of nuclei spin buffer 1. Next, 5 mL of nuclei buffer 3 was gently layered underneath the nuclei solution. The nuclei were pelleted through the cushion in a swinging-bucket centrifuge (3200×g, 4°C, 15 minutes). The supernatant was rapidly discarded, and the pellet was resuspended in loading buffer containing DAPI.

FANS was performed on a BD Biosciences FACSAria II flow cytometer using the 70 μm nozzle. Single nuclei were gated using DAPI and side scatter measurements to exclude debris and multiplets. Among the single nuclei, EGFP+/tdTomato− and tdTomato+/EGFP− nuclei were identified using a two-dimensional scatterplot. Nuclei were sorted such that ~40% were EGFP+/tdTomato− and ~60% tdTomato+/EGFP− for the snRNA-seq experiments, and ~55% were EGFP+/tdTomato− and ~45% tdTomato+/EGFP− for the multiome sequencing experiments.

#### 10x Genomics snRNA-seq and Multiome Sequencing

Following nuclei sorting, either snRNA-seq (Chromium Single Cell 3′ Reagent Kit v3, 10x Genomics) or multiome ATAC + gene expression profiling (Chromium Single Cell Multiome ATAC + Gene Expression v1, 10x Genomics) was performed according to manufacturer protocols. Libraries were sequenced on an Illumina NextSeq 550 or NovaSeq 6000 using run parameters specified by 10x Genomics. For the final snRNA-seq analysis, control experiments from Blum et al., Nat. Neurosci., 2021 (GEO: GSE161621) were also used.^24^

#### snRNA-seq: Data Pre-Processing and Ambient RNA Removal

Sequencing reads were demultiplexed and aligned to a custom mouse pre-mRNA reference transcriptome containing mutant hSOD1 (10x Genomics) using the cellranger mkfastq and cellranger count pipelines (Cell Ranger v3.1.0, 10x Genomics). Subsequent analyses were performed in R (v4.1.1) using the Seurat package (v4.0.4).^102^ A Seurat object was made for each sample using the filtered count matrix which excludes putative empty droplets. Each Seurat object was processed to obtain clusters (NormalizeData, FindVariableFeatures, ScaleData, RunPCA, FindNeighbors with dims = 1:20, FindClusters with resolution = 0.5), and these clusters were used with SoupX (v1.6.2)^105^ to remove ambient RNA contamination (SoupChannel, setClusters, autoEstCont). The SoupX-adjusted counts were rounded to the nearest integer. Mitochondrial reads were then removed and stage information (“ctl”, “sod.early”, “sod.mid”, “sod.end”) was added to the metadata of each object. Some samples were derived from a mixture of both SOD1-G93A mice of a given stage and control mice. For these samples, each RNA profile was assigned to the appropriate condition using the presence or absence of hSOD1 counts.

#### snRNA-seq: Integration, Quality Control, and Clustering

The Seurat objects were integrated using Seurat’s reference-based integration workflow. First, the Seurat objects were log-normalized (NormalizeData), and variable features were identified for each object individually (FindVariableFeatures with selection.method = “vst” and features = 2000). Features that were repeatedly variable across datasets were selected for integration (SelectIntegrationFeatures). The sample “210727_nova_CZI/sod1_mn_nuclei_2_9”, which was derived from control and early-stage SOD1-G93A mice, was selected as the reference. The reference was used to find integration anchors (FindIntegrationAnchors), and then the integrated object was created (IntegrateData).

Clustering and visualization were performed using the integrated Seurat object (DefaultAssay set to “integrated”, ScaleData, RunPCA, RunUMAP with reduction = “pca” and dims = 1:20, FindNeighbors with reduction = “pca” and dims = 1:20, FindClusters with resolution = 2). scDblFinder (v1.8.0)^106^ was used to identify doublets. Clusters with >40% of the RNA profiles classified as “doublet” by scDblFinder were removed, and any remaining RNA profiles classified as “doublet” were also removed from the Seurat object. Next, clusters with high expression of marker genes that are known to be mutually exclusive were removed to eliminate additional doublet clusters. The remaining RNA profiles were re-clustered (DefaultAssay set to “integrated”FindNeighbors with reduction = “pca”, and dims = 1:20, FindClusters with resolution = 0.8).

To remove clusters with a low proportion of intron-containing reads, we analyzed a representative end-stage sample (“3nextseqs_11_18/3nextseq_run_11_8_endpoint_female”). Raw count matrices were generated using cellranger count (Cell Ranger v7.1.0, 10x Genomics) with the mm10–2020-A reference transcriptome, with the—include-introns flag set to true and false. The resulting matrices were processed using Read10X and CreateSeuratObject. For each barcode, the proportion of intronic reads (prop_intronic) was calculated as the difference between total (exonic + intronic) and exonic-only UMI counts, divided by the total. Clusters with a median prop_intronic below 0.4 were excluded from further analysis. The final clusters were manually annotated in the cell_class metadata column using the expression of known marker genes.^24,26^

#### snRNA-seq: Cholinergic Neuron and Alpha Motor Neuron Subclustering

A Seurat object containing data from cholinergic neurons was created using the nuclei annotated as “Cholinergic Neurons” from the above analysis. Variable features were identified (DefaultAssay set to “RNA”, FindVariableFeatures), and subclustering was performed (DefaultAssay set to “integrated”, ScaleData, RunPCA, RunUMAP with reduction = “pca” and dims = 1:13, FindNeighbors with reduction = “pca” and dims = 1:13, FindClusters with resolution = 0.4). The resulting clusters were manually annotated in the cholinergic_type metadata column using the expression of known marker genes from mouse cholinergic neurons.^24,28^ Another Seurat object containing data from alpha motor neurons was created using the nuclei annotated as “Alpha MNs” from the previous analysis of cholinergic neurons. Variable features were identified (DefaultAssay set to “RNA”, FindVariableFeatures), and subclustering was performed (DefaultAssay set to “integrated”, ScaleData, RunPCA, RunUMAP with reduction = “pca” and dims = 1:12, FindNeighbors with reduction = “pca” and dims = 1:12, FindClusters with resolution = 0.7).

#### snRNA-seq: Differential Expression and Gene Ontology (GO) Enrichment Analyses

DESeq2 (v1.32.0)^110^ was used to perform pseudobulk differential expression analyses. For each analysis, SoupX-adjusted counts rounded to the nearest integer were aggregated by gene across all nuclei of a given type for each experiment/biological replicate. All pseudobulk replicates contained data from at least 30 nuclei. For analyses in figure panels labeled “Downsampled,” nuclei were randomly subsampled within each cholinergic type to equalize cell numbers per replicate prior to aggregation, thereby controlling for differences in cell type abundance. The resulting genes-by-replicates count matrix was used to create a data frame of sample information, which included the condition of interest for the differential expression analysis (ex: stage information or cell type). The count matrix and sample information were used as input into DESeqDataSetFromMatrix with design set to the condition of interest. The resulting object was used as input into the DeSeq function (minReplicatesForReplace = Inf) to perform differential expression analysis using the Wald test to compute statistical significance and the Benjamini-Hochberg method to correct for testing of multiple hypotheses. The differential expression results were extracted, and genes with an adjusted *p* value of NA were removed. Enrichr (https://maayanlab.cloud/Enrichr/)^113,114^ was used to perform GO enrichment analysis. Significantly differentially expressed genes (padj < 0.01) that were upregulated or downregulated were used as the input gene set, and the background gene set was all genes from the differential expression analysis that had a numerical adjusted *p* value. Results from the GO Biological Process 2025 analysis were used. These analyses used the Fisher’s exact test to compute statistical significance and the Benjamini-Hochberg method to correct for testing of multiple hypotheses.

#### Multiome Sequencing: Data Pre-Processing

Sequencing reads for five samples were demultiplexed and aligned to a custom mouse mm10-arc reference, which allows mutant hSOD1 gene expression to be detected (10x Genomics), using the cellranger-arc mkfastq and cellranger-arc count pipelines (Cell Ranger ARC v2.0.1, 10x Genomics). Subsequent analyses were performed in R (v4.1.1) using the ArchR package (v1.0.2)^46^ unless otherwise noted. Arrow files were generated from ATAC fragment files (createArrowFiles, default parameters) and used to construct an ArchRProject. To incorporate gene expression data, filtered gene-barcode matrices from each sample were imported (import10xFeatureMatrix) and added to the ArchRProject (addGeneExpressionMatrix). Samples were derived from a mixture of both SOD1-G93A mice of a given stage and sex and control mice of the opposite sex. Each nucleus was assigned to the appropriate condition using counts of hSOD1 and sex-specific genes (*Xist*, *Uty*). Nuclei with ambiguous or conflicting marker gene expression were excluded.

#### Multiome Sequencing: Clustering and Doublet Removal

Dimensionality reduction was performed on the ArchR project from above using the addIterativeLSI function on both the ATAC (clusterParams resolution = 0.2) and RNA modalities (clusterParams resolution = 0.2, useMatrix = “GeneExpressionMatrix”, depthCol = “Gex_nUMI”, varFeatures = 2500, firstSelection = “variable”, binarize = FALSE). A combined LSI representation was then computed from the ATAC and RNA LSI embeddings (addCombinedDims). UMAP embeddings were generated separately on the ATAC, RNA, and combined dimensions (addUMAP with minDist = 0.8), and clustering was performed on the combined LSI dimensions (addClusters with resolution = 0.4). Next, two clusters with high expression of marker genes that are known to be mutually exclusive were removed. The remaining clusters were renamed and then manually annotated in the cell_class metadata column using the expression of known marker genes.

#### Multiome Sequencing: Cholinergic Neuron and Alpha Motor Neuron Subclustering

An ArchR project containing data from cholinergic neurons was created using the nuclei annotated as “Cholinergic Neurons” from the above analysis. Dimensionality reduction was performed on the resulting ArchR project using the addIterativeLSI function on both the ATAC (clusterParams resolution = 0.2) and RNA modalities (clusterParams resolution = 0.2, useMatrix = “GeneExpressionMatrix”, depthCol = “Gex_nUMI”, varFeatures = 2500, firstSelection = “variable”, binarize = FALSE). A combined LSI representation was then computed from the ATAC and RNA embeddings (addCombinedDims). UMAP embeddings were generated separately on the ATAC, RNA, and combined dimensions (addUMAP with minDist = 0.5), and clustering was performed on the combined LSI dimensions (addClusters with resolution = 0.5). One cluster with high expression of marker genes that are known to be mutually exclusive among cholinergic neurons was removed. The remaining clusters were renamed and then manually annotated in the cholinergic_type metadata column using the expression of known marker genes.

A similar approach was taken to the one above to create an ArchR project containing data from alpha motor neurons, with a few exceptions (addIterativeLSI: clusterParams resolution was left at the default value; addUMAP: minDist = 0.4). Clustering was performed using the ATAC-based LSI (addClusters with resolution = 1.2, nOutlier = 10), and the resulting clusters were manually annotated in the alpha_subtype metadata column using the expression of known marker genes and DM signature genes.

#### Multiome Sequencing: Cholinergic Neuron Peak Calling with Downsampled Datasets

To generate a downsampled ArchR project of control cholinergic neurons, only nuclei annotated as “control” in the Stage metadata column were retained. A new metadata column was created by combining sample identity and cholinergic neuron type (e.g., Alpha MNs, Gamma MNs, Gamma* MNs, etc.). For each sample, the number of nuclei per cholinergic subtype was summarized, and the minimum number of nuclei among subtypes was determined. Nuclei were then randomly downsampled within each sample and subtype to the appropriate minimum count to ensure equal representation of cholinergic subtypes across samples. The resulting set of nuclei was used to subset the original cholinergic neuron ArchR project, producing a downsampled control dataset for downstream comparisons.

The downsampled control ArchR project was split into individual ArchR projects by cholinergic neuron type (Alpha MNs, Gamma MNs, Gamma* MNs, Gad1+ cholinergic interneurons, Pitx2+ cholinergic interneurons, and Visceral MNs). For each subtype-specific project, pseudobulk replicates were generated by sample (addGroupCoverages with groupBy = “Sample”, minCells = 38, and minReplicates = 3). Peaks were then identified separately for each subtype using addReproduciblePeakSet with MACS2 (groupBy = “Sample”), and peak matrices were added with addPeakMatrix. These subtype-specific ArchR projects were subsequently used to quantify the number of peaks and to calculate the fraction of reads in peaks (FRIP) for each cholinergic neuron type.

The downsampled control ArchR project was used to create pseudobulk replicates (addGroupCoverages with groupBy = “cholinergic_type”, minCells = 38, and minReplicates = 3). Peaks were then called using addReproduciblePeakSet (groupBy = “cholinergic_type”), which used MACS2, and the peak matrix was added (addPeakMatrix). To quantify chromatin accessibility at single-nucleus resolution, the peak-by-cell accessibility matrix (PeakMatrix) was retrieved from the downsampled control ArchR project (getMatrixFromProject). For each nucleus, the number of accessible peaks was defined as the number of peaks with ≥1 fragment. To identify marker peaks across cholinergic neuron subtypes, differentially accessible peaks (marker peaks) were identified (getMarkerFeatures with groupBy = “cholinergic_type”, useMatrix = “PeakMatrix”, and testMethod = “wilcoxon”).

To compare chromatin accessibility changes with disease for motor neuron subtypes, alpha, gamma, gamma*, and visceral motor neurons were subset from the full cholinergic neuron ArchR project. Gamma and gamma* neurons were grouped as Pan-Gamma MNs, and individual ArchR projects were created for the three final subtypes: Alpha MNs, Pan-Gamma MNs, and Visceral MNs. For each sample/disease stage, the minimum number of nuclei among the three subtypes was computed, and nuclei were randomly downsampled within each sample/stage to match the appropriate minimum value, ensuring balanced representation across subtypes. Pseudobulk replicates were then created for each downsampled, subtype-specific ArchR project (addGroupCoverages with groupBy = “Stage” and minCells = 89). Peaks were called using addReproduciblePeakSet (groupBy = “Stage”), and the peak matrix was added (addPeakMatrix). Differentially accessible peaks were identified by comparing mid/end-stage SOD1-G93A motor neurons to controls for each subtype (getMarkerFeatures with groupBy = “Stage”, useGroups = “mid-late”, bgdGroups = “control”, useMatrix = “PeakMatrix”, testMethod = “wilcoxon”, and maxCells = 574).

#### Multiome Sequencing: Alpha Motor Neuron Differentially Accessible Peaks with Disease

With the non-downsampled alpha motor neuron ArchR project from above, pseudobulk replicates were made (addGroupCoverages with groupBy = “Stage”), peaks were called (addReproduciblePeakSet with groupBy = “Stage”), and the peak matrices were added (addPeakMatrix). Differentially accessible peaks were identified by comparing mid/end-stage SOD1-G93A alpha motor neurons to controls (getMarkerFeatures with groupBy = “Stage”, useGroups = “mid-late”, bgdGroups = “control”, useMatrix = “PeakMatrix”, testMethod = “wilcoxon”, and maxCells = 1052).

#### Multiome Sequencing: Identification of Positive Transcription Factor Regulators

Positive transcription factor (TF) regulators were identified for control skeletal motor neuron subtypes (alpha and pan-gamma), control alpha motor neuron subtypes (fast-firing, intermediate, and slow-firing), and alpha motor neuron disease status (non-DM, early DM, late DM). For each of these three analyses, an ArchR project containing the appropriate nuclei was created. With each ArchR project, pseudobulk replicates were made (addGroupCoverages with groupBy set appropriately), peaks were called (addReproduciblePeakSet with groupBy set appropriately), and the peak matrices were added (addPeakMatrix). Motif annotations were then added for each peak (addMotifAnnotations with motifSet = “cisbp”). Background peaks were generated (addBgdPeaks), and motif deviations and deviation z-scores were computed (addDeviationsMatrix with peakAnnotation = “Motif”). These data were aggregated by groups of interest (getGroupSE with useMatrix = “MotifMatrix” and groupBy set appropriately) and subsetted to just the deviation z-score data. For each motif, the maximum delta in z-score across groups (“maxDelta”) and the correlation between the motif accessibility and gene expression of the associated TF (correlateMatrices with useMatrix1 = “GeneExpressionMatrix”, useMatrix2 = “MotifMatrix”, and reducedDims = “LSI_Combined”) were calculated. The maxDelta information from above was added to the resulting correlation data frames. Motifs/TFs were labeled as “positive TF regulators” if they met the following criteria: correlation > 0.5, adjusted *p*-value < 0.01, and maxDelta above the 75th percentile.

#### snRNA-seq/Multiome Sequencing: Label Transfer from Multiome to snRNA-seq Alpha Motor Neurons

The filtered count matrices containing the RNA data from the multiome experiments were imported (import10xFeatureMatrix function from ArchR). The resulting matrix was filtered to contain data from nuclei that are present in the alpha motor neuron ArchR project. The filtered matrix was used to create a Seurat object (CreateSeuratObject), and metadata (including alpha_subtype, which contains the firing properties/DM status information) from the alpha motor neuron ArchR project was added to the Seurat object (AddMetaData). The Seurat object was log-normalized (NormalizeData), and variable features were identified (FindVariableFeatures with selection.method = “vst” and features = 2000). To transfer the alpha_subtype labels to the RNA only alpha motor neuron Seurat object, transfer anchors were identified (FindTransferAnchors with reference set to the multiome RNA Seurat object, query set to the RNA only alpha motor neuron object, reference.assay = “RNA”, query.assay = “RNA”, dims = 1:50), and predictions were made (TransferData using the above anchorset, refdata set to the alpha_subtype metadata field from the multiome RNA Seurat object, dims = 1:50). These predictions were added as metadata (AddMetaData) to the RNA-only alpha motor neuron Seurat object.

#### snRNA-seq/Multiome Sequencing: *In Silico* Transcription Factor Perturbation with CellOracle

Transcription factor (TF) regulators of alpha motor neuron subtypes were nominated using Python (v3.10) and CellOracle (v0.18.0),^47^ with analyses conducted separately for (1) control alpha motor neuron subtypes (fast-firing, intermediate, slow-firing) and (2) alpha motor neurons grouped by disease status (non-DM, early DM, late DM). To generate input for CellOracle, the snRNA-seq dataset (with subtype labels transferred from the multiome reference) was converted to AnnData format and pre-processed using Scanpy (v1.10.0). Pre-processing included gene filtering (minimum 1 count per gene using scanpy.pp.filter_genes), total count normalization (scanpy.pp.normalize_total), log transformation (scanpy.pp.log1p), selection of 2,000 highly variable genes (scanpy.pp.highly_variable_genes with flavor = “seurat”), PCA and neighbor graph construction (scanpy.tl.pca, scanpy.pp.neighbors), and UMAP embedding (scanpy.tl.umap). Discrete clusters that were not connected to the main alpha motor neuron population were removed.

To estimate pseudotime trajectories, a Pseudotime_calculator object (Pt) was created for each analysis using CellOracle, with inputs including the UMAP coordinates and cluster labels. Lineages were manually defined (Pt.set_lineage), and root cells were selected (Pt.set_root_cells). Diffusion maps were computed with Scanpy (scanpy.tl.diffmap), and pseudotime values were calculated with get_pseudotime_per_each_lineage.

To prepare the data for regulatory network inference, a prebuilt mouse scATAC-seq-derived base GRN was imported (load_mouse_scATAC_atlas_base_GRN) as base_GRN, and Oracle objects were then initialized using raw count matrices, annotated cluster labels, and UMAP coordinates. The base GRN was then incorporated via oracle.import_TF_data. To reduce dimensionality and enable efficient k-nearest neighbors (KNN) imputation, PCA was performed (oracle.perform_PCA), and the number of components was selected based on a variance threshold, capped at 50. KNN imputation (oracle.knn_imputation) was applied using 2.5% of total cells as k (balanced = True, b_sight = k × 8, and b_maxl = k × 4).

Next, gene regulatory networks (GRNs) were inferred for each cell group (e.g., each control subtype or disease-status group) using oracle.get_links to generate Links objects (links). The resulting networks were filtered to retain high-confidence regulatory interactions (links.filter_links with *p* = 0.001, threshold_number = 10,000, weight = “coef_abs”). Group-specific TF dictionaries were then derived (oracle.get_cluster_specific_TFdict_from_Links) and used to fit the GRNs for downstream perturbation simulations (oracle. fit_GRN_for_simulation, alpha = 10, use_cluster_specific_tfdict = True).

To define a reference cell state trajectory, previously computed pseudotime values were transferred to the Oracle object (oracle.adata.obs[“Pseudotime”] = Pt.adata.obs.Pseudotime). A Gradient_calculator object (gradient) was initialized using this pseudotime key. Cell density was estimated across a regular grid (gradient.calculate_p_mass with smooth = 0.8, n_grid = 40, and n_neighbors = 200), and low-density regions were filtered (gradient.calculate_mass_filter, min_mass = 6.3 for the control subtype analysis and 8.3 for the disease the analysis). The pseudotime values were mapped onto the grid using polynomial regression (gradient.transfer_data_into_grid with args = {“method”: “polynomial”, “n_poly”: 3}), and the gradient was computed (gradient.calculate_gradient), producing a reference vector field of unperturbed cell state transitions.

To evaluate the effect of TF perturbations, *in silico* knockout (KO) simulations were performed for all active regulatory TFs identified from the inferred GRNs (oracle.active_regulatory_genes). For each TF, CellOracle was used to simulate KO by setting the gene’s expression to zero (oracle.simulate_shift with n_propagation = 3). Cell state transitions were modeled using estimated transition probabilities (oracle.estimate_transition_prob with n_neighbors = 200, knn_random = True, and sampled_fraction = 1) and embedding shifts (oracle.calculate_embedding_shift with sigma_corr = 0.05). An Oracle_development_module object (dev) was instantiated for each TF, and the reference pseudotime gradient was loaded (dev.load_differentiation_reference_data). The simulated perturbation results were then imported (dev.load_perturb_simulation_data). Alignment between the KO vector field and the reference gradient was quantified by computing an inner product score (dev.calculate_inner_product), which was then discretized into 10 bins (dev.calculate_digitized_ip) to generate a perturbation score. Perturbation scores were analyzed using Oracle_systematic_analysis_helper, with statistical testing performed via calculate_positive_ps_p_value and calculate_negative_ps_p_value.

#### *In Vitro* Motor Neuron Differentiation, Lentiviral Transduction, Western Blot Analysis, Bulk RNA Sequencing, and Gene Set Enrichment Analysis

Human WTC11 induced pluripotent stem cells (iPSCs) harboring doxycycline-inducible NGN2, ISL1, and LHX3 (hNIL) transgenes were maintained and differentiated into motor neurons as previously described,^100,121^ with minor modifications. On Day 0, iPSCs were dissociated with Accutase and plated on Matrigel-coated plates in mTeSR Plus supplemented with 10 μM ROCK inhibitor. On Day 1, medium was replaced with neural induction medium (DMEM/F12 supplemented with N2, non-essential amino acids, and GlutaMAX) containing 2 μg/mL doxycycline and 0.2 μM γ-secretase inhibitor (Compound E).

On Day 3, cells were dissociated and replated onto poly-D-lysine (0.1 mg/mL) and laminin-coated 12-well plates (2 × 10^5^ cells per well) in neural induction medium supplemented with laminin (1 μg/mL) and BrdU (40 μM). Aphidicolin (10 μM) was added beginning on Day 3 and maintained at each media change to limit proliferation of mitotically active cells. From Days 4–9, cultures were maintained in DMEM/F12-based neural induction medium supplemented with B27, CultureOne, laminin (1 μg/mL), and BDNF/GDNF/NT3 (20 ng/mL each), with half-media changes performed every 1–2 days. On Day 10, cultures were transitioned to Neurobasal-based maturation medium supplemented with B27, CultureOne, laminin (500 ng/mL), and BDNF/GDNF/NT3 (20 ng/mL each), with half-media changes every 3–4 days thereafter.

On Day 16, motor neurons were transduced with lentivirus encoding CREB3-V5, ATF3, or mCherry control. Plasmids were obtained from Addgene (CREB3-V5, #144787; ATF3, #141707; mCherry, #145026),^101^ and lentivirus was produced by the Gene Vector and Virus Core (GVVC) at Stanford University. Transductions were performed at a targeted multiplicity of infection (MOI) of 2, with virus applied directly to the wells. Half-media changes continued as described above. RIPA-soluble protein was collected 14 days post-transduction for Western blot analysis using the Bio-Rad Trans-Blot Turbo Transfer System and EveryBlot Blocking buffer. Primary antibodies were obtained from Cell Signaling Technology (V5-Tag (D3H8Q), #13202; ATF-3 (D2Y5W), #33593).

RNA was also harvested 14 days post-transduction using Trizol reagent (Invitrogen, 15596026) following the manufacturer’s instructions for bulk RNA sequencing. For each condition, three independent wells were transduced and processed as technical replicates. Libraries were prepared using SMARTer Stranded Total RNA-Seq Kit v2 (TaKaRa, 634411), starting with 20 ng total RNA per sample, following the manufacturer’s protocol. The libraries were then quantified, pooled, and sequenced on a NovaSeq X in 150 bp paired-end mode (Illumina). Analysis was performed following the nf-core/rnaseq workflow. Briefly, adapters in FASTQ files were trimmed using TrimGalore. The adapter-trimmed FASTQ files were then mapped to the human genome (hg38) using STAR, following ENCODE’s recommended settings. Transcript abundance was quantified with Salmon, and differential gene expression analysis was performed using DESeq2. Gene set enrichment analysis was performed with the fgsea R package (v1.20.0).^112^ DM-up and DM-down gene sets were defined from mouse differential expression results (padj < 0.01, log2FC > 0 or < 0), mapped to human orthologs using the biomaRt R package (v2.50.3), and restricted to uniquely mapped protein-coding genes. Genes were ranked by DESeq2 Wald statistics, retaining one value per gene (maximum absolute statistic where multiple entries per gene were present), and enrichment was assessed using fgseaMultilevel (minSize = 15, maxSize = 5000). Effect sizes were reported as normalized enrichment scores (NES), and significance was assessed using permutation-based *p* values with Benjamini-Hochberg (FDR) correction.

#### MERFISH: Sample Collection and Imaging

For collection of fresh frozen spinal cord samples, mice were deeply anesthetized and then perfused with PBS. Following perfusion, the spinal cords were hydraulically extruded as described above or manually dissected. The tissue from each cord was cut into three or four small pieces centered around the cervical enlargement and another three to four small pieces centered around the lumbar enlargement. The cervical and lumbar tissue pieces were placed into respective plastic cryomolds that contained pre-chilled optimal cutting temperature (OCT) compound. Using forceps, each cryomold was transferred into an isopentane and liquid nitrogen bath such that the cryomold made contact with the isopentane but was not submerged. The cryomolds maintained contact with the isopentane until the OCT had solidified. The samples were then stored at −80°C until the MERFISH experiments were performed.

The MERFISH experiments were conducted through the Vizgen MERSCOPE technology laboratory service. One MERSCOPE run contained only control, lumbar tissue (~3–4 tissue sections from tissue pieces embedded together as described above), and the 18 additional runs contained both SOD1-G93A tissue at a given disease stage and age-matched control tissue (~3–4 tissue sections each) per MERSCOPE run. Of these 18 runs, six contained early-stage SOD1-G93A tissue, six contained mid-stage SOD1-G93A tissue, and six contained end-stage SOD1-G93A tissue. Of the six runs per disease stage, two runs contained tissue centered around the cervical enlargement and four runs contained tissue centered around the lumbar enlargement. The full sample preparation user guide is available at https://vizgen.com/resources/fresh-and-fixed-frozen-tissue-sample-preparation/. For imaging, a custom gene panel for 140 genes was used. Additionally, *Apoe* and *Gfap* were included as sequential genes for all runs. For runs containing early or mid-stage SOD1-G93A tissue, hSOD1-G93A was also included as a sequential gene. The full instrument user guide is available at https://vizgen.com/resources/merscope-instrument/.

#### MERFISH: Generation of a Custom Motor Neuron Segmentation Model

A custom motor neuron segmentation model was generated using the Cellpose3 (v3.1.0) graphical user interface.^115,122^ First, to generate training data, input images were created for each MERFISH experiment by plotting relevant transcripts as 10-pixel-diameter circles at their appropriate spatial coordinates with a custom Python script. One image included motor neuron/DM markers (*Chat*, *Prph*, *Slc5a7*, *Atf3*) in green, and a second image included non-motor neuron markers (*Aldh1l1*, *Aqp4*, *Cx3cr1*, *Gad1*, *Mog*, *Slc17a6*, *Slc6a5*, *Trem2*) in red. These input images were used with the Vizgen Post-processing Tool (vpt, v1.3.0) to extract 600 × 600 μm image patches from selected locations across spinal cord tissue sections and experiments (vpt extract-image-patch). The resulting image patches were used to manually trace motor neurons using the Graphic app (Picta) on an iPad (Apple). The manually traced motor neuron masks were post-processed using a custom ImageJ macro to create Cellpose-compatible segmentation masks. The image patches and masks were then used to train a new Cellpose model (initial model: cyto3, chan to segment: green, chan2: red, learning_rate: 0.1, weight_decay: 0.0001, n_epochs: 500). The resulting model was validated visually on held-out images to confirm accurate segmentation of large cholinergic motor neurons and was used for downstream segmentation tasks.

#### MERFISH: Cell Segmentation, Transcript Partitioning, and Cell Metadata Calculation

Cell segmentation, transcript partitioning, and cell metadata calculation were performed using the Vizgen Post-processing Tool (vpt, v1.3.0) with the Cellpose2 plugin (vpt-plugin-cellpose2, v1.0.1).^123^ To segment motor neurons and other cell types, a custom two-task segmentation algorithm was used. For motor neuron segmentation, the custom model described above was applied to full-experiment input images showing motor neuron and non-motor neuron transcript locations. For segmentation of other cells, a standard Cellpose2 “nuclei” model was applied using the DAPI channel. Segmentation outputs from the two tasks were combined using a “harmonize” fusion strategy to produce a complete, non-overlapping set of cell boundaries. This segmentation algorithm was used to identify cell boundaries for each MERFISH experiment (vpt run-segmentation with a tile size of 7000 and tile overlap of 900). After segmentation, transcripts were partitioned into cells (vpt partition-transcripts), and then cell metadata was calculated (vpt derive-entity-metadata, vpt sum-signals).

#### MERFISH: Data Pre-Processing and Initial Clustering

Subsequent MERFISH analyses were performed in Python (v3.9.21) using the Scanpy package (v1.10.3).^104^ For each MERFISH experiment, an AnnData object was created by combining gene expression data, cell metadata, and signal intensities for the sequential genes (*Apoe*, *Gfap*, and hSOD1-G93A). To account for multiple tissue sections within a single experiment, each cell was assigned to a specific section based on its spatial coordinates. Cells that did not fall within any defined tissue section were excluded. For cells within valid sections, additional metadata was added, including section ID, slide ID, anatomical region, disease stage, and experiment batch (VS119 or VS223). For each MERFISH experiment, genes labeled as “blank” were removed as well as cells with 20 or fewer transcripts or five or fewer detected genes. The filtered AnnData objects were then concatenated into a single dataset with raw counts stored in .X and duplicated in .layers[“counts”]. Additionally, a few low-quality tissue sections were removed based on manual inspection.

To account for cell size differences, sequential gene signals and raw gene expression values were normalized by cell volume. Total gene expression for each cell was then normalized to 250 (scanpy.pp.normalize_total), and expression values were log-transformed with a pseudocount (scanpy.pp.log1p). The log-normalized gene expression values were subsequently converted to z-scores (scanpy.pp.scale with max_value = 10), and principal component analysis (PCA) was performed for dimensionality reduction (scanpy.tl.pca). A batch-balanced k-nearest neighbors graph was then constructed using BBKNN (scanpy.external.pp.bbknn), with experiment batch (VS119 or VS223) specified as the batch key. This graph was used to compute a UMAP embedding for visualization (scanpy.tl.umap). Leiden clustering was then performed (scanpy.tl.leiden with resolution = 0.5, flavor = “igraph”, and n_iterations = 2), and the resulting clusters were manually annotated based on the expression of cell class marker genes and their spatial locations.

#### MERFISH: Cholinergic Neuron and Alpha Motor Neuron Subclustering

To subcluster cholinergic neurons, cells labeled as “Cholinergic Neurons” were subset from the full AnnData object. Batch correction across experiment sets (VS119 and VS223) was performed using BBKNN (scanpy.external.pp.bbknn), followed by UMAP embedding (scanpy.tl.umap with min_dist = 0.1) and Leiden clustering (scanpy.tl.leiden with resolution = 1.0, flavor = “igraph”, and n_iterations = 2). Clusters enriched for *Mog* expression were identified and evaluated as putative peri-motor neuron oligodendrocytes. To assess this, the volume distribution of these cells was compared to that of high-confidence oligodendrocytes from the full dataset using violin plots. Interquartile range (IQR)-based whisker bounds were used to define the expected volume range for oligodendrocytes. Cells falling within this range were reclassified as oligodendrocytes, while those outside the range were removed. The remaining cholinergic neurons were then reanalyzed: BBKNN and UMAP were recomputed, and Leiden clustering was repeated with increased resolution (resolution = 1.2). The resulting clusters were manually annotated using the expression of known cholinergic neuron marker genes. Finally, cells with a volume less than 1000 were removed to exclude low-volume cholinergic neurons. To subcluster alpha motor neurons, cells labeled as “Alpha MNs” were subset from the cholinergic neuron dataset. BBKNN (scanpy.external.pp.bbknn), UMAP (scanpy.tl.umap), and Leiden clustering (scanpy.tl.leiden with resolution = 0.45, flavor = “igraph”, and n_iterations = 2) were then applied to identify subclusters.

#### MERFISH: Label Transfer from snRNA-seq to MERFISH Alpha Motor Neurons

To annotate alpha motor neuron subtypes in the MERFISH dataset, label transfer was performed using the alpha motor neuron snRNA-seq dataset in AnnData format as a reference. Subtype labels (“Fast-Firing”, “Intermediate”, “Slow-Firing”, “Early DM”, and “Late DM”) were originally defined in the multiome dataset and subsequently transferred to the snRNA-seq dataset, as described above, prior to use as the reference. Both the MERFISH and snRNA-seq datasets were filtered to include only shared genes, and a batch label was added to distinguish their origins. The datasets were concatenated and jointly embedded using scVI (scvi.model.SCVI, scvi-tools v1.1.6.post2).^107,108^ The SCVI model was trained on the combined dataset with batch correction (SCVI.setup_anndata and SCVI.train). Cell type label transfer was then performed using SCANVI, a semi-supervised extension of scVI.^108,109^ The SCANVI model was initialized from the pretrained SCVI model (SCANVI.from_scvi_model) and trained using the snRNA-seq annotations as the labeled reference and the MERFISH cells as unlabeled (SCANVI.train with max_epochs = 400). Predicted cell type labels were inferred for each MERFISH alpha motor neuron (SCANVI.predict) and stored in the alpha motor neuron AnnData object.

#### MERFISH: Alpha Motor Neuron Differential Expression Analysis with Disease

To identify differentially expressed genes in alpha motor neurons, a set of disease-associated genes previously defined in the snRNA-seq analysis (control vs. end-stage; adjusted *p*-value < 0.01) was intersected with the MERFISH gene panel. The resulting gene set was used to subset the MERFISH alpha motor neuron dataset, and raw expression values were retrieved from the counts layer. Expression was then normalized by cell volume, scaled to 250 total counts per cell (scanpy.pp.normalize_total), and log-transformed with a pseudocount (scanpy.pp.log1p). Pairwise differential expression analysis was performed between control vs. mid-stage and control vs. end-stage alpha motor neurons using two-sided Mann-Whitney U tests. Fold changes were computed on the linear (non-log-transformed) scale and then log2-transformed, and adjusted *p*-values were calculated using the Benjamini-Hochberg procedure (statsmodels.stats.multitest.multipletests).

#### MERFISH: Dataset Refinement and Spatial Standardization

An updated version of the full MERFISH dataset was generated to refine and extend cell type annotations. To do so, subsets of the original AnnData object were selectively replaced: peri-motor neuron oligodendrocytes were reassigned to the “oligodendrocytes” class, and all cholinergic neurons were removed and replaced with the filtered dataset excluding low-volume cells and oligodendrocyte contaminants. Alpha motor neurons were replaced with a label-transferred dataset containing predicted subtypes, and a DM_status column was added to classify them as “DM” or “non-DM.” To standardize section orientation, as done in Sun et al.,^124^ spatial coordinates were mean-centered and rotated using section-specific angles determined by visual inspection. Updated coordinates were stored in adata.obsm[“spatial”].

#### MERFISH: Tissue Section Filtering for Spatial Analyses

For analyses sensitive to tissue integrity, such as spatial neighborhood and relative abundance analyses, sections were further filtered to exclude sections with tears. Each tissue section was manually reviewed and labeled as “keep,” “remove,” “left,” or “right.” Sections labeled “keep” were retained in full, while left and right halves were extracted from “left” and “right” sections, respectively. Cells from “keep,” left-filtered, and right-filtered sections were merged to generate a high-quality spatial dataset for downstream analysis.

#### MERFISH: Distance Calculation and Nearest Neighbor Determination

All distances were calculated as Euclidean distances between segmentation-derived cell centroids. For nearest-neighbor analyses, the six nearest neighbors of each motor neuron were identified and ranked from first to sixth.

#### MERFISH: Reactive Glial Cell Classification

To identify reactive glial cells across disease stages, per-experiment expression thresholds were computed based on control-stage tissue. For each slide (excluding one control-only slide with no disease tissue), the 95th percentile of *Apoe* expression among control microglia/macrophages and of *Gfap* expression among control astrocytes was calculated. These values were computed using high-pass filtered and normalized expression values. Cells were labeled based on these control-derived thresholds: microglia/macrophages with elevated *Apoe* were classified as reactive, and astrocytes with elevated *Gfap* were classified as reactive or white matter astrocytes.

#### MERFISH: Alpha Motor Neuron Morphological Quantification

Morphological features (soma volume, anisotropy, solidity, and perimeter-to-area ratio) were computed from MERFISH-derived cell segmentation boundaries for alpha motor neurons. To enrich for motor neuron segmentations containing nuclei for morphology analyses, DAPI signal intensity (DAPI_high_pass) was log10-transformed (log10[x + 1]), and an experiment-wide threshold was determined using Otsu’s method (skimage.filters.threshold_otsu). Segmentations with log-transformed DAPI signal greater than or equal to the Otsu threshold were classified as nucleus-containing cells and were retained for morphometric quantification. This filtering step was applied exclusively for morphology analyses and was not used for clustering, label transfer, or differential expression analyses.

#### snRNA-seq: Cell-Cell Communication Analysis with CellChat

To identify disease-associated ligand-receptor interactions, we performed cell-cell communication analysis using the CellChat package (v2.2.0)^73^ in R (v4.4.2). An expression matrix and metadata were first exported from a final snRNA-seq Seurat object that contained all high-quality data and annotations. Specifically, the normalized expression data were obtained with the Seurat function GetAssayData (assay = “RNA”, slot = “data”), and the full metadata were extracted from the meta.data. These were saved as .rds files and used as input for CellChat. For the CellChat analysis, the dataset was restricted to three cell types of interest (alpha motor neurons, astrocytes, and microglia/macrophages) and two conditions (control and end-stage).

For each condition (control and end-stage), a CellChat object was constructed from the filtered count matrix and cell metadata (createCellChat with group.by set to cell type labels). The ligand-receptor interaction database was restricted to the secreted signaling subset of CellChatDB.mouse (subsetDB with search = “Secreted Signaling” and key = “annotation”). For each CellChat object, the expression data were preprocessed (subsetData, identifyOverExpressedGenes with do.fast = FALSE, and identifyOverExpressedInteractions), and communication probabilities were computed (computeCommunProb with type = “truncatedMean” and trim = 0.1). Subsequently, signaling pathways were inferred (computeCommunProbPathway), the communication network was aggregated (aggregateNet), and network centrality scores were calculated (netAnalysis_computeCentrality).

The control and disease CellChat objects were then merged (mergeCellChat) for a comparative analysis. Differential expression between conditions was assessed using identifyOverExpressedGenes with the disease condition set as the positive dataset (only. pos = FALSE, thresh.pc = 0.1, thresh.fc = 0.1, thresh.p = 0.05, group.de.combined = FALSE, do.fast = FALSE). The differentially expressed gene information was mapped onto the inferred cell-cell communications (netMappingDEG), and ligand-receptor pairs showing significantly increased or decreased signaling in disease relative to control were extracted (subsetCommunication; either datasets set to the positive, disease dataset, ligand.logfc = 0.1, receptor.logfc = NULL or datasets set to the negative, control dataset, ligand.logfc = −0.1, receptor.logfc = NULL). To avoid ambiguity, ligand-receptor interactions appearing in both up- and downregulated sets were removed. Visualization of differentially regulated interactions was performed using the netVisual_bubble function, and bubble plots were generated for each source population (alpha motor neurons, astrocytes, microglia/macrophages) against other potential target populations.

Communication probability is estimated using a mass-action model based on average ligand and receptor expression, with statistical significance determined by permutation testing. Higher values indicate stronger expression-supported evidence of signaling between cell groups. “Upregulated” signaling in disease is defined based on increased ligand expression together with significant communication probability.

#### Human Spinal Cord snRNA-seq/Fragment-seq: Data Pre-Processing and Integration

Human spinal cord single-nucleus RNA-seq and fragment data were obtained from Cao et al. (2025).^83^ For the snRNA-seq data, analyses were performed starting from the motor neuron Seurat object generated by Cao et al., followed by additional quality control filtering to generate the final nucleus object for downstream analyses. Quality control filtering retained nuclei with nFeature_rna > 3361 and intronic_mapped_rate > 0.65 to enrich for high-quality neuronal nuclei and exclude damaged nuclei or cytoplasmic fragments. The nFeature_RNA threshold was guided by motor neuron profiles from our previous human spinal cord dataset,^22^ and the intronic_mapped_rate threshold was selected to exclude profiles with intermediate intronic rates that retained fragment-like features. Raw counts were normalized using NormalizeData with relative count (RC) normalization (normalization.method = “RC”, scale.factor = 10,000). The top 1,000 highly variable genes (HVGs) were selected using FindVariableFeatures with the vst method. Normalized expression values were scaled using ScaleData, followed by PCA using RunPCA. Samples were integrated using Harmony (HarmonyIntegration, SampleID as the integration variable) using the first 15 principal components and theta = 1.5.

For the fragment data, neuronal fragments were independently recovered from the original Cao et al. count matrices, as fragments were largely excluded from the processed objects. The count matrices were imported into R and converted into Seurat objects (Seurat v5)^103^ using the hdf5r package. Mitochondrial genes were removed from the fragment count matrix to generate an RNA_noMito assay, which was used for all downstream fragment analyses. Raw counts from the RNA_noMito assay were normalized using LogNormalize, and the top 2,000 HVGs were selected using FindVariableFeatures with the vst method. Normalized expression values were scaled using ScaleData, and PCA was performed using RunPCA. Samples were integrated using Harmony (HarmonyIntegration, SampleID as the integration variable) with the first 30 principal components. For the fragment data, initial filtering required fewer than 3,000 UMIs and a low intronic mapped rate (<40%) to ensure bona fide neuronal fragment identity prior to subclustering. The resulting neuronal fragment profiles were confirmed to be enriched for *NEFM* expression.

#### Human Spinal Cord snRNA-seq/Fragment-seq: Clustering and Motor Neuron Subtype Annotation

Following Harmony integration, nearest-neighbor graphs were constructed using FindNeighbors with the Harmony dimensions described above, followed by Louvain clustering using FindClusters (resolution = 0.5 for nuclei and resolution = 1 for fragments). Dimensionality reduction for visualization was performed using RunUMAP with the same dimensions. The nuclei data were subsequently normalized using LogNormalize. Subtypes of alpha, gamma, and visceral motor neurons, as well as cholinergic, excitatory, and inhibitory interneurons were annotated based on the robust expression of established marker genes (e.g., *SLC17A6*, *GAD1*, *VIPR2*, *PEX5L*, *AMTN*, *NOS1*).

#### Human Spinal Cord snRNA-seq/Fragment-seq: Differential Gene Expression Analysis

Differential expression analyses were performed separately for each nucleus/fragment type using raw counts with the Nebula R package (v1.5.6).^111^ Models compared disease status groups (SOD1, C9, and sALS) against neurologically healthy controls, with sample-specific effects modeled as (1 | SampleID). Batch and mitochondrial read fraction (pct_counts_mito) were included in the model as covariates. NEBULA-derived log fold-change and SE estimates were converted to base 2, Wald/z statistics were calculated as logFC/SE, and *p*-values were adjusted using the Benjamini-Hochberg (FDR) procedure for each disease-control comparison before use in downstream analyses and supplementary tables.

#### Human Spinal Cord snRNA-seq/Fragment-seq: Cross-Species Wilcoxon Rank-Based Gene Set Enrichment and DM Scoring

To compare human motor neuron responses to the mouse disease-associated motor neuron (DM) signature, murine genes were first mapped to human orthologs using the homologene R package (v1.4.68.19.3.27). The top 1,000 significantly upregulated and downregulated genes (padj < 0.01) from the mouse DM signature were selected by absolute log_2_ fold change after mapping to human orthologs, and gene set enrichment was assessed using one-sided Wilcoxon-Mann-Whitney rank-sum tests on NEBULA-derived Wald/z statistics (calculated as logFC/SE) for each nucleus and fragment type, with effect sizes reported as Hodges-Lehmann estimates of median shift with 95% confidence intervals.

For the TDP-43 cryptic target analysis, we curated a list of candidate genes whose cryptic splicing is regulated by TDP-43 from the literature. This panel incorporated proposed and validated TDP-43 cryptic targets *STMN2*, *UNC13A*, *CYFIP2*, *HDGFL2*, *AGRN*, *PFKP*, *SYT7*, *KALRN*, *ATG4B*, *ARHGAP32*, *ELAVL3*, *PRUNE2*, *NUP188*, *CAMK2B*, *CDK7*, *ATP8A2*, *RAP1GAP*, and *KCNQ2*_._^84,85,125–134^

To quantify disease states at the single-cell and fragment levels, a “Weighted DM Score” was developed. Human orthologs of the mouse DM signature were filtered for detectability (detected in >1% of profiles used in the corresponding analysis), and two gene panels were constructed: a DM-up panel (top 1,000 upregulated genes) and a DM-down panel (top 1,000 downregulated genes). The same selected DM-up and DM-down panels were used for all profiles within a given analysis. Weighted scores were calculated using log-normalized human expression values that were z-scored for each gene across profiles in the given analysis. For each profile c and gene panel G (either DM-up or DM-down), the score was calculated as Σg∈G(zg,c × |log_2_FCg|) / Σg∈G|log_2_FCg|, where zg,c is the z-scored expression value for gene g in profile c, and log_2_FCg is the mouse DM log_2_ fold-change for gene g. The final Delta Weighted DM Score was computed by subtracting the DM-down weighted score from the DM-up weighted score.

#### Human Spinal Cord snRNA-seq/Fragment-seq: Linear Mixed-Effects Models (LMMs) for Delta Weighted DM Scores

To assess statistical differences in Delta Weighted DM Scores across conditions (Control, SOD1, C9, sALS) or pooled conditions (Control vs. all ALS), linear mixed-effects models were fit separately for each nucleus or fragment type using the lme4 (v1.1–35.1)^119^ and lmerTest (v3.2–0) R packages.^120^ Models were fit using individual nuclei or fragments as observations, with condition or pooled condition as the fixed effect and SampleID as a random intercept to account for sample-specific baseline differences and within-sample correlation among profiles (Score ~ condition + (1 | SampleID)). Pairwise statistical significance and estimated marginal means were calculated using the emmeans R package (v2.0.1).

#### Partitioned Heritability and H-MAGMA Analyses

Partitioned heritability was estimated with linkage disequilibrium (LD) Score Regression (LDSC, v1.0.1) via the command line to evaluate enrichment of genome-wide association study (GWAS) signals within differentially accessible chromatin regions.^88,116^ Differentially accessible peaks (FDR < 0.05) were lifted over from the mm10 to the hg19 genome using the bnMapper function from the bx-python package (v0.12.0).^118^ After conversion, 250 bp padding was added to both sides of each peak, and blacklisted hg19 regions were removed.^89,135^ Annotation files were generated by identifying all HapMap3 single nucleotide polymorphisms (SNPs)^136^ located within the lifted-over peaks. LD scores were then calculated using default settings (LD window: 1cM, reference panel: 1000 Genomes European Phase 3).^137^ Partitioned heritability was estimated by including LD scores from both the baseline model and SNPs within all lifted-over differentially accessible peaks in the regression.^88^ GWAS summary statistics for ALS and other disorders were used to compute trait-specific partitioned heritability.^90–93^ Enrichment was defined as the proportion of heritability explained by SNPs in the annotation (i.e., lifted-over differentially accessible peaks) divided by the proportion of reference SNPs within the annotation. Enrichment *p*-values (Z-tests on regression coefficients) were FDR-corrected across the four GWAS datasets.

To identify putative genes underlying the ALS GWAS signal enrichment within lifted-over differentially accessible regions, H-MAGMA was performed using the most recent ALS GWAS, following the developer’s instructions.^94,95^ H-MAGMA extends conventional Multi-marker Analysis of GenoMic Annotation (MAGMA) by incorporating Hi-C-derived 3D chromatin interaction data to link intergenic SNPs to their potential target genes.^117^ Required input files, including exon and promoter coordinates, SNP coordinates, their overlaps, and adult brain Hi-C data, were downloaded from Zenodo (https://doi.org/10.5281/zenodo.5503876). Loop anchors in the Hi-C dataset were intersected with promoter regions to define anchor 1 sites. Intergenic SNPs located in the corresponding anchor 2 sites were then assigned to the gene whose promoter overlapped anchor 1. The resulting SNP-gene annotation table was then used for gene-level analysis with the SNP-wise model in MAGMA (v1.10).^117^ The final H-MAGMA output, containing *Z* statistics for each gene, was intersected with genes in the lifted-over differentially accessible peaks to identify putative ALS risk genes within regions of altered chromatin accessibility.

### QUANTIFICATION AND STATISTICAL ANALYSIS

Statistical analyses were performed using R, Python, and the analysis software described in the relevant method details sections. Analysis-specific statistical tests, software and packages, multiple-testing corrections, *n* values, definitions of *n*, and measures of center, dispersion, and precision are reported in the corresponding method details sections and figure legends.

In brief, differential expression analyses for mouse snRNA-seq and *in vitro* bulk RNA-seq used DESeq2 with Wald tests and Benjamini-Hochberg FDR correction. MERFISH alpha motor neuron differential expression used two-sided Mann-Whitney U tests/Wilcoxon rank-sum tests with Benjamini-Hochberg FDR correction. Human snRNA-seq and fragment differential expression used NEBULA with SampleID included as a sample-level effect, Wald/z statistics calculated as logFC/SE, and *p* values corrected using the Benjamini-Hochberg FDR procedure. Gene set enrichment analyses used fgsea or one-sided Mann-Whitney U tests/Wilcoxon rank-sum tests, as described in method details. GO enrichment, gene-set overlap, spatial abundance, spatial neighborhood, morphology, and related analyses used the tests specified in method details and figure legends, including Fisher’s exact tests, Welch’s *t* tests, paired *t* tests, one-way ANOVA with Tukey’s honestly significant difference test, Mann-Whitney U tests/Wilcoxon rank-sum tests, paired Wilcoxon signed-rank tests, and Bonferroni or FDR correction where indicated. Cell-cell communication significance was assessed using CellChat permutation testing. Linear mixed-effects models were fit using lme4 and lmerTest, with pairwise comparisons and estimated marginal means calculated using emmeans. Partitioned heritability enrichment was assessed using LDSC Z-tests on regression coefficients, with FDR correction across GWAS datasets.

No formal tests were performed to assess whether data met assumptions of the statistical approaches. Statistical tests were selected based on the experimental design, data structure, and relevant comparison. Model-based methods appropriate for count data or for data with many nuclei/fragments per biological sample were used for sequencing and single-cell analyses, including DESeq2 for pseudobulk RNA-seq, NEBULA for human single-nucleus/fragment analyses, and linear mixed-effects models with SampleID as a random effect where individual nuclei/fragments were modeled as observations. Non-parametric tests, including Mann-Whitney U tests/Wilcoxon rank-sum tests and paired Wilcoxon signed-rank tests, were used when normality could not be assumed or rank-based inference was preferred. Welch’s *t* tests were used for comparisons in which equal variance between groups was not assumed.

## Supplementary Material

MMC2

MMC1

SUPPLEMENTAL INFORMATION

Supplemental information can be found online at https://doi.org/10.1016/j.cell.2026.05.047.

## Figures and Tables

**KEY RESOURCES TABLE:** 

REAGENT or RESOURCE	SOURCE	IDENTIFIER

Antibodies		

V5-Tag (D3H8Q) Rabbit Monoclonal Antibody	Cell Signaling Technology	Cat#13202; RRID:AB_2687461
ATF-3 (D2Y5W) Rabbit Monoclonal Antibody	Cell Signaling Technology	Cat#33593; RRID:AB_2799039

Critical commercial assays		

Chromium Single Cell 3'Reagent Kit v3	10x Genomics	N/A
Chromium Single Cell Multiome ATAC + Gene Expression v1	10x Genomics	N/A
SMARTer Stranded Total RNA-Seq Kit v2	Takara	Cat#634411

Deposited data		

Mouse spinal cord snRNA-seq dataset (control and SOD1-G93A)	This paper	GEO: GSE306676
Mouse spinal cord multiome (snATAC + snRNA-seq) dataset (control and SOD1- G93A)	This paper	GEO: GSE306675
Mouse spinal cord MERFISH dataset (control and SOD1-G93A)	This paper	Zenodo:https://doi.org/10.5281/zenodo.16938739
Mouse spinal cord snRNA-seq dataset (control)	Blum et al.^24^	GEO: GSE161621
Human spinal cord snRNA-seq dataset (Control and ALS)	Cao et al.^83^	10.1101/2025.08.26.672029

Experimental models: Cell lines		

WTC11 hNIL iPSC line	Fernandopulle et al.^100^ Kindly provided by Michael Ward and Christopher Grunseich	N/A

Experimental models: Organisms/strains		

ChAT-IRES- Cre mice	The Jackson Laboratory	Strain #:006410; RRID:IMSR_JAX:006410
ROSAnT-nG mice	The Jackson Laboratory	Strain #:023035; RRID:IMSR_JAX:023035
SOD1-G93A mice	The Jackson Laboratory	Strain #:002726; RRID:IMSR_JAX:002726
B6SJLF1/J mice	The Jackson Laboratory	Strain #:100012; RRID:IMSR_JAX:100012

Recombinant DNA		

CREB3-V5 plasmid (TFORF3311)	Joung et al.^101^	Addgene plasmid #144787; RRID:Addgene_144787
ATF3 plasmid (TFORF0801)	Joung et al.^101^	Addgene plasmid #141707; RRID:Addgene_141707
mCherry plasmid (TFORF3550)	Joung et al.^101^	Addgene plasmid #145026; RRID:Addgene_145026

Software and algorithms		

Cell Ranger (v3.1.0/v7.1.0)	10x Genomics	https://www.10xgenomics.com/support/software/cell-ranger/latest
Cell Ranger ARC (v2.0.1)	10x Genomics	https://www.10xgenomics.com/support/software/cell-ranger-arc/latest
Seurat (v4.0.4/v5)	Hao et al.^102^; Hao et al.^103^	https://satijalab.org/seurat/
Scanpy (v1.10.0/1.10.3)	Wolf et al.^104^	https://scanpy.readthedocs.io/en/stable/
ArchR (v1.0.2)	Granja et al.^46^	https://www.archrproject.com
SoupX (v1.6.2)	Young and Behjati^105^	https://github.com/constantAmateur/SoupX
scDblFinder (v1.8.0)	Germain et al.^106^	https://bioconductor.org/packages/release/bioc/html/scDblFinder.html
scvi-tools (v1.1.6.post2)	Lopez et al.^107^; Gayoso et al.^108^; Xu et al.^109^	https://scvi-tools.org
CellOracle (v0.18.0)	Kamimoto et al.^47^	https://morris-lab.github.io/CellOracle.documentation/
CellChat (v2.2.0)	Jin et al.^73^	https://github.com/jinworks/CellChat
DESeq2 (v1.32.0)	Love et al.^110^	https://bioconductor.org/packages/release/bioc/html/DESeq2.html
NEBULA (v1.5.6)	He et al.^111^	https://cran.r-project.org/web/packages/nebula/index.html
fgsea (v1.20.0)	Korotkevich et al.^112^	https://bioconductor.org/packages/release/bioc/html/fgsea.html
Enrichr	Kuleshov et al.^113^; Chen et al.^114^	https://maayanlab.cloud/Enrichr/
Cellpose3 (v3.1.0)	Stringer and Pachitariu^115^	https://cellpose.readthedocs.io/en/latest/
Vizgen Post-processing Tool (vpt) (v1.3.0)	Vizgen	https://vizgen.github.io/vizgen-postprocessing/index.html
LD Score Regression (LDSC) (v1.0.1)	Finucane et al.^88^; Finucane et al.^116^	https://github.com/bulik/ldsc
MAGMA (v1.10)	de Leeuw et al.^117^	https://cncr.nl/research/magma/
H-MAGMA	Sey et al.^94^; Sey et al.^95^	https://github.com/thewonlab/H-MAGMA
bx-python (v0.12.0)	Denas et al.^118^	https://github.com/bxlab/bx-python
lme4 (v1.1–35.1)	Bates et al.^119^	https://cran.r-project.org/web/packages/lme4/index.html
lmerTest (v3.2–0)	Kuznetsova et al.^120^	https://cran.r-project.org/web/packages/lmerTest/index.html
emmeans (v2.0.1)	Lenth et al.	https://cran.r-project.org/web/packages/emmeans/index.html

Other		

MERSCOPE spatial transcriptomics platform	Vizgen	https://vizgen.com
Illumina sequencers (NextSeq 550, NovaSeq 6000, NovaSeq X)	Illumina	N/A

## Highlights

- Longitudinal multi-omic profiling of motor neurons in SOD1-ALS mice
- Vulnerable alpha motor neurons adopt a disease-associated motor neuron state (DM)
- Transcription factor networks govern motor neuron state transitions
- DM features are conserved in human ALSt
