# Supplementary material for: An emergent disease-associated motor neuron state precedes cell death in ALS
Source: Cell. Author manuscript; Available in PMC 2026 Aug 7. (PMC13446465; doi:10.1016/j.cell.2026.05.047)
Supplement: Supplementary file 1 [file NIHMS2184328-supplement-MMC1.pdf]

Cell, Volume *189*

Supplemental information

An emergent disease-associated motor neuron state precedes cell death in ALS

Olivia Gautier, Jacob A. Blum, Thao P. Nguyen, Shaolong Cao, Sandy Klemm, Mai Yamakawa, Dann Huh, Jessica A. Hurt, Nasa Sinnott-Armstrong, Yi Zeng, Chung-ha O. Davis, Juliane Bombosch, Chang Liu, Lisa N. Encarnacion, Kevin A. Guttenplan, Derek Chen, Arwa Kathiria, Luke Zhao, Stephen Moore, Alex Meng, Kailee Ong, Don W. Cleveland, John Ravits, Jessica E. Rexach, William J. Greenleaf, and Aaron D. Gitler

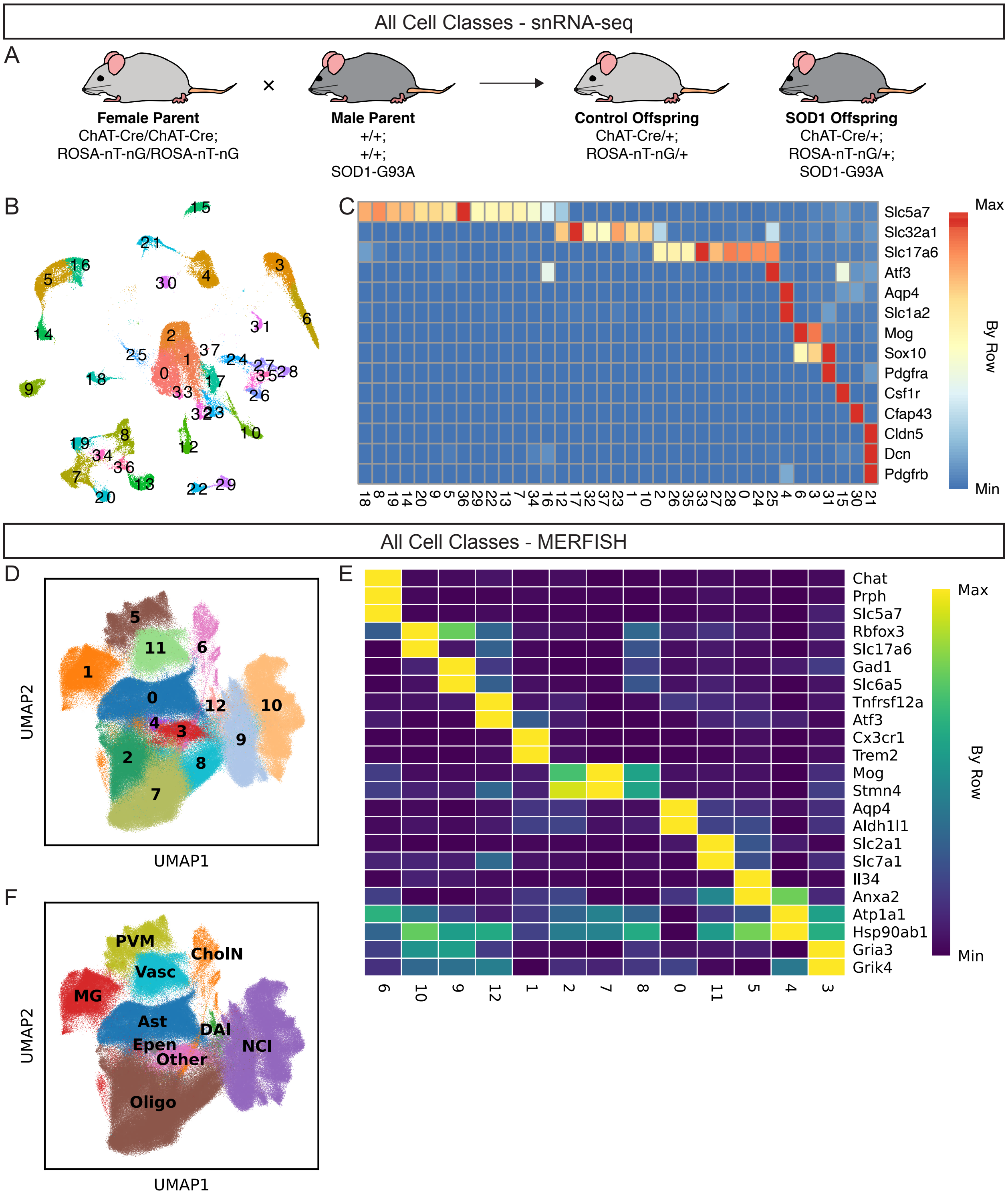
All Cell Classes - snRNA-seq
A
Female Parent
ChAT-Cre/ChAT-Cre;
ROSA-nT-nG/ROSA-nT-nG
×
Male Parent
+/+;
+/+;
SOD1-G93A
Control Offspring
ChAT-Cre/+;
ROSA-nT-nG/+
SOD1 Offspring
ChAT-Cre/+;
ROSA-nT-nG/+;
SOD1-G93A
B
C
Slc5a7
Slc32a1
Slc17a6
Atf3
Aqp4
Slc1a2
Mog
Sox10
Pdgfra
Csf1r
Cfap43
Cldn5
Dcn
Pdgfrb
Max
By Row
Min
18 8 19 14 20 9 5 36 29 22 13 7 34 16 12 17 32 37 23 1 10 2 26 35 33 27 28 0 24 25 4 6 3 31 15 30 21
All Cell Classes - MERFISH
D
UMAP2
UMAP1
E
Chat
Prph
Slc5a7
Rbfox3
Slc17a6
Gad1
Slc6a5
Tnfrsf12a
Atf3
Cx3cr1
Trem2
Mog
Stmn4
Aqp4
Aldh1l1
Slc2a1
Slc7a1
Il34
Anxa2
Atp1a1
Hsp90ab1
Gria3
Grik4
Max
By Row
Min
6 10 9 12 1 2 7 8 0 11 5 4 3
F
PVM
CholN
Vasc
MG
Ast
DAI
Epen
NCI
Other
Oligo
UMAP2
UMAP1

Figure S1. Identification of major cell classes by snRNA-seq and MERFISH, related to Figure 1
(A) Mouse breeding scheme. **(B)** UMAP of all snRNA-seq nuclei labeled by cluster. **(C)** Expression heatmap of marker genes for each cluster from (B). **(D)** UMAP of MERFISH cells labeled by cluster. **(E)** Expression heatmap of marker genes for each cluster from (D). **(F)** UMAP of MERFISH cells labeled by major cell class (Ast: Astrocytes, CholN: Cholinergic Neurons, DAI: Disease-Associated Interneurons, MG: Microglia/Macrophages, NCI: Non-Cholinergic Interneurons, Oligo: Oligodendrocytes, Epen: Putative Ependymal Cells, PVM: Putative Perivascular/Meningeal Cells, Vasc: Putative Vascular Cells).

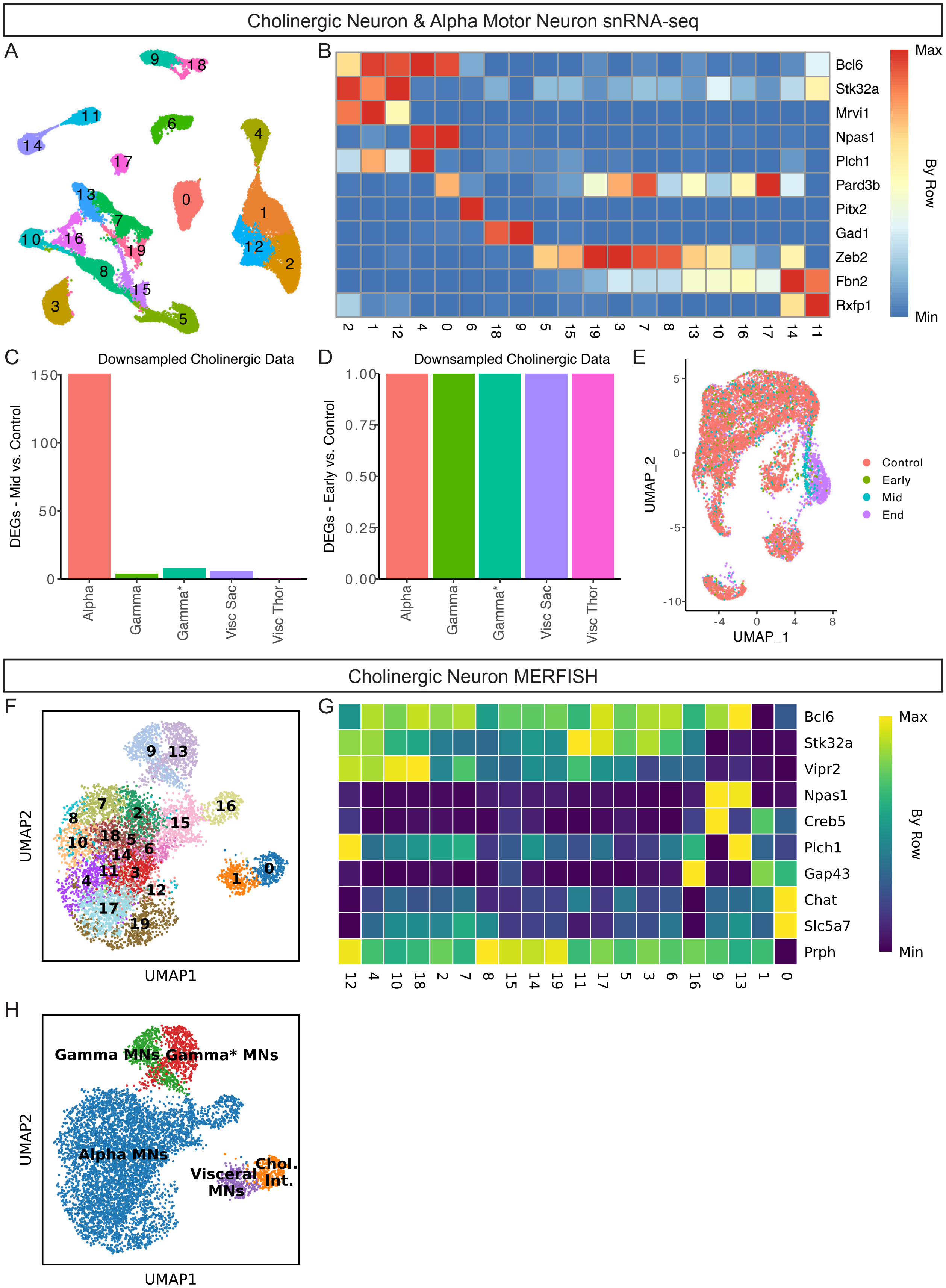

Cholinergic Neuron & Alpha Motor Neuron snRNA-seq
A
9
18
11
14
6
4
17
0
13
7
1
10
16
19
12
2
8
3
15
5
B
Bcl6
Stk32a
Mrvi1
Npas1
Plch1
Pard3b
Pitx2
Gad1
Zeb2
Fbn2
Rxfp1
Max
By Row
Min
2 1 12 4 0 6 18 9 5 15 19 3 7 8 13 10 16 17 14 11
C
Downsampled Cholinergic Data
DEGs - Mid vs. Control
150
100
50
0
Alpha
Gamma
Gamma*
Visc Sac
Visc Thor
D
Downsampled Cholinergic Data
DEGs - Early vs. Control
1.00
0.75
0.50
0.25
0.00
Alpha
Gamma
Gamma*
Visc Sac
Visc Thor
E
UMAP_2
UMAP_1
5
0
-5
-10
-4
0
4
8
Control
Early
Mid
End
Cholinergic Neuron MERFISH
F
UMAP2
UMAP1
9
13
16
7
2
8
15
10
18
5
6
14
11
3
1
0
4
12
17
19
G
Bcl6
Stk32a
Vipr2
Npas1
Creb5
Plch1
Gap43
Chat
Slc5a7
Prph
Max
By Row
Min
12 4 10 18 2 7 8 15 14 19 11 17 5 3 6 16 9 13 1 0
H
UMAP2
UMAP1
Gamma MNs
Gamma* MNs
Alpha MNs
Visceral MNs
Chol. Int.

Figure S2. Identification of cholinergic neuron types by snRNA-seq and MERFISH, related to Figures 1-2

(A) UMAP of snRNA-seq cholinergic neurons labeled by cluster. **(B)** Expression heatmap of marker genes for each cluster from (A). **(C)** Number of DEGs between mid-stage SOD1-G93A and control nuclei for each motor neuron type (DESeq2 Wald test; padj < 0.01). **(D)** Number of DEGs between early-stage SOD1-G93A and control nuclei for each motor neuron type (DESeq2 Wald test; padj < 0.01). The only DEG for all subtypes is the hSOD1-G93A transgene. **(E)** UMAP of snRNA-seq alpha motor neurons labeled by control and disease stage. **(F)** UMAP of MERFISH cholinergic neurons labeled by cluster. **(G)** Expression heatmap of marker genes for each cluster from (F). **(H)** UMAP of MERFISH cholinergic neurons labeled by cholinergic neuron type.

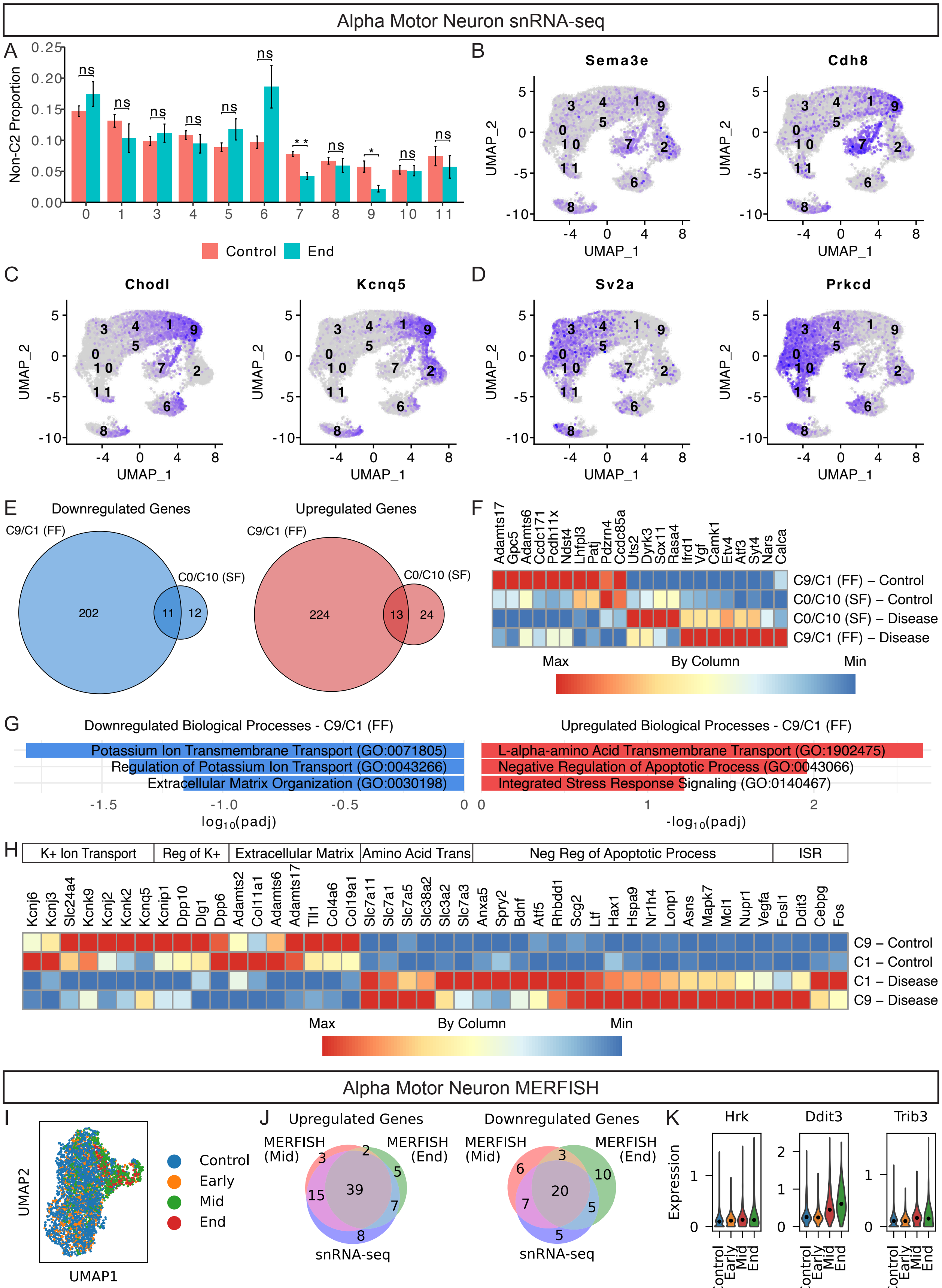
Alpha Motor Neuron snRNA-seq
A
Non-C2 Proportion
ns
ns
ns
ns
ns
ns
**
ns
*
ns
ns
Control
End
B
Sema3e
Cdh8
UMAP_2
UMAP_1
C
Chodl
Kcnq5
D
Sv2a
Prkcd
E
Downregulated Genes
C9/C1 (FF)
C0/C10 (SF)
202
11
12
Upregulated Genes
224
13
24
F
Adamts17
Gpc5
Adamts6
Ccdc171
Pcdh11x
Ndst4
Lhfpl3
Patj
Pdzrn4
Ccdc85a
Uts2
Dyrk3
Sox11
Rasa4
Ifrd1
Vgf
Camk1
Etv4
Atf3
Syt4
Nars
Calca
C9/C1 (FF) – Control
C0/C10 (SF) – Control
C0/C10 (SF) – Disease
C9/C1 (FF) – Disease
Max
By Column
Min
G
Downregulated Biological Processes - C9/C1 (FF)
Potassium Ion Transmembrane Transport (GO:0071805)
Regulation of Potassium Ion Transport (GO:0043266)
Extracellular Matrix Organization (GO:0030198)
log10(padj)
Upregulated Biological Processes - C9/C1 (FF)
L-alpha-amino Acid Transmembrane Transport (GO:1902475)
Negative Regulation of Apoptotic Process (GO:0043066)
Integrated Stress Response Signaling (GO:0140467)
-log10(padj)
H
K+ Ion Transport
Reg of K+
Extracellular Matrix
Amino Acid Trans
Neg Reg of Apoptotic Process
ISR
Kcnj6
Kcnj3
Slc24a4
Kcnk9
Kcnj2
Kcnk2
Kcnq5
Kcnip1
Dpp10
Dlg1
Dpp6
Adamts2
Col11a1
Adamts6
Adamts17
Tll1
Col4a6
Col19a1
Slc7a11
Slc7a1
Slc7a5
Slc38a2
Slc7a3
Anxa5
Spry2
Bdnf
Atf5
Rhbdd1
Scg2
Ltf
Hax1
Hspa9
Nr1h4
Lonp1
Asns
Mapk7
Mcl1
Nupr1
Vegfa
Fosl1
Ddit3
Cebpg
Fos
C9 – Control
C1 – Control
C1 – Disease
C9 – Disease
Max
By Column
Min
Alpha Motor Neuron MERFISH
I
UMAP2
UMAP1
Control
Early
Mid
End
J
Upregulated Genes
MERFISH (Mid)
MERFISH (End)
snRNA-seq
3
2
5
15
39
7
8
Downregulated Genes
6
3
10
20
7
5
5
K
Hrk
Ddit3
Trib3
Expression
Control
Early
Mid
End

Figure S3. Subtype-specific responses of alpha motor neurons to disease, related to Figure 2
(A) Proportions of non-cluster 2 alpha motor neurons by cluster in control and end-stage conditions (mean ± standard error across experiments; n = 11 control, n = 6 end-stage; Welch's t-tests with FDR correction; n.s. = not significant; * padj ≤ 0.05 and ** padj ≤ 0.01). **(B-D)** UMAPs showing expression of marker genes of gluteus/shoulder-innervating (B), fast-firing (C), and slow-firing (D) alpha motor neurons (darker purple = higher expression). **(E)** Overlap of downregulated (left) or upregulated (right) genes in pre-DM fast-firing (clusters 9 and 1) and slow-firing (clusters 0 and 10) alpha motor neurons (mid/end-stage versus control; DESeq2 Wald test; padj < 0.01). **(F)** Expression heatmap of selected DEGs in both fast-firing and slow-firing pre-DM alpha motor neurons. Median $|\log_2 FC|$ for control versus disease = 1.30 in C9/C1 (FF) neurons (range 0.66-4.85) and 1.02 in C0/C10 (SF) neurons (range 0.61-4.25). **(G)** Selected GO biological processes enriched among genes downregulated (left) and upregulated (right) in pre-DM fast-firing alpha motor neurons (Enrichr; Fisher's exact tests with FDR correction). **(H)** Expression heatmap of selected DEGs in pre-DM fast-firing alpha motor neurons. Median $|\log_2 FC|$ for C9/C1 (FF) control versus disease = 1.27 (range 0.63-3.58). **(I)** UMAP of MERFISH alpha motor neurons labeled by control and disease stage. **(J)** Overlap of upregulated (left) and downregulated (right) genes in alpha motor neurons across MERFISH and snRNA-seq datasets. MERFISH results compare mid- or end-stage alpha motor neurons to controls (two-sided Mann-Whitney U tests; FDR < 0.01). For snRNA-seq, only DEGs from Table S1A (DESeq2 Wald test; padj < 0.01) that are present in the MERFISH panel were included. **(K)** Log-normalized expression of select pro-death and stress-associated DEGs by MERFISH (two-sided Mann-Whitney U tests between control and end-stage; FDR < 0.01).

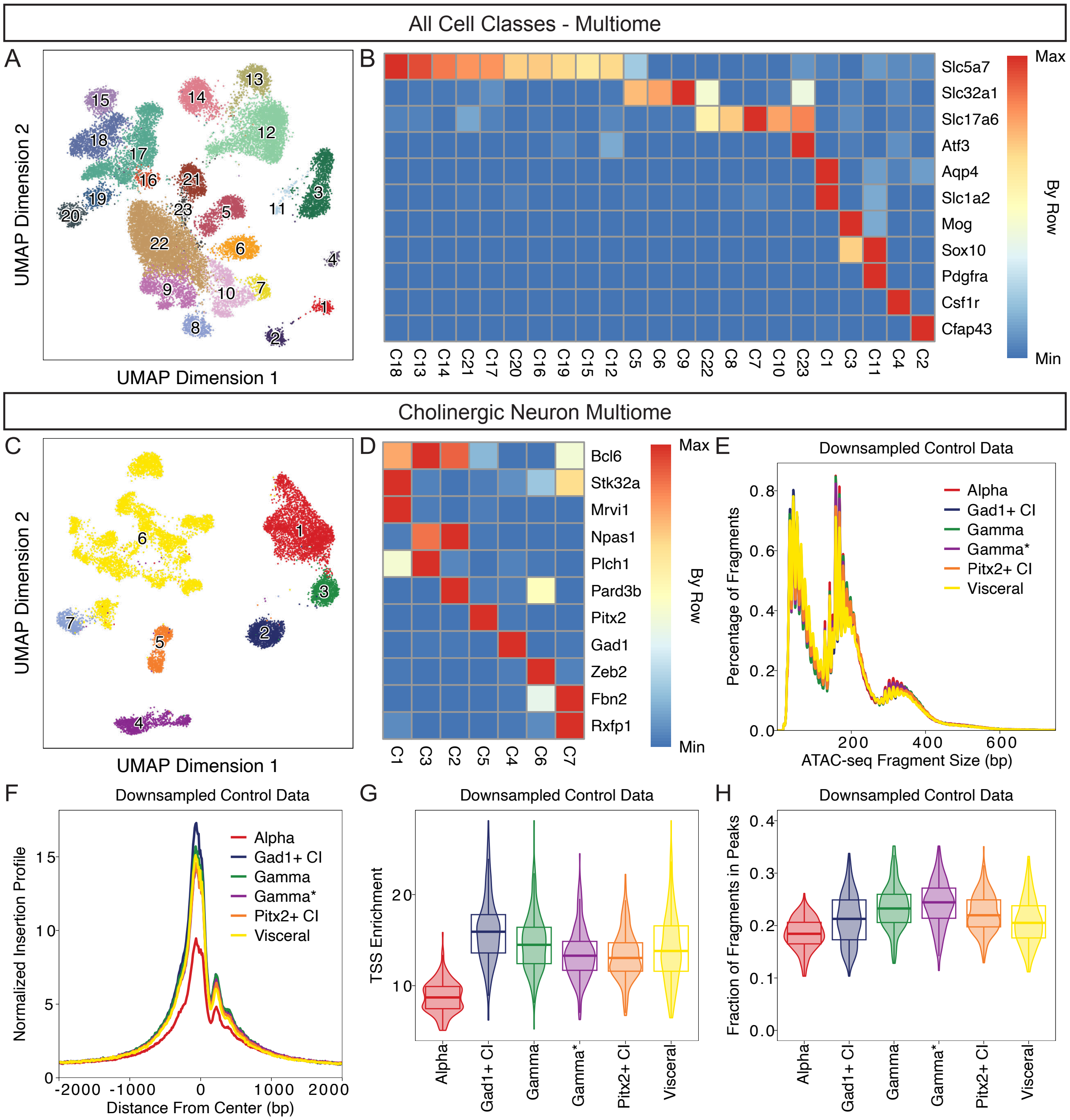
All Cell Classes - Multiome
A
UMAP Dimension 2
UMAP Dimension 1
B
Slc5a7
Slc32a1
Slc17a6
Atf3
Aqp4
Slc1a2
Mog
Sox10
Pdgfra
Csf1r
Cfap43
C18 C13 C14 C21 C17 C20 C16 C19 C15 C12 C5 C6 C9 C22 C8 C7 C10 C23 C1 C3 C11 C4 C2
Max
By Row
Min
Cholinergic Neuron Multiome
C
UMAP Dimension 2
UMAP Dimension 1
D
Bcl6
Stk32a
Mrvi1
Npas1
Plch1
Pard3b
Pitx2
Gad1
Zeb2
Fbn2
Rxfp1
C1 C3 C2 C5 C4 C6 C7
Max
By Row
Min
E
Downsampled Control Data
Percentage of Fragments
ATAC-seq Fragment Size (bp)
Alpha
Gad1+ CI
Gamma
Gamma*
Pitx2+ CI
Visceral
F
Downsampled Control Data
Normalized Insertion Profile
Distance From Center (bp)
Alpha
Gad1+ CI
Gamma
Gamma*
Pitx2+ CI
Visceral
G
Downsampled Control Data
TSS Enrichment
Alpha
Gad1+ CI
Gamma
Gamma*
Pitx2+ CI
Visceral
H
Downsampled Control Data
Fraction of Fragments in Peaks
Alpha
Gad1+ CI
Gamma
Gamma*
Pitx2+ CI
Visceral

Figure S4. Identification of major cell classes and cholinergic neuron types by paired snATAC-seq and snRNA-seq, related to Figure 3

(A) UMAP of all nuclei from integrated paired snATAC-seq and snRNA-seq data labeled by integrated RNA/ATAC-derived cluster. **(B)** Expression heatmap of marker genes for each cluster from (A). **(C)** UMAP of cholinergic neurons from integrated paired snATAC-seq and snRNA-seq data labeled by ATAC-derived cluster. **(D)** Expression heatmap of marker genes for each cluster from (C). **(E)** snATAC-seq fragment size distributions for each control cholinergic type. **(F)** snATAC-seq TSS enrichment profiles for each control cholinergic type. **(G)** Distribution of snATAC-seq TSS enrichment scores across control cholinergic neuron types. **(H)** Distribution of snATAC-seq Fraction of Reads/Fragments in Peaks (FRIP) scores across control cholinergic neuron types. FRIP was calculated separately for each cholinergic neuron type using the sub-type-specific peak sets from Figure 3E.

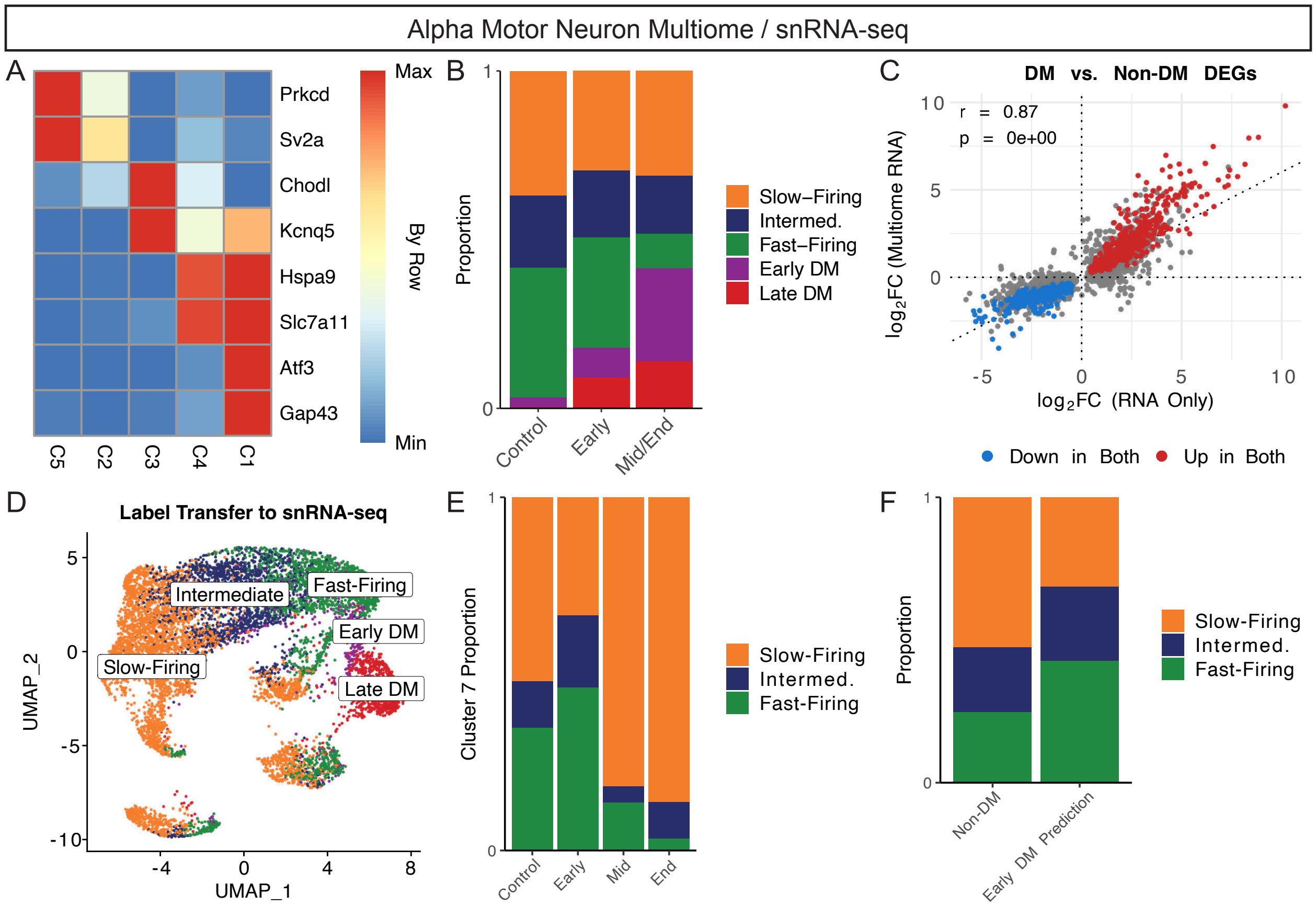

Alpha Motor Neuron Multiome / snRNA-seq
A
Prkcd
Sv2a
Chodl
Kcnq5
Hspa9
Slc7a11
Atf3
Gap43
C5
C2
C3
C4
C1
Max
By Row
Min
B
Proportion
Control
Early
Mid/End
Slow–Firing
Intermed.
Fast–Firing
Early DM
Late DM
C
DM vs. Non-DM DEGs
r = 0.87
p = 0e+00
log2FC (Multiome RNA)
log2FC (RNA Only)
Down in Both
Up in Both
D
Label Transfer to snRNA-seq
Intermediate
Fast-Firing
Early DM
Slow-Firing
Late DM
UMAP_2
UMAP_1
E
Cluster 7 Proportion
Control
Early
Mid
End
Slow-Firing
Intermed.
Fast-Firing
F
Proportion
Non-DM
Early DM Prediction
Slow-Firing
Intermed.
Fast-Firing

Figure S5. Identification of alpha motor neuron subtypes by paired snATAC-seq and snRNA-seq and label transfer to snRNA-seq data, related to Figure 3

(A) Expression heatmap of marker genes for each alpha motor neuron cluster from Figure 3G. **(B)** Proportion of alpha motor neuron subtypes across control and disease stages. **(C)** Concordance of gene-level $\log_2$ fold changes for DM versus non-DM motor neurons between RNA-only (x-axis) and multiome RNA (y-axis). Genes significantly up- or downregulated in both datasets (DESeq2 Wald test; padj < 0.01) are shown in red and blue, respectively. Genes significant in only one dataset are shown in gray. Dotted line indicates linear regression fit. Pearson r and two-sided p-value are shown. **(D)** UMAP of snRNA-seq alpha motor neurons labeled by alpha motor neuron subtype after label transfer from the multiome data. **(E)** Proportion of non-DM alpha motor neuron subtypes within cluster 7 from snRNA-seq across control and disease stages. **(F)** Proportion of predicted non-DM alpha motor neuron subtypes for Early DM cells compared to the observed subtype composition in non-DM cells.

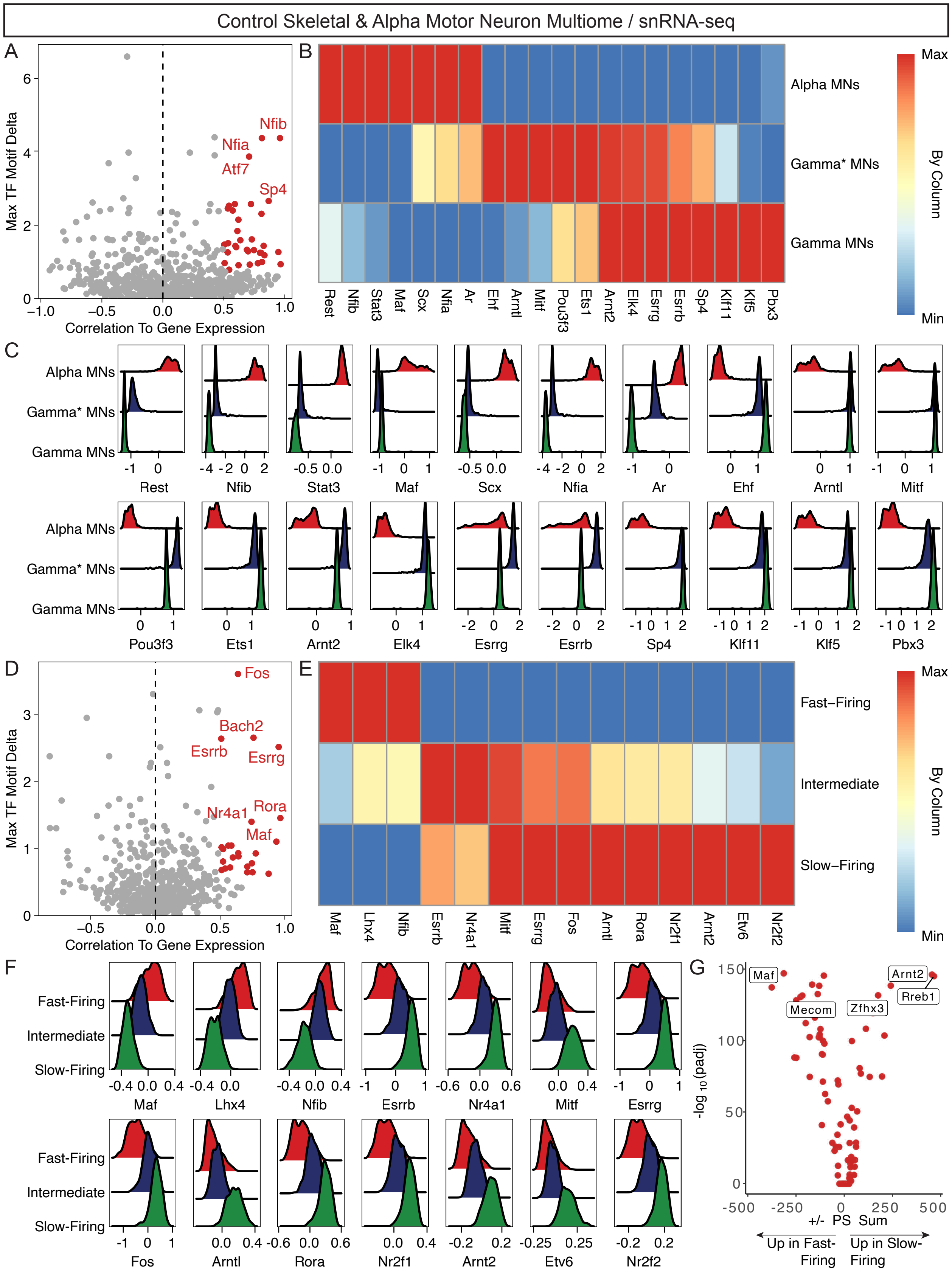

Control Skeletal & Alpha Motor Neuron Multiome / snRNA-seq
A
Max TF Motif Delta
Correlation To Gene Expression
Nfib
Nfia
Atf7
Sp4
B
Alpha MNs
Gamma* MNs
Gamma MNs
Max
Min
By Column
Rest
Nfib
Stat3
Maf
Scx
Nfia
Ar
Ehf
Arntl
Mitf
Pou3f3
Ets1
Arnt2
Elk4
Esrrg
Esrrb
Sp4
Klf11
Klf5
Pbx3
C
D
Fos
Bach2
Esrrb
Esrrg
Nr4a1
Rora
Maf
E
Fast–Firing
Intermediate
Slow–Firing
Lhx4
Nr2f1
Nr2f2
Etv6
F
G
Maf
Mecom
Zfhx3
Arnt2
Rreb1
-log 10 (padj)
+/- PS Sum
Up in Fast-Firing
Up in Slow-Firing

Figure S6. TF regulators of skeletal and alpha motor neuron subtypes, related to Figure 4
(A) ArchR identification of positive TF regulators of alpha, gamma*, and gamma motor neurons. TFs in the top quartile of maximum differences in chromVAR deviation z-scores with correlation > 0.5 are in red. **(B)** Expression heatmap of selected differentially expressed TFs between alpha and pan-gamma motor neurons by snRNA-seq (median $|\log_2 FC|$ for control alpha versus pan-gamma motor neurons = 1.18; range 0.55-6.91). **(C)** Distributions of chromVAR deviation scores of TFs from (B) for alpha, gamma*, and gamma motor neurons. **(D)** ArchR identification of positive TF regulators of alpha motor neuron subtypes (fast-firing, intermediate, slow-firing). TFs in the top quartile of maximum differences in chromVAR deviation z-scores with correlation > 0.5 are in red. **(E)** Expression heatmap of selected differentially expressed TFs between fast-firing and slow-firing alpha motor neurons by snRNA-seq after label transfer from the multiome data (median $|\log_2 FC|$ for control fast-firing versus slow-firing alpha motor neurons = 1.13; range 0.52-2.70). **(F)** Distributions of chromVAR deviation scores of TFs from (E) for fast-firing, intermediate, and slow-firing alpha motor neurons. **(G)** CellOracle *in silico* knockout results of 56 TFs in fast-firing and slow-firing alpha motor neurons (PS Sum = sum of perturbation scores across nuclei).

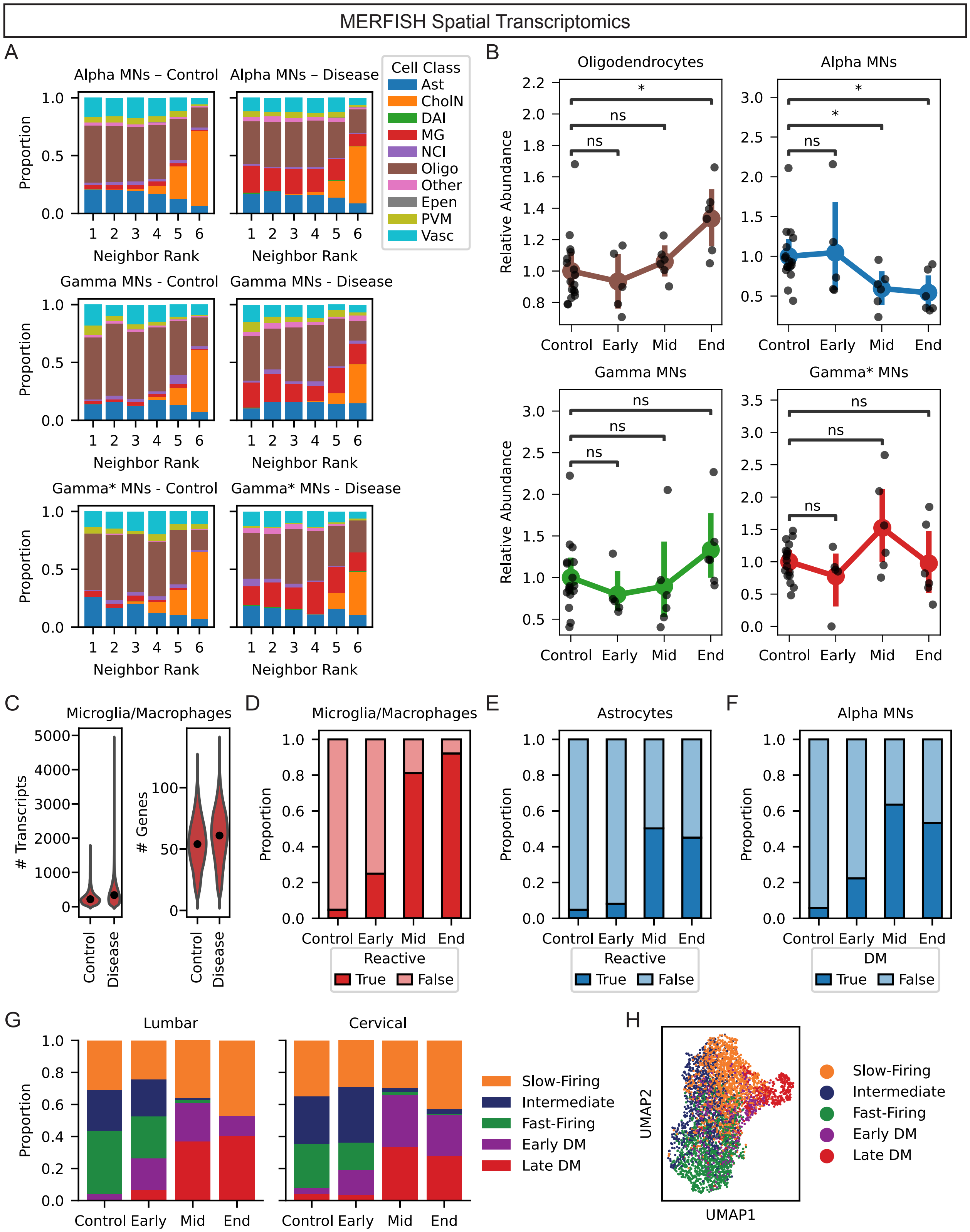

MERFISH Spatial Transcriptomics
A
Alpha MNs – Control
Alpha MNs – Disease
Gamma MNs - Control
Gamma MNs - Disease
Gamma* MNs - Control
Gamma* MNs - Disease
Proportion
Neighbor Rank
Cell Class
Ast
CholN
DAI
MG
NCI
Oligo
Other
Epen
PVM
Vasc
B
Oligodendrocytes
Alpha MNs
Gamma MNs
Gamma* MNs
Relative Abundance
ns
*
Control
Early
Mid
End
C
Microglia/Macrophages
Transcripts
Genes
Control
Disease
D
Microglia/Macrophages
Proportion
Reactive
True
False
E
Astrocytes
Reactive
True
False
F
Alpha MNs
DM
True
False
G
Lumbar
Cervical
Proportion
Slow-Firing
Intermediate
Fast-Firing
Early DM
Late DM
H
UMAP2
UMAP1
Slow-Firing
Intermediate
Fast-Firing
Early DM
Late DM

Figure S7. Extended spatial and compositional dynamics of spinal cord cell types with disease in the SOD1-G93A mouse, related to Figure 6

(A) Cell class composition of the six nearest neighbors to alpha (top), gamma (middle), and gamma* (bottom) motor neurons in control (left) and disease (right) conditions (Ast: Astrocytes, CholN: Cholinergic Neurons, DAI: Disease-Associated Interneurons, MG: Microglia/Macrophages, NCI: Non-Cholinergic Interneurons, Oligo: Oligodendrocytes, Epen: Putative Ependymal Cells, PVM: Putative Perivascular/Meningeal Cells, Vasc: Putative Vascular Cells). Microglia/macrophages are significantly enriched at all neighbor positions for all skeletal motor neuron subtypes in disease compared to control (Fisher's exact tests; Bonferroni-adjusted $p \leq 0.01$). **(B)** Abundance of oligodendrocytes (top left), alpha (top right), gamma (bottom left), and gamma* (bottom right) skeletal motor neurons relative to interneurons. Black points represent per-experiment means ($n \geq 5$), scaled to control (one-sided Welch's t-tests with Bonferroni correction; n.s. = not significant; * $p_{adj} \leq 0.05$). Colored points and lines indicate condition means with 95% confidence intervals. **(C)** Distribution of unique transcripts (left) and genes (right) detected per microglia/macrophage from control and disease conditions (median values = 218 transcripts and 54 genes in control, and 338 transcripts and 61 genes in disease). **(D)** Proportion of reactive and non-reactive microglia/macrophages across conditions. **(E)** Proportion of reactive/white matter and other astrocytes across conditions. **(F)** Proportion of DM and non-DM alpha motor neurons across conditions after label transfer. **(G)** Proportion of alpha motor neuron subtypes across conditions. **(H)** UMAP of MERFISH alpha motor neurons labeled by alpha motor neuron subtype.

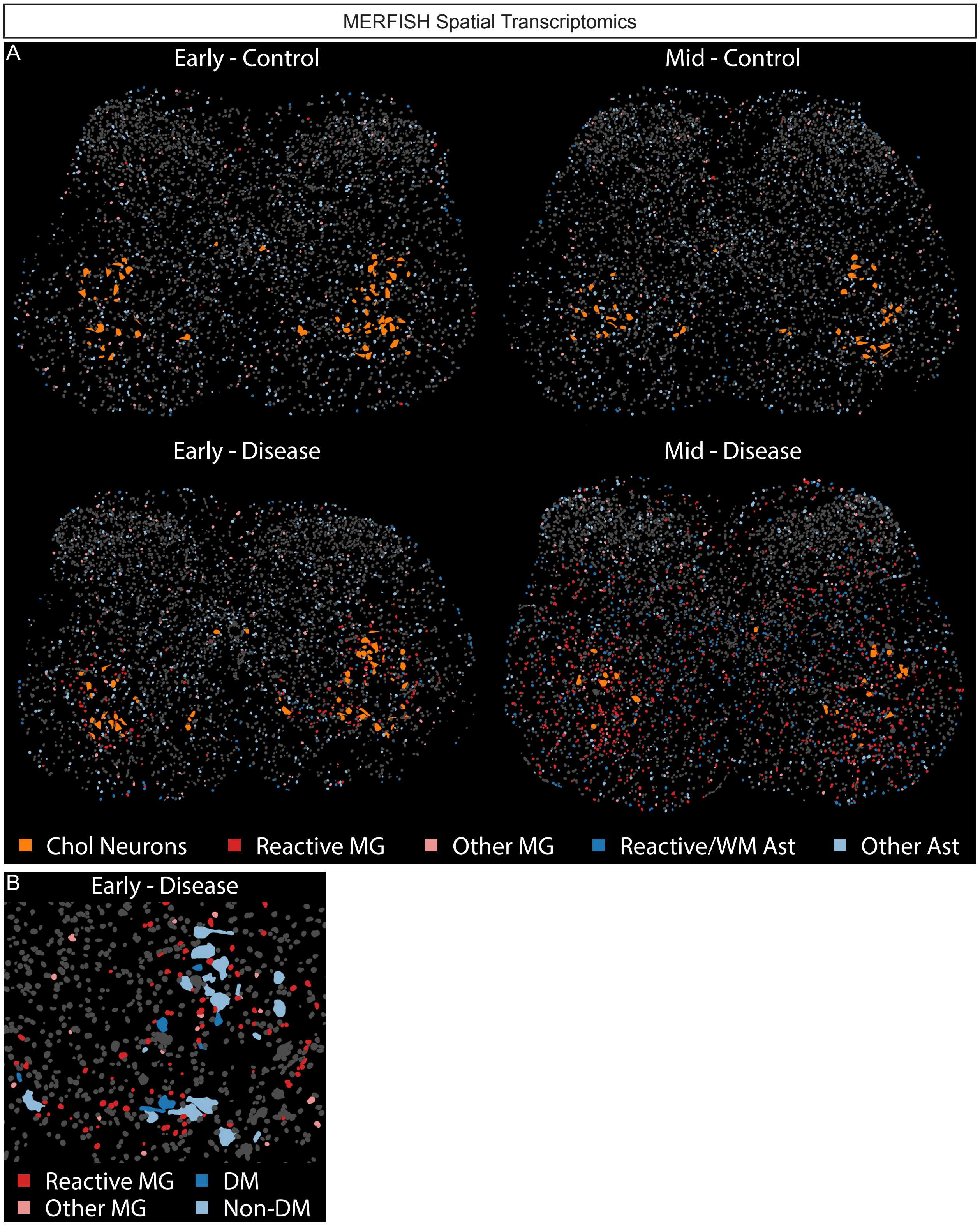
MERFISH Spatial Transcriptomics
A
Early - Control
Mid - Control
Early - Disease
Mid - Disease
Chol Neurons
Reactive MG
Other MG
Reactive/WM Ast
Other Ast
B
Early - Disease
Reactive MG
DM
Other MG
Non-DM

Figure S8. Representative MERFISH segmentation images, related to Figure 6
(A) Representative lumbar spinal cord sections from early- and mid-stage control and SOD1-G93A mice, showing cholinergic neurons (orange), reactive microglia/macrophages (red), other microglia (pink), reactive/white matter astrocytes (dark blue), and other astrocytes (light blue). **(B)** Higher-magnification view of the ventral horn from an early-stage disease section showing reactive microglia/macrophages (red), other microglia (pink), DM alpha motor neurons (dark blue), and non-DM alpha motor neurons (light blue).

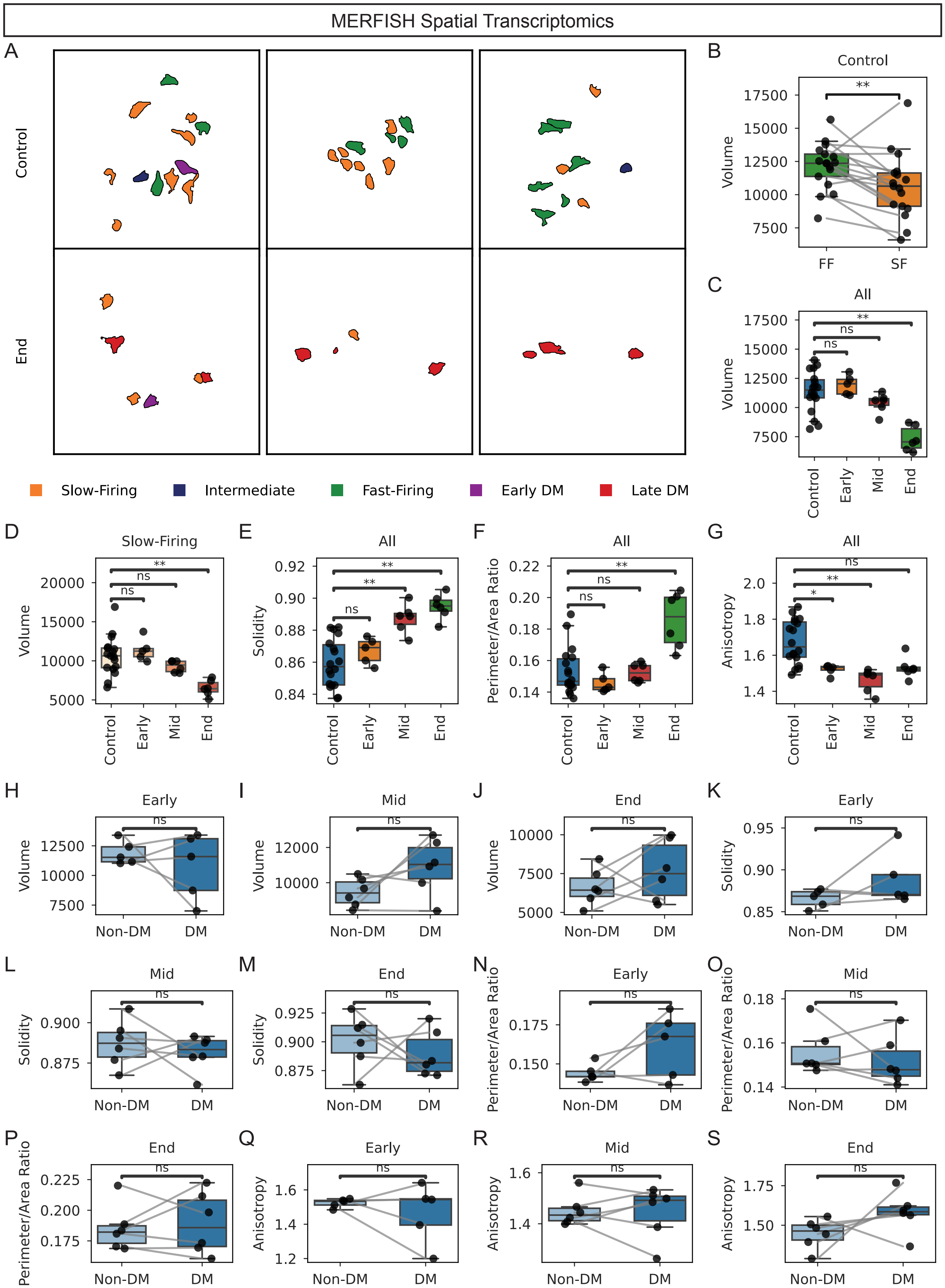

MERFISH Spatial Transcriptomics
A
Control
End
Slow-Firing
Intermediate
Fast-Firing
Early DM
Late DM
B
Control
Volume
FF
SF
C
All
Control
Early
Mid
End
D
Slow-Firing
E
All
Solidity
F
All
Perimeter/Area Ratio
G
All
Anisotropy
H
Early
I
Mid
J
End
K
Early
L
Mid
M
End
N
Early
O
Mid
P
End
Q
Early
R
Mid
S
End
Non-DM
DM
ns
*
**

Figure S9. MERFISH-based alpha motor neuron morphology across disease progression, related to Figure 6

(A) Representative segmentation masks of alpha motor neurons in control (top) and end-stage disease (bottom) MERFISH lumbar spinal cord sections. **(B)** Soma volume of control Fast-Firing (FF) and Slow-Firing (SF) alpha motor neurons in control samples. Points represent per-experiment means (n = 18; one-sided paired Wilcoxon signed-rank test; ** $p \leq 0.01$). **(C-D)** Soma volume of all alpha motor neurons (C) or slow-firing alpha motor neurons (D) across conditions. Points represent per-experiment means ($n \geq 5$; one-sided Mann–Whitney U tests with Bonferroni correction; n.s. = not significant; ** padj ≤ 0.01). **(E-G)** Alpha motor neuron morphometric features across conditions for solidity (E), perimeter-to-area ratio (F), and anisotropy (G). Points represent per-experiment means ($n \geq 5$; Mann–Whitney U tests with Bonferroni correction; n.s. = not significant; * padj ≤ 0.05 and ** padj ≤ 0.01). **(H-S)** Morphometric features between non-DM and DM alpha motor neurons within each disease stage (volume: H-J; solidity: K-M; perimeter-to-area ratio: N-P; anisotropy: Q-S). Points represent per-experiment means ($n \geq 5$; paired Wilcoxon signed-rank tests performed separately for each stage and metric with FDR correction applied across stages for each metric; n.s. = not significant).

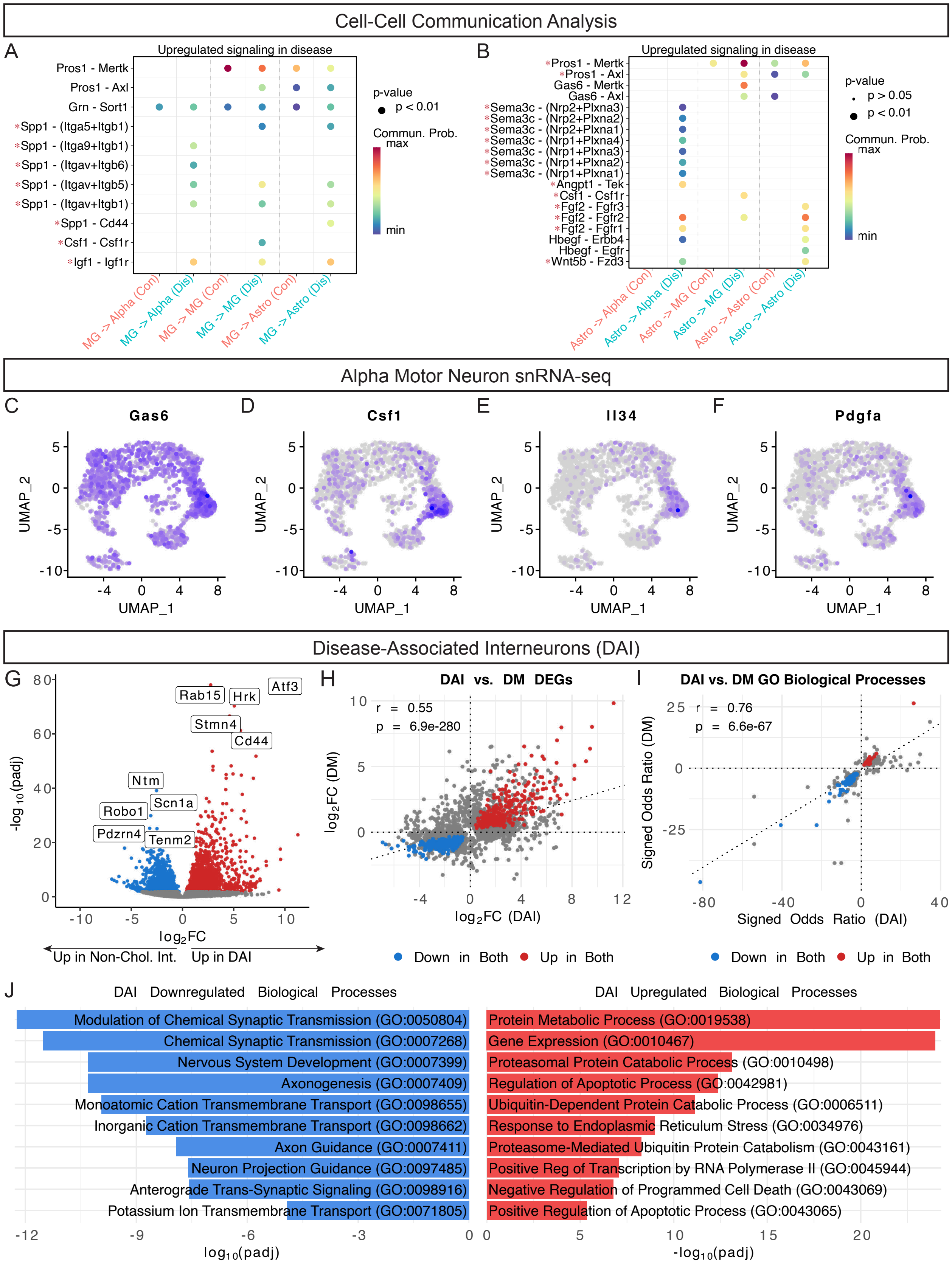
Cell-Cell Communication Analysis
A
Upregulated signaling in disease
Pros1 - Mertk
Pros1 - Axl
Grn - Sort1
*Spp1 - (Itga5+Itgb1)
*Spp1 - (Itga9+Itgb1)
*Spp1 - (Itgav+Itgb6)
*Spp1 - (Itgav+Itgb5)
*Spp1 - (Itgav+Itgb1)
*Spp1 - Cd44
*Csf1 - Csf1r
*Igf1 - Igf1r
MG -> Alpha (Con)
MG -> Alpha (Dis)
MG -> MG (Con)
MG -> MG (Dis)
MG -> Astro (Con)
MG -> Astro (Dis)
p-value
p < 0.01
Commun. Prob.
max
min
B
Upregulated signaling in disease
*Pros1 - Mertk
*Pros1 - Axl
Gas6 - Mertk
Gas6 - Axl
*Sema3c - (Nrp2+Plxna3)
*Sema3c - (Nrp2+Plxna2)
*Sema3c - (Nrp2+Plxna1)
*Sema3c - (Nrp1+Plxna4)
*Sema3c - (Nrp1+Plxna3)
*Sema3c - (Nrp1+Plxna2)
*Sema3c - (Nrp1+Plxna1)
*Angpt1 - Tek
*Csf1 - Csf1r
*Fgf2 - Fgfr3
*Fgf2 - Fgfr2
*Fgf2 - Fgfr1
Hbegf - Erbb4
Hbegf - Egfr
*Wnt5b - Fzd3
Astro -> Alpha (Con)
Astro -> Alpha (Dis)
Astro -> MG (Con)
Astro -> MG (Dis)
Astro -> Astro (Con)
Astro -> Astro (Dis)
p-value
p > 0.05
p < 0.01
Commun. Prob.
max
min
Alpha Motor Neuron snRNA-seq
C
Gas6
D
Csf1
E
Il34
F
Pdgfa
UMAP_2
UMAP_1
Disease-Associated Interneurons (DAI)
G
Rab15
Hrk
Atf3
Stmn4
Cd44
Ntm
Scn1a
Robo1
Pdzrn4
Tenm2
-log10(padj)
log2FC
Up in Non-Chol. Int.
Up in DAI
H
DAI vs. DM DEGs
r = 0.55
p = 6.9e-280
log2FC (DM)
log2FC (DAI)
Down in Both
Up in Both
I
DAI vs. DM GO Biological Processes
r = 0.76
p = 6.6e-67
Signed Odds Ratio (DM)
Signed Odds Ratio (DAI)
Down in Both
Up in Both
J
DAI Downregulated Biological Processes
Modulation of Chemical Synaptic Transmission (GO:0050804)
Chemical Synaptic Transmission (GO:0007268)
Nervous System Development (GO:0007399)
Axonogenesis (GO:0007409)
Monoatomic Cation Transmembrane Transport (GO:0098655)
Inorganic Cation Transmembrane Transport (GO:0098662)
Axon Guidance (GO:0007411)
Neuron Projection Guidance (GO:0097485)
Anterograde Trans-Synaptic Signaling (GO:0098916)
Potassium Ion Transmembrane Transport (GO:0071805)
log10(padj)
DAI Upregulated Biological Processes
Protein Metabolic Process (GO:0019538)
Gene Expression (GO:0010467)
Proteasomal Protein Catabolic Process (GO:0010498)
Regulation of Apoptotic Process (GO:0042981)
Ubiquitin-Dependent Protein Catabolic Process (GO:0006511)
Response to Endoplasmic Reticulum Stress (GO:0034976)
Proteasome-Mediated Ubiquitin Protein Catabolism (GO:0043161)
Positive Reg of Transcription by RNA Polymerase II (GO:0045944)
Negative Regulation of Programmed Cell Death (GO:0043069)
Positive Regulation of Apoptotic Process (GO:0043065)
-log10(padj)

Figure S10. Candidate disease-associated ligand-receptor signaling and transcriptional signature of disease-associated interneurons (DAIs), related to Figure 6

(A-B) Ligand-receptor interactions upregulated in disease with microglia/macrophages (A) and astrocytes (B) as the sender cell types. Bubble size indicates statistical significance (permutation test), and color reflects communication probability (see STAR Methods). Red stars indicate ligand-receptor interactions where the ligand is significantly upregulated in microglia/macrophages or astrocytes, respectively, at end-stage by snRNA-seq (DESeq2 Wald test; padj < 0.01). **(C-F)** UMAPs showing expression of upregulated ligands in DMs (darker purple = higher expression). **(G)** DEGs in DAIs versus other non-cholinergic interneurons from multiome RNA (DESeq2 Wald test; padj < 0.01). **(H)** Concordance of gene-level $\log_2$ fold changes for DAI versus non-cholinergic interneurons (x-axis) and DM versus non-DM motor neurons (y-axis). Genes significantly up- or downregulated in both datasets (DESeq2 Wald test; padj < 0.01) are shown in red and blue, respectively. Genes significant in only one dataset are shown in gray. Dotted line indicates linear regression fit. Pearson r and two-sided p-value are shown. **(I)** Concordance of GO biological process enrichment between DAI and DM signatures (x-axis: DAI; y-axis: DM), plotted as signed odds ratios. Terms significantly enriched in both datasets (Enrichr; Fisher's exact tests with FDR correction; padj < 0.01) in the same direction are shown in red (positive) and blue (negative). Terms significant in only one dataset are shown in gray. Dotted line indicates linear regression fit. Pearson r and two-sided p-value are shown. **(J)** Selected GO biological processes enriched among DAI-downregulated (left) and DAI-upregulated (right) genes (Enrichr; Fisher's exact tests with FDR correction).

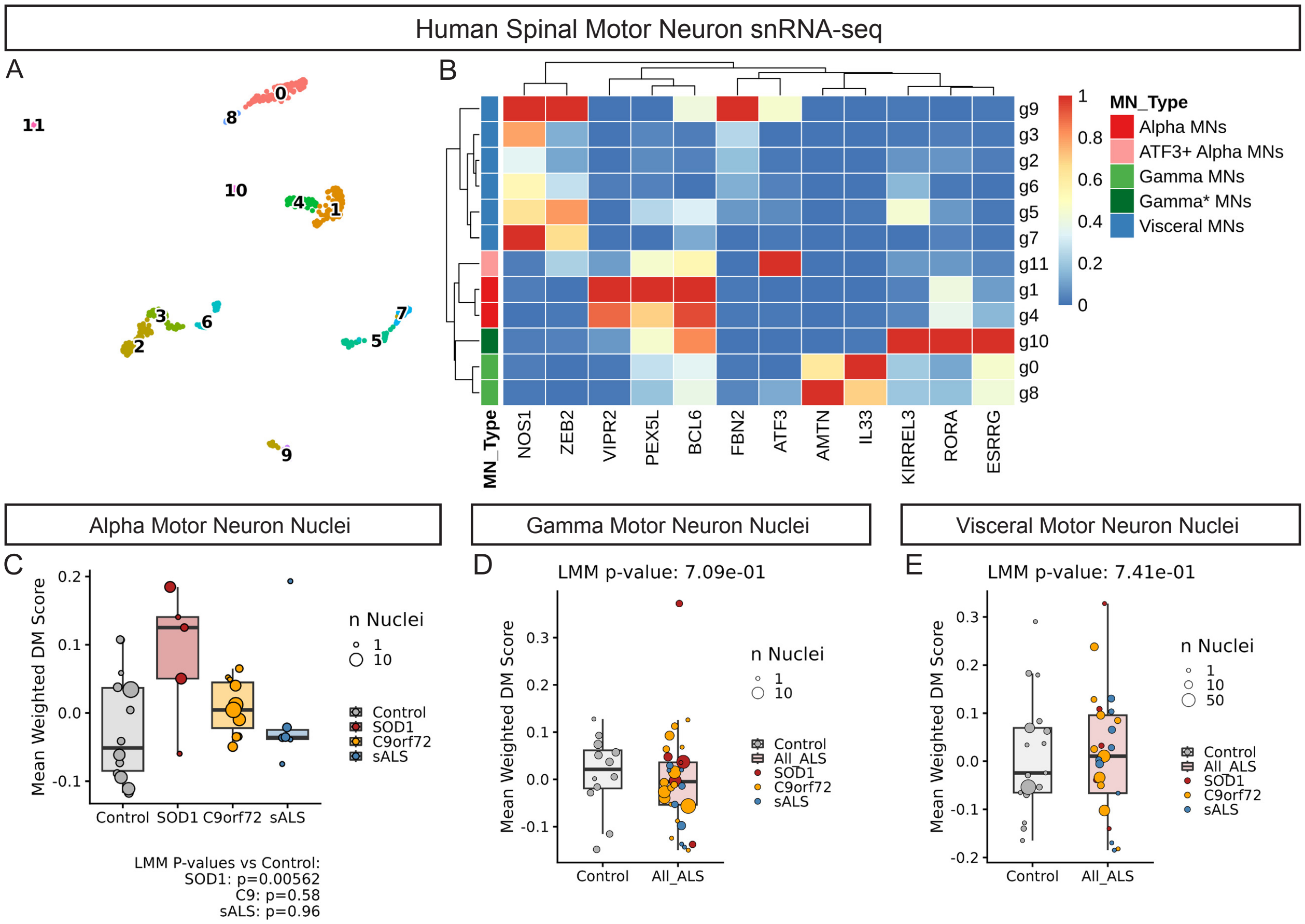

Human Spinal Motor Neuron snRNA-seq
A
B
MN_Type
Alpha MNs
ATF3+ Alpha MNs
Gamma MNs
Gamma* MNs
Visceral MNs
NOS1
ZEB2
VIPR2
PEX5L
BCL6
FBN2
ATF3
AMTN
IL33
KIRREL3
RORA
ESRRG
Alpha Motor Neuron Nuclei
Gamma Motor Neuron Nuclei
Visceral Motor Neuron Nuclei
C
D
E
LMM p-value: 7.09e-01
LMM p-value: 7.41e-01
Mean Weighted DM Score
n Nuclei
Control
SOD1
C9orf72
sALS
All_ALS
LMM P-values vs Control:
SOD1: p=0.00562
C9: p=0.58
sALS: p=0.96

Figure S11. Identification of human spinal motor neuron subtypes and subtype-specific transcriptional responses by snRNA-seq, related to Figure 7

(A) UMAP of human spinal motor neuron snRNA-seq nuclei labeled by cluster. **(B)** Expression heatmap of selected marker genes for each cluster in (A). Data are min/max-normalized by column. **(C-E)** DM scores from alpha (C), gamma (D), and visceral (E) motor neuron nuclei. Alpha motor neuron scores are shown across control and ALS subtypes, and gamma and visceral motor neuron scores are shown across control and pan-ALS conditions. Points represent sample/donor means. Statistical significance was assessed using linear mixed models fit to individual nuclei with sample identity as a random effect (see STAR Methods).

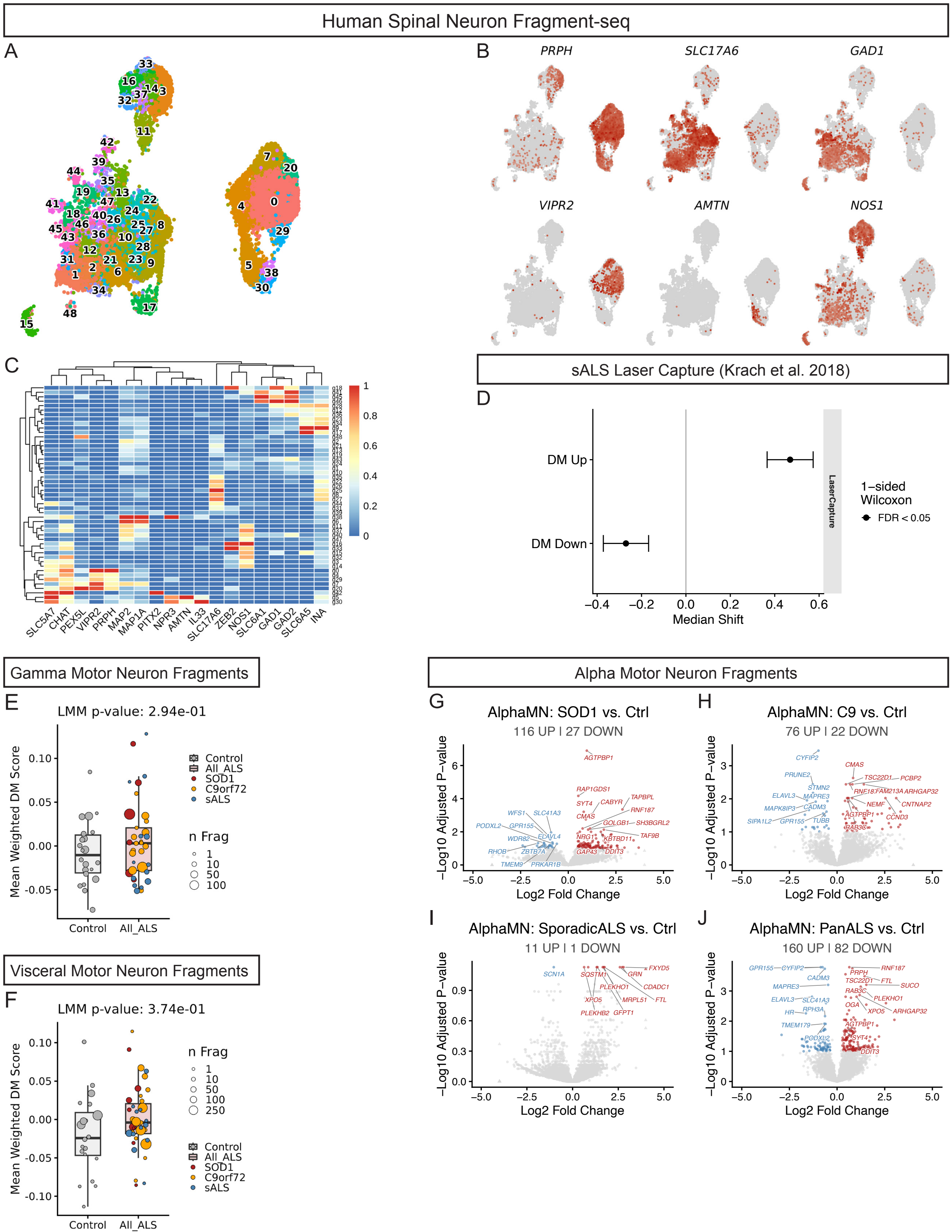
Human Spinal Neuron Fragment-seq
A
B
PRPH
SLC17A6
GAD1
VIPR2
AMTN
NOS1
C
sALS Laser Capture (Krach et al. 2018)
D
DM Up
DM Down
LaserCapture
1-sided Wilcoxon
FDR < 0.05
Median Shift
Gamma Motor Neuron Fragments
E
LMM p-value: 2.94e-01
Mean Weighted DM Score
Control
All_ALS
SOD1
C9orf72
sALS
n Frag
Visceral Motor Neuron Fragments
F
LMM p-value: 3.74e-01
Alpha Motor Neuron Fragments
G
AlphaMN: SOD1 vs. Ctrl
116 UP | 27 DOWN
H
AlphaMN: C9 vs. Ctrl
76 UP | 22 DOWN
I
AlphaMN: SporadicALS vs. Ctrl
11 UP | 1 DOWN
J
AlphaMN: PanALS vs. Ctrl
160 UP | 82 DOWN
-Log10 Adjusted P-value
Log2 Fold Change

Figure S12. Identification of human spinal motor neuron subtypes and subtype-specific transcriptional responses by neuronal fragment sequencing, related to Figure 7

(A) UMAP of human neuron fragment profiles labeled by cluster. **(B)** UMAPs showing expression of motor (*PRPH*), excitatory (*SLC17A6*), inhibitory (*GAD1*), alpha motor (*VIPR2*), gamma motor (*AMTN*), and visceral motor (*NOS1*) neuron marker genes (darker red = higher expression). **(C)** Expression heatmap of selected marker genes for each cluster in (A). Data are min/max-normalized by column. **(D)** Wilcoxon rank-based gene set enrichment of DM-up and DM-down gene sets in sALS versus control laser-captured motor neurons (Krach et al. 2018). Points indicate median shifts in DESeq2 Wald statistics for each gene set relative to all other genes tested (Hodges-Lehmann estimates with 95% confidence intervals). Significance was assessed by one-sided Wilcoxon-Mann-Whitney rank-sum tests with FDR correction across tested comparisons. **(E-F)** DM scores from gammaFRAGs (E) and visceralFRAGs (F) across control and pan-ALS conditions. Points represent sample/donor means. Significance was assessed using linear mixed models fit to individual fragments with sample identity as a random effect (see STAR Methods). **(G-J)** DEGs in SOD1-ALS (G), C9-ALS (H), sALS (I), and pan-ALS (J) alphaFRAGs relative to controls (NEBULA differential expression analysis; padj < 0.01).

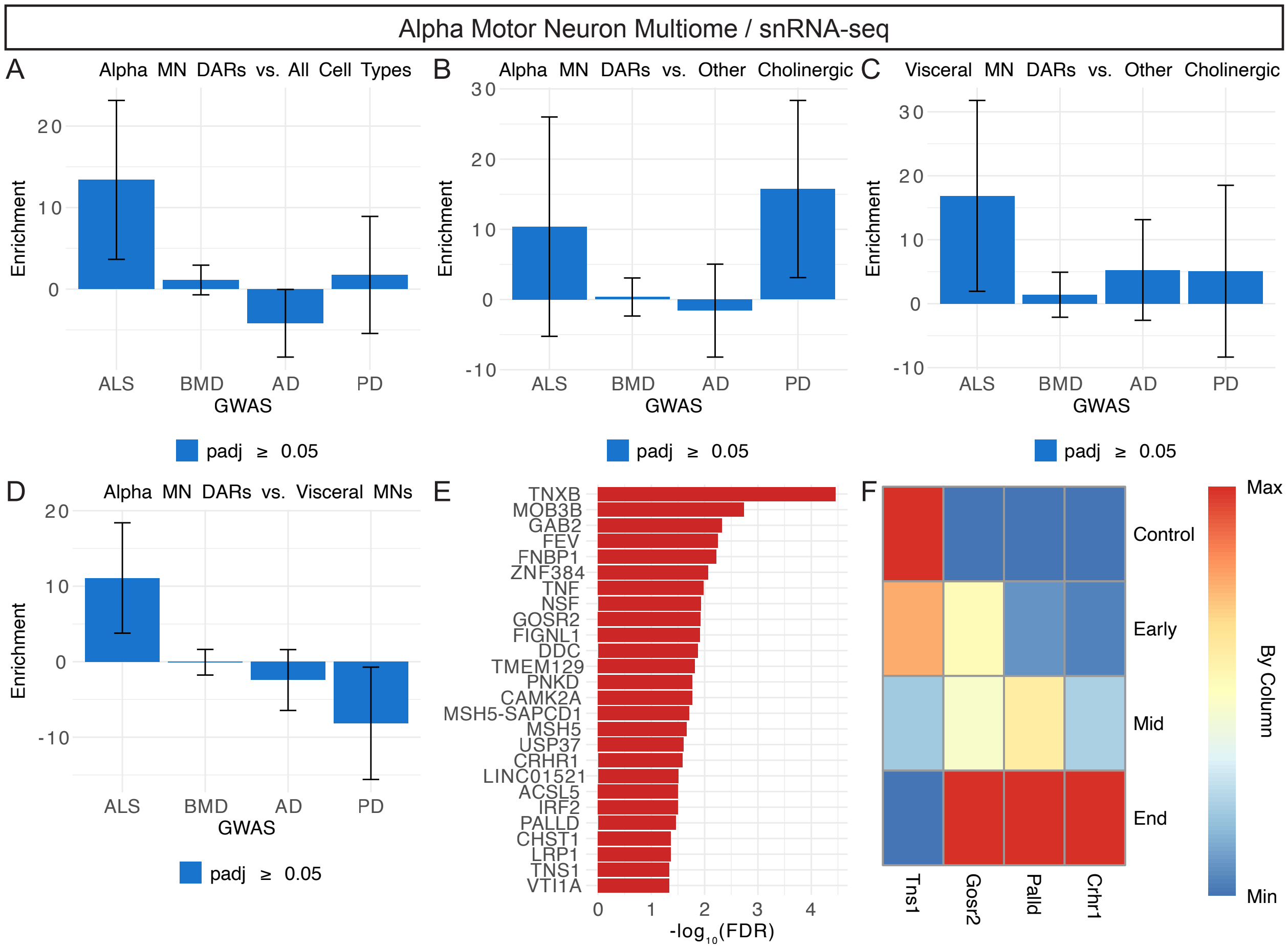
Alpha Motor Neuron Multiome / snRNA-seq
A
Alpha MN DARs vs. All Cell Types
B
Alpha MN DARs vs. Other Cholinergic
C
Visceral MN DARs vs. Other Cholinergic
Enrichment
ALS
BMD
AD
PD
GWAS
padj ≥ 0.05
D
Alpha MN DARs vs. Visceral MNs
E
TNXB
MOB3B
GAB2
FEV
FNBP1
ZNF384
TNF
NSF
GOSR2
FIGNL1
DDC
TMEM129
PNKD
CAMK2A
MSH5-SAPCD1
MSH5
USP37
CRHR1
LINC01521
ACSL5
IRF2
PALLD
CHST1
LRP1
TNS1
VTI1A
-log10(FDR)
F
Control
Early
Mid
End
Tns1
Gosr2
Palld
Crhr1
Max
Min
By Column

Figure S13. ALS heritability analyses and H-MAGMA prioritization of candidate genes in alpha motor neurons, related to Figure 7

(A-D) Heritability enrichment from partitioned LD score regression across four traits using differentially accessible regions (DARs) from the control condition. Traits include ALS, BMD (bone mineral density abnormality), AD (Alzheimer's disease), and PD (Parkinson's disease). Comparisons include alpha motor neuron (MN) DARs versus all cell types (A), alpha MN DARs versus other cholinergic neurons (B), visceral MN DARs versus other cholinergic neurons (C), and alpha MN DARs versus visceral MNs (D). Bars show enrichment estimates ± standard error and statistical significance (Z-tests on regression coefficients with FDR correction across tested traits). **(E)** Candidate ALS-associated genes based on H-MAGMA analysis and alpha motor neuron differential chromatin accessibility with disease. Gene-level associations were derived by mapping non-coding single nucleotide polymorphisms (SNPs) to target genes using chromatin interaction profiles (see STAR Methods). **(F)** Expression heatmap of candidate ALS-associated genes from (E) that are differentially expressed between control and end-stage alpha motor neurons by snRNA-seq (DESeq2 Wald test; padj < 0.01).
